# Significant enhancement of fatty acid composition in seeds of the allohexaploid, *Camelina sativa*, using CRISPR/Cas9 gene editing

**DOI:** 10.1111/pbi.12663

**Published:** 2017-01-12

**Authors:** Wen Zhi Jiang, Isabelle M. Henry, Peter G. Lynagh, Luca Comai, Edgar B. Cahoon, Donald P. Weeks

**Affiliations:** ^1^ Department of Biochemistry and Center for Plant Science InnovationUniversity of NebraskaLincolnNEUSA; ^2^ Department of Plant Biology and UC Davis Genome CenterUniversity of CaliforniaDavisCAUSA

**Keywords:** *Camelina sativa*, gene editing, CRISPR/Cas9, allohexaploid, oleic acid, fatty acid composition

## Abstract

The CRISPR/Cas9 nuclease system is a powerful and flexible tool for genome editing, and novel applications of this system are being developed rapidly. Here, we used CRISPR/Cas9 to target the *FAD2* gene in *Arabidopsis thaliana* and in the closely related emerging oil seed plant, *Camelina sativa,* with the goal of improving seed oil composition. We successfully obtained Camelina seeds in which oleic acid content was increased from 16% to over 50% of the fatty acid composition. These increases were associated with significant decreases in the less desirable polyunsaturated fatty acids, linoleic acid (i.e. a decrease from ~16% to <4%) and linolenic acid (a decrease from ~35% to <10%). These changes result in oils that are superior on multiple levels: they are healthier, more oxidatively stable and better suited for production of certain commercial chemicals, including biofuels. As expected, *A. thaliana* T_2_ and T_3_ generation seeds exhibiting these types of altered fatty acid profiles were homozygous for disrupted *FAD2* alleles. In the allohexaploid, Camelina, guide RNAs were designed that simultaneously targeted all three homoeologous *FAD2* genes. This strategy that significantly enhanced oil composition in T_3_ and T_4_ generation Camelina seeds was associated with a combination of germ‐line mutations and somatic cell mutations in *FAD2* genes in each of the three Camelina subgenomes.

## Introduction

*Camelina sativa* (hereafter, Camelina) is an oil seed crop of the Brassicaceae family that has attracted considerable attention because of its short growing season, and its productivity in geographic regions with limited rainfall and soil fertility (Iskandarov *et al*., 2014; Pilgeram *et al*., 2007; Zubr, 1997). Despite the increasing commercial interest in Camelina, a limitation to the wider use of its seed oil in biofuels, lubricants and food applications is its high content of polyunsaturated fatty acids, particularly linolenic acid (18:3), which accounts for 30%–40% of seed oil from most Camelina cultivars (Iskandarov *et al*., 2014). The high polyunsaturated content makes Camelina oil more susceptible to oxidation and food products derived from this oil more prone to rancidity (Frolich and Rice, 2005). To address this deficiency in Camelina oil quality, efforts have been directed at increasing the content of the more oxidatively stable oleic acid by suppression of *FAD2* genes for the Δ12 oleic acid desaturase that converts oleic acid to linoleic acid (18:2) and linolenic acid (18:3) (Hutcheon *et al*., 2010; Kang *et al*., 2011; Nguyen *et al*., 2013). The result of this genetic modification is an increase in oleic acid content and corresponding decreases in polyunsaturated fatty acid (18:2 and 18:3) content of seed oils (Nguyen *et al*., 2013). Methods for *FAD2* suppression in Camelina and other crops have included RNA interference (RNAi; Clemente and Cahoon, 2009; Graef *et al*., 2009; Jung *et al*., 2011; Nguyen *et al*., 2013), microRNAs (Belide *et al*., 2012), TALENs (Haun *et al*., 2014) and standard mutagenesis and selection (e.g. Kang *et al*., 2011; Pham *et al*., 2012; Thambugala *et al*., 2013; Wells *et al*., 2014).

The recent advent of the highly efficient and facile CRISPR/Cas9 system for gene editing (Cong *et al*., 2013; Jinek *et al*., 2012; Mali *et al*., 2013) in animals (Petersen and Niemann, 2015; Proudfoot *et al*., 2015) and plants (Weeks *et al*., 2015) offers the opportunity to determine whether the oil composition of Camelina seeds could be favourably altered by knocking out the activities of a few or all of the six fatty acid desaturase 2 (*FAD2*) genes present in the genome of this allohexaploid plant (Hutcheon *et al*., 2010; Kang *et al*., 2011). If successful, this strategy would increase oleic acid content and lower the content of linoleic acid, linolenic acid and other long‐chain polyunsaturated fatty acids. Because the genomes of Arabidopsis and Camelina—and the *FAD2* genes, in particular (Hutcheon *et al*., 2010; Kang *et al*., 2011; Nguyen *et al*., 2013)—share strong homology with each other, we designed sgRNA constructs for use in Camelina but tested them first in Arabidopsis to determine their efficacy in creating Cas9/sgRNA‐mediated gene mutations and changing the fatty acid composition of seeds. This strategy assumed that Arabidopsis plants homozygous for *FAD2* gene mutations could be obtained within two or three generations, whereas three or more generations would be required to inactivate most or all of the *FAD2* genes in allohexaploid Camelina. The nuclear genome of Camelina, as well as that of other allohexaploids such as bread wheat, contains three separate subgenomes that, because they avoid intergenomic (homoeologous) recombination, behave as three distinct and separate diploid genomes (Comai, 2005; Feldman and Levy, 2012; Madlung and Wendel, 2013). While homoeologous recombination can be manipulated genetically in certain species, there is effectively no opportunity to replace a functional *FAD2* gene with a defective allele from another subgenome through classical breeding techniques. In other words, homozygous or biallelic knockouts of *FAD2* genes must be achieved independently for all three subgenomes if complete depletion of FAD2 enzyme activity is to be achieved.

In this report, we present evidence for efficient, multigenerational, knockout of *FAD2* genes by the Cas9/sgRNA gene editing system in somatic and germ‐line cells of Arabidopsis and Camelina leaves and seeds. We demonstrate in these initial experiments that such alterations lead to increases in oleic acid composition from ~16% to >50% and total monounsaturated fatty acid (18:1, 20:1, 22:1) from ~32% to >70% with concurrent decreases in linoleic and linolenic fatty acid content.

## Results and discussion

### FAD2 as a target for Cas9/sgRNA modification

The results presented here are the first report from a long‐term project to significantly change the oil composition of Camelina seeds using the Cas9/sgRNA gene editing system to knockout the activity of *FAD2*, a key gene involved in the synthesis of polyunsaturated fatty acids. All three pairs of *FAD2* genes in the allohexaploid, *C. sativa,* have all been shown to be active in developing seeds (Hutcheon *et al*., 2010; Kang *et al*., 2011; Nguyen *et al*., 2013). Thus, knocking out one or more of these genes should decrease the conversion of oleic acid to linoleic acid and linolenic acid (Figure S1). Because Camelina *FAD2* genes share extremely high homology with their counterparts in the much easier and quicker to manipulate diploid plant, *Arabidopsis thaliana* (hereafter Arabidopsis) (Hutcheon *et al*., 2010; Kang *et al*., 2011; Nguyen *et al*., 2013), our strategy (discussed in detail below) has been to design Cas9 and sgRNA genes that often, but not always, target the same 20‐nucleotide (nt) DNA sequence [23 nt, if the NGG protospacer adjacent motif (PAM) region is included] present in both Arabidopsis and Camelina genes. As detailed below, seed fatty acid profiles in T_2_, T_3_ and T_4_ generations of transgenic Camelina were followed along with DNA sequence modifications in both leaf tissues and seeds for each generation.

**Figure 1:**
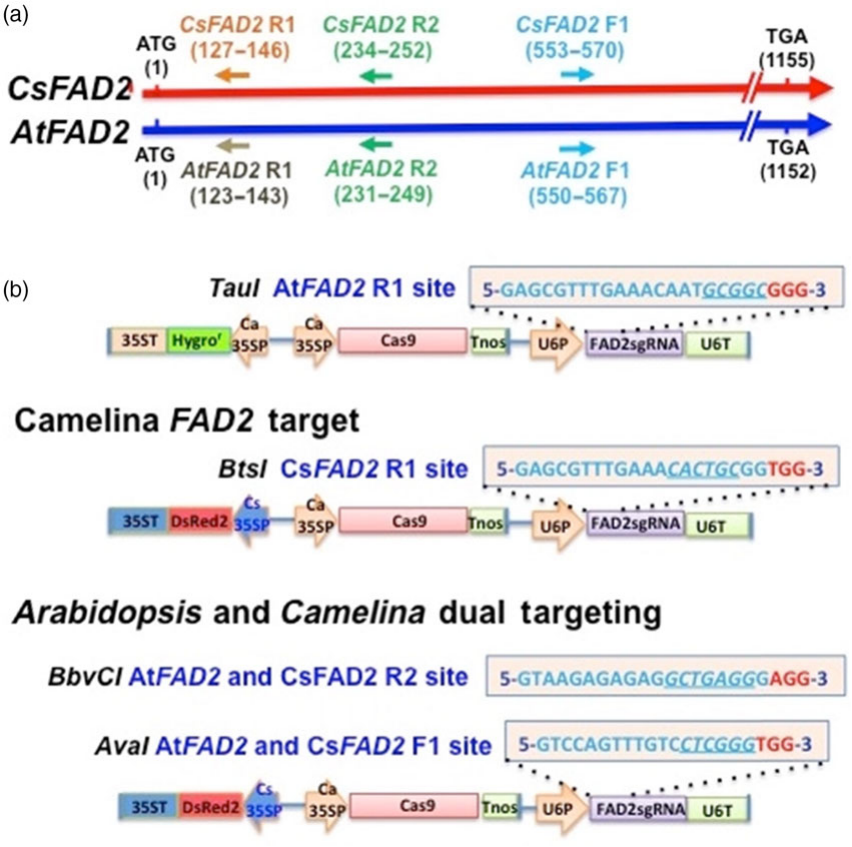
Targeting of the Camelina and Arabidopsis *FAD2* genes for knockout by the Cas9/sgRNA gene editing system. (a) Cas9/sgRNA targeting sites in the *FAD2* genes of Camelina (top line) and Arabidopsis (bottom line). ATG, the *FAD2* gene initiation codon starting at nucleotide 1; TGA, the *FAD2* gene termination codon ending at nucleotide 1155 (Camelina) or 1152 (Arabidopsis). (b) Cas9/sgRNA gene constructs for targeting the *FAD2* genes of Camelina and Arabidopsis. Boxed and lettered in blue are 20‐nucleotide target sequences (plus 3‐nucleotide PAM sequence—in red) contained in sgRNA genes for the Arabidopsis *FAD2*R1 target site (containing a *Tau*I restriction enzyme cut site at the predicted point of Cas9/sgRNA‐mediated DNA cleavage), the Camelina RI site (with a *Bts*I site), Arabidopsis and Camelina R2 sites (with a *Bbv*CI site) and Arabidopsis and Camelina F1 sites (with an *Ava*I site). Transcription of the sgRNA gene is controlled by the Arabidopsis U6 gene promoter (U6P) and termination (U6T) regions, the Cas9 gene by Cauliflower mosaic virus (Ca) *35S* promoter and termination regions and the DsRed2 red fluorescence gene by cassava mosaic virus (Cs) *35S* promoter and termination regions.

For both Arabidopsis and Camelina, three 20‐nt target sites for Cas9/sgRNA‐mediated DNA cleavage of the *FAD2* gene were selected. These 20‐nt target sequences were oriented within each gene either in the same 5′ to 3′ direction as the reading frame of the gene (the ‘forward’ direction) or in the 5′ to 3′ direction on the opposite (reverse) strand of DNA. The location of each of these sites in the *FAD2* genes of Camelina and Arabidopsis is shown in Figure 1a. Each of these sites was deliberately chosen to be located in the 5′ portion of each gene to ensure that gene disruptions altering the gene's reading frame would produce a translation product lacking enzymatic activity. The sites were also chosen to contain a restriction enzyme cut site at the site of Cas9/sgRNA‐directed DNA cleavage three base pairs upstream of the PAM site. As described below, if Cas9/sgRNA activity results in DNA cleavage at this site and if the double‐stranded DNA break (DSB) is repaired by the error‐prone nonhomologous end‐joining (NHEJ) DNA repair mechanism, the restriction enzyme site often will be destroyed. Thus, if the region containing this site is amplified using the appropriate PCR primers, amplicons containing nonmodified restriction enzyme sites will be cleaved, whereas amplicons containing a destroyed restriction site will remain intact. Size analysis of the restriction enzyme‐digested amplicons thus allows a simple qualitative and rapid means for detecting leaf or seed samples that contain (or do not contain) genes whose DNA sequence has been altered by the Cas9/sgRNA gene editing complex.

Drawings depicting the structures of the Cas9 gene and sgRNA gene constructs designed to recognize and cleave each of the three *FAD2* gene target sites (Figure 1a) are provided in Figure 1b. The complete DNA sequence for each construct is provided in Supporting Information and Experimental procedures. In each construct, the Cas9 gene is driven by the cauliflower mosaic virus *35S* gene promoter and terminated with the *Agrobacterium tumefaciens Tnos* gene termination sequence as described previously (Jiang *et al*., 2013, 2014). The sgRNA genes are driven by the Arabidopsis U6 promoter and followed by the termination region from the same gene. The construct targeting the Arabidopsis *FAD2*R1 site contained a hygromycin resistance gene to allow for rapid selection of transgenic seeds (Figure 1b). The three other constructs each contained a DsRed2 gene that allowed the use of a hand‐held green fluorescent flashlight to easily detect and select red fluorescing transgenic T_1_ seeds (Lu and Kang, 2008; Nguyen *et al*., 2013) obtained from T_0_ plants produced by transformation of Camelina or Arabidopsis using the floral dip transformation technique. Importantly, as part of our strategy, only red fluorescent seeds were selected for further evaluation at each generation. Thus, presuming normal Mendelian inheritance of the T‐DNA region containing the DsRed gene and the accompanying Cas9 and sgRNA genes, each plant analysed in this study had the potential to actively express the Cas9/sgRNA complex in most or all of its tissues. Important implications of this strategy are discussed below.

### Extensive Cas9/sgRNA‐mediated gene editing in leaf and seed tissues

Over 200 T_1_ Arabidopsis plants and 300 T_1_ Camelina plants that carried various Cas9 and sgRNA genes targeting *FAD2* genes were generated and analysed during this study (Data Set S1). From these T_1_ plants, we produced large numbers of T_2_ and T_3_ plant progeny and seeds (Data Set S1) and limited samples of T_4_ seeds. Each plant generated in this study was given a unique name (e.g. AtFAD2R1 T1‐10‐8‐7 is the 7th T_3_ progeny plant from the 8th T_2_ progeny plant from the 10th T_1_ Arabidopsis parent plant targeted for gene disruption at the R1 site of the *FAD2* gene).

To speed the selection of plants carrying mutations in a targeted gene and to discard plants lacking Cas9/sgRNA‐directed gene mutations, DNA was extracted from leaf tissue of transgenic T_1_ plants and used for PCR amplification of sgRNA target sites of interest using sets of primers listed in Table S1. As described above, each selected target site contained a restriction enzyme recognition sequence overlapping the expected Cas9/sgRNA cleavage site three base pairs upstream of the NGG PAM site, for easy detection of mutation events. As a result, if the Cas9/sgRNA complex created a DSB at the expected site and if an error occurred during DNA repair by the NHEJ mechanism, the restriction enzyme recognition site would be destroyed. In such cases, subjecting isolated DNA to digestion with the restriction enzyme appropriate to match the target site prior to PCR amplification of the target region would produce a nondigested, full‐length, PCR product. Conversely, if no Cas9/sgRNA‐directed mutation occurred at the site, the isolated DNA would be cleaved by the restriction enzyme and no PCR product would be visible on a standard ethidium bromide‐stained agarose gel. An example of such a PCR/restriction enzyme (PCR/RE) analysis is shown in Figure S2 in which DNA from four different transgenic Arabidopsis plants displayed full‐length, 322‐bp PCR products, while DNA from a nontransgenic control plant produced the expected 244‐ and 78‐bp restriction enzyme digestion fragments. Several DNA samples testing positive in the PCR/RE analyses were subjected to Sanger DNA sequencing of PCR‐amplified target sites to determine the exact nature of the mutation. In most experiments, a few DNA samples tested negative in the PCR/RE analyses were also sequenced as controls and to confirm that they, indeed, were true negatives.

The expected ability of the Cas9/sgRNA system to cause gene mutations in the *FAD2* genes of Arabidopsis and Camelina plants was confirmed by DNA sequencing. Sequence analyses of DNA from leaf and seed samples (Data Set S2) confirmed multiple mutations over multiple generations at each of the three target sites in each of the three different *FAD2* gene types present, respectively, in the A, B and C subgenomes of the allohexaploid genome. In the overview presented in Data Set S2, DNA sequences obtained by Sanger sequencing are provided for 328 *FAD2* gene target sites, 258 of which contained the kinds of short‐length nucleotide insertions and deletions typical of NHEJ DNA repair that follows creation of DSBs by the Cas9/sgRNA complex. Interestingly, over half of these mutations were the result of a single‐nucleotide insertion that was nearly always located at the predicted Cas9/sgRNA cut site 3 bp upstream of the PAM site. Among these single‐nucleotide insertions, there was a marked preference for T (50%) and A (33%) nucleotide inserts over G (12%) and C (6%) nucleotide inserts. The summary at the bottom of Data Set S2 shows that approximately 19% of mutations involved nucleotide deletions over 3 bp in size and approximately 5% involved nucleotide insertions of 3 bp or more. Because all single‐nucleotide insertions and most other insertions and deletions change the reading frame of the gene and because all target sites were deliberately chosen to be located in the 5′ region of the gene (Figure 1a), the vast majority of mutations created in the *FAD2* genes are predicted to lead to gene knockout. Because of single‐nucleotide polymorphisms (SNPs) between the three types of *FAD2* genes found in the A, B and C subgenomes of Camelina (Hutcheon *et al*., 2010), we were able to determine that all of the *FAD2* genes in each of the subgenomes were efficiently targeted by the Cas9/sgRNA complex.

To assess whether off‐target mutations occurred, three sites in the genome most similar to the target *FAD2* site were PCR‐amplified and sequenced by Sanger chemistry using *C. sativa* DNA from *C. sativa* plants with a high mutation rate at the *FAD2*R1 target site. These three best off‐target candidate loci contain a PAM and complete homology to the 10‐bp ‘seed region’ that is at the 3′ end of the *FAD2* protospacer (Jiang *et al*., 2015). In the 5′ ends, they contain five or six mismatches to the *FAD2* protospacer (Figure S6). The resulting DNA sequence chromatograms from the Cas9‐positive plants were indistinguishable from the WT chromatograms, suggesting that off‐target mutations are not prevalent when using the *FAD2* protospacer of interest (Figure S6).

Examination of the data in Data Set S2 also revealed that approximately 8% of the DNA sequencing reads contained a combination of single‐nucleotide polymorphisms found in two (or, in one case, three) of the homoeologous *FAD2* genes. Such combinations could be due to unexpected recombination of homoeologous chromosome fragments following Cas9/sgRNA‐generated chromosome breaks or, alternatively, to generation of incomplete DNA transcripts during polymerase chain reaction (PCR) DNA amplification and the subsequent utilization of these incomplete strands as templates during ensuing rounds of amplification. To distinguish between these two possibilities, the same DNA samples used for PCR amplification and Sanger DNA sequencing shown above were subjected to PCR amplification and DNA sequencing using Illumina Amplicon‐Seq techniques in which thousands of DNA strands were sequenced from DNA isolated from each plant under study.

### PCR artefacts are generated during amplification of the three highly homologous copies of the *FAD2* gene in Camelina

**Figure 2:**
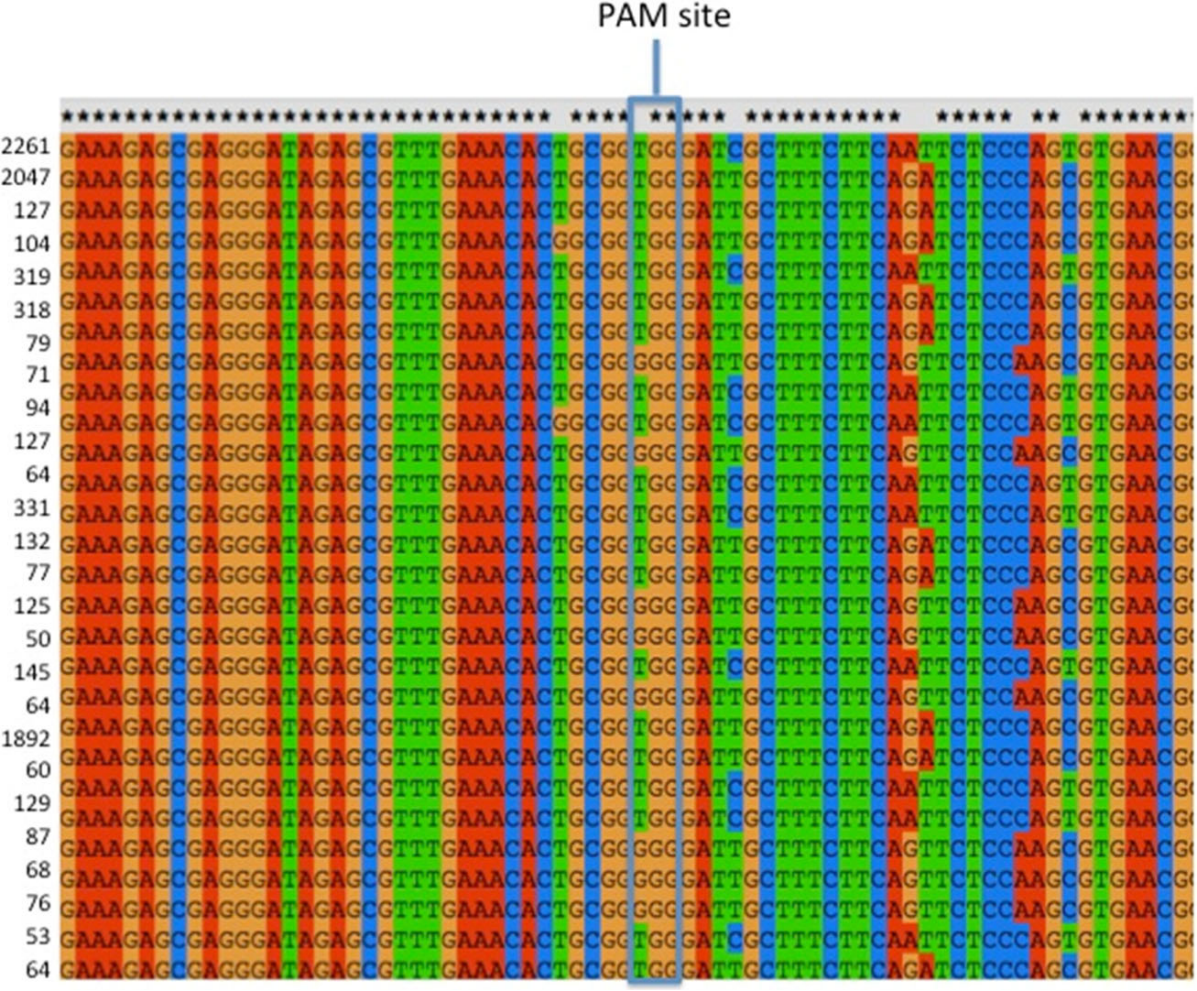
Clustal alignment of the sequences of the Cs*FAD2*R1 target site in *Camelina sativa* leaf wild‐type sample. Illumina Amplicon‐Seq was used for DNA sequencing. Read frequency counts for each sample are provided to the left of the sequences. Only the sequences found at least 50 times are depicted. The three most common sequences represent the three WT homoeologs sequences. Asterisks denote nucleotides conserved in all three subgenome homoeologs (A–C). Lack of an asterisk denotes a nucleotide position at which there is a single‐nucleotide polymorphism in one of the three homoeologs.

**Figure 3:**
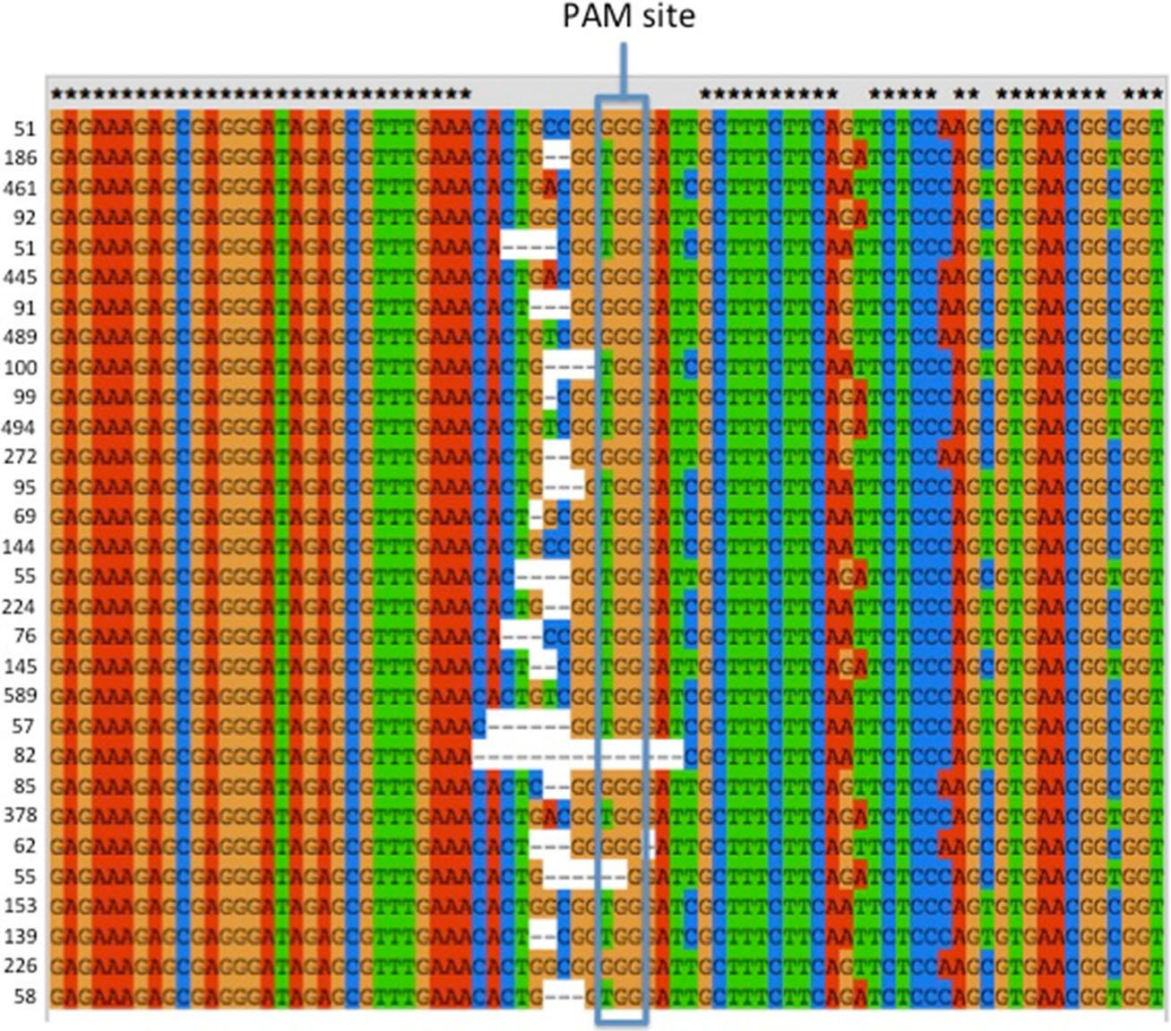
Clustal alignment of the sequences of the Cs*FAD2*R1 target site in *Camelina sativa* leaf sample T1‐1‐3 exhibiting Cas9/sgRNA‐mediated gene editing. Illumina Amplicon‐Seq was used for DNA sequencing. Read frequency counts for each sample are provided to the left of the sequences. Only the most common sequences found at least 50 times are depicted. Asterisks denote nucleotides conserved in all three subgenome homoeologs (A, B and C). Lack of an asterisk denotes a nucleotide position at which there is a single‐nucleotide polymorphism in one of the three homoeologs.

The allohexaploid Camelina contains three distinct yet closely related homoelogous subgenomes, called A, B and C. Two subgenomes consist of seven chromosomes and the third of six chromosomes adding to the somatic chromosome content of 20 pairs (Hutcheon *et al*., 2010). Individual single‐copy genes, such as the *FAD2* gene, thus come in three allelic pairs that can be distinguished based on single‐nucleotide polymorphisms (SNPs) specific to each subgenome (Hutcheon *et al*., 2010; Kang *et al*., 2011; Nguyen *et al*., 2013) (Data Set S2 and Figures 2 and 3). Our Sanger sequence data (Data Set S2) indicated that approximately 8% of the DNA sequencing reads contained a combination of single‐nucleotide polymorphisms found in two (or, in one case, three) of the homoeologous *FAD2* genes. Specifically, of the 259 Cas9/sgRNA‐mediated mutations observed in our Sanger sequencing of cloned FAD2 gene DNA, 21 contained apparent chimeras between homoeologous chromosomes (Data Set S2). All possible combinations of homoeologous chromosome exchanges (A + C, B + C, etc.) were observed. No chimeric sequences were observed in the limited number of DNA sequences (i.e. 30) obtained from Sanger DNA sequencing of wild‐type Camelina DNA samples.

Because all homoeologs were targeted by the same guide RNA, it is possible that these sequences originated from recombination between homoeologous chromosomes following Cas9/sgRNA‐generated chromosome breaks. Alternatively, as all sequences were amplified using the same primers, these sequences could have originated from chimeric PCR amplicons. To distinguish between these two possibilities, 30 transgenic plants expressing the Cas9 and sgRNA and two wild‐type plants were selected from the samples analysed above and subjected to PCR amplification and DNA sequencing using Illumina Amplicon‐Seq, generating thousands of sequences from each sample (Data Set S3 and S4). Analysed sequences were 350 bp long and amplified using nonhomoeologous specific primers, that is primers that perfectly matched all three homoeologous sequences. Paired‐ended reads were obtained and processed using custom scripts (see Experimental procedures for details) to assign sequences to specific homoeologs, based on the SNPs distinguishing the three WT sequences. Chimeric sequences were observed in both transgenic and wild‐type data sets (Data Set S3), and the percentage of chimeric sequence was at least as high in the control as in the transgenic samples (Table S2—summarized in Figure S3). These results thus provide no evidence for recombination between homoeologous DNA strands following Cas9/sgRNA‐generated cleavage of the DNAs, but, rather, point to artificial recombination between homoeologous *FAD2* gene strands during PCR amplification as the source of the observed chimeras. Reports in the recent literature (e.g. Liu *et al*., 2014 and references therein) document that PCR amplification of DNA from distinctly different, but highly homologous, DNA molecules can lead to the generation of ‘recombinant’ DNA strands due to a small number of incomplete PCR amplicons being used as primer sequences. These studies coupled with the present study point to the need for extreme care in interpreting data from experiments using PCR amplification of DNA from polyploid species.

### CRISPR/Cas9 action results in a variety of mutant alleles

**Figure 4:**
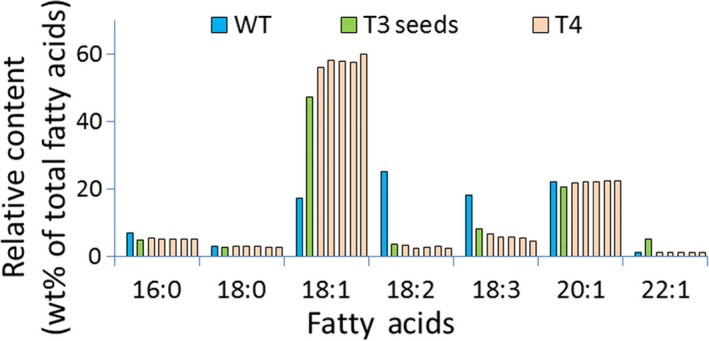
Seed oil profile of seeds from Arabidopsis plants transformed using Cas9/sgRNA targeting the R2 site of the AtFAD2 gene. Blue: wild‐type control; green: T3 seeds (from T2 plant T1‐25‐4); light red: T4 seeds (from T3 plant, in order, T1‐25‐4‐1, T1‐25‐4‐2, T1‐25‐4‐3, T1‐25‐4‐4, T1‐25‐4‐5) derived from T1‐25‐4. Methods for extraction and measurement of Arabidopsis seed fatty acids are detailed in Experimental procedures.

Sequencing the targeted sites allowed for an in‐depth analysis of the patterns of mutations as, in total, 329 Sanger sequences and 410 490 Illumina sequences were analysed. Analyses of the initial Sanger data and the Illumina Amplicon‐Seq data provided useful comparisons. First, the Amplicon‐Seq data confirmed the presence of many different types of insertions and deletions at the CS*FAD2*R1 target site (average of 71% indels) and the lack of insertions and deletions at the same site in DNA samples obtained from wild‐type plant tissues (average 0.6% indels) (Figures 3 and 4 and Data Set S3). Next, it confirmed that all three homoeologs were efficiently targeted although the percentage of mutant sequence was higher in homoeologs B sequences (84.4%) than in homoeologs A (60.7%) or C (67.3%). Averaged across all mutated samples and homoeologs, Sanger reads indicated 21.2% WT, 78.2% indel‐containing and 0.6% SNP‐containing sequencing. These numbers were similar to those obtained from the Illumina reads, which indicated 27.5%, 71.1% and 1.4% of WT, indel‐containing and SNP‐containing reads, respectively. There were nearly twice as many Illumina reads containing insertions as compared to deletions. Within the Illumina reads, >99% of the insertions were single‐nucleotide insertions with the following distribution: 49.1% T, 35.3% A, 12.9% G and 2.8% C. This distribution was consistent with observation from the Sanger data (50% T, 33% A, 12% G and 6% C), demonstrating strong insertion preferences. Deletions were more variable in both data sets. Of the 64 368 Illumina reads that contained deletions, half were 1‐bp deletions, a quarter were 2‐bp deletions, 11% were 3‐bp deletions, and the remaining 12% were >3‐bp insertions. For WT1, the Illumina reads were 92.2% WT, 0% indel‐containing and 7.1% SNP‐containing, and for WT2, they were 91.9%, 1.3% and 6.8%, respectively. The SNPs and indels observed in WT1 and WT2 are assumed to originate primarily from PCR‐derived artefacts documented above and provide a base estimate of false positives.

### Significant Cas9/sgRNA‐mediated changes in seed oil composition

Analyses of fatty acid composition of seeds from both Arabidopsis and Camelina plants containing Cas9/sgRNA‐mediated mutations in their *FAD2* genes as well as from control plants lacking such modifications demonstrated a range of oleic acid content from the usual ~16% to greater than 50% (Data Set S5). One of the best‐performing Arabidopsis lines (FAD2R2 T1‐25‐4 and its T_4_ seed progeny) showed an oleic oil concentration of ~50% in the T_3_ generation. This increased to a range between ~55% and ~60% in the T_4_ generation (Figure 4). As expected from results of earlier studies with Cas9/sgRNA‐disrupted genes of Arabidopsis (Feng *et al*., 2014; Jiang *et al*., 2014), DNA sequencing of the FAD2 genes in T_2_ and T_3_ plants producing these high oleic acid seeds showed them to be homozygous for disruptions of the *FAD2* genes (Figure S4). Also, as expected, the number of generations needed for oleic acid seed content to increase in the allohexaploid, Camelina, was more than in diploid Arabidopsis—although a few T_4_ Camelina seeds contained oleic acid levels of 50% or greater (Data Set S5 and Figure S5a). In each case in which there was a rise in oleic acid content, there was the expected proportional decrease in linoleic and linolenic acid content, leading to seeds with fatty acid composition more favourable for production of certain specialty oils and biodiesel. Targeting each of the three selected sites within the *FAD2* gene (F1, R1 and R2) for Cas9/sgRNA attack was effective in causing significant increases in production of oleic acid and decreases in linoleic acid and linolenic acid (Figure S5a) in the best‐performing Camelina lines. Production of total monounsaturated fatty acids (18:1, 20:1, 22:1) increased from ~30% in wild‐type seeds to as high as ~74% (Figure S5b). These results demonstrate that, as with the use of RNAi, gene silencing and other gene editing technologies, Cas9/sgRNA system can be used to cause significant and favourable changes in seed fatty acid composition in both Arabidopsis and the emerging oil seed crop, Camelina. Consistent with our molecular off‐target analysis, no evidence of overt off‐target effects was observed in the phenotypes of plants or seeds containing Cas9/sgRNA‐mediated gene mutations.

### Changes in seed oil composition originate from the combined contributions of Cas9/sgRNA‐mediated mutations in somatic cells of Camelina seeds and germ‐line mutations

**Figure 5:**
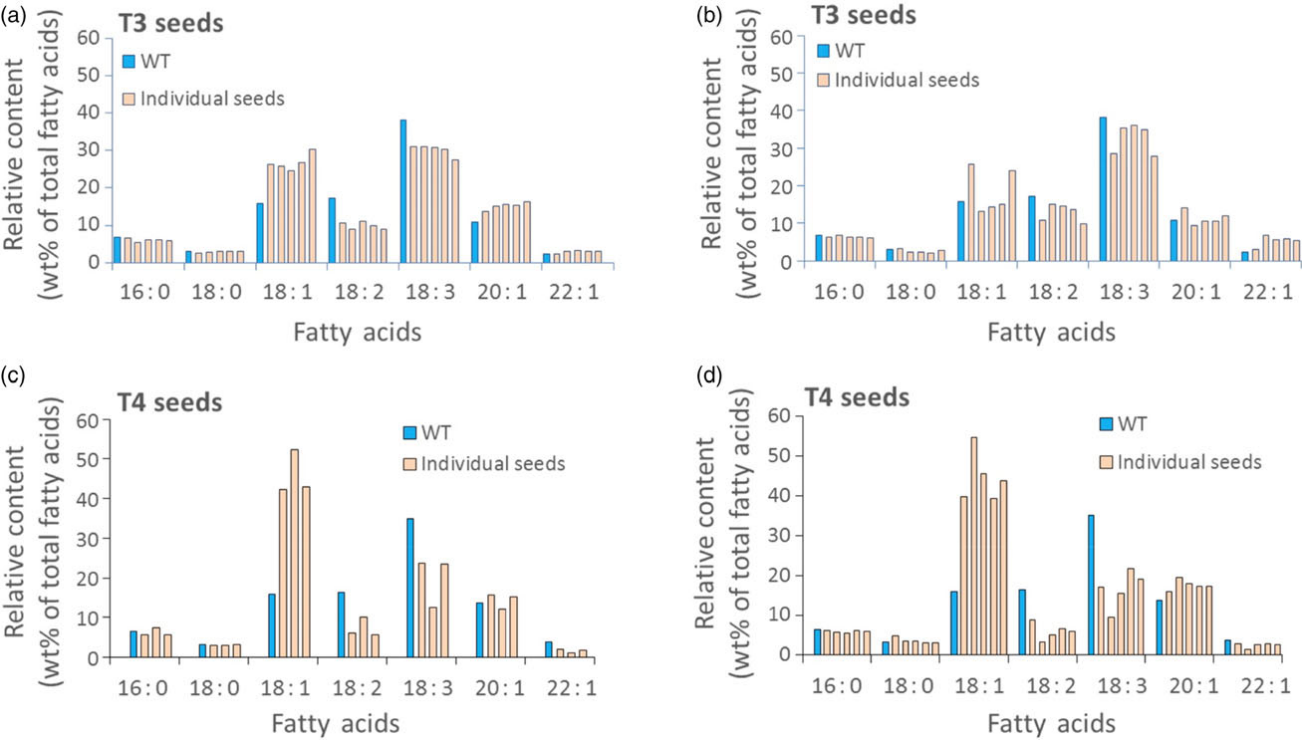
Oil profile in seeds of Camelina plants transformed using Cas9/sgRNA targeting the R1 site of the *FAD2* genes. Variation of T3 seed oil profiles of individual seeds from T_2_ plant T1‐1‐3 (a) and T_2_ plant T1‐5‐1 (b); variation of seed oil profiles of individual T_4_ seeds from T_3_ plant T1‐3‐2‐3 (c) and T_3_ plant T1‐5‐1‐1 (d). Blue: wild‐type control; light red: individual seed tested. Methods for extraction and measurement of Camelina seed fatty acids are detailed in Experimental procedures.

While expression of the Cas9 and sgRNA genes is clearly responsible for the changes in seed oil composition documented in Data Set S3, the data presented in Data Set S2 and S4 raise questions with regard to the source of this phenotype and the probability of its stable inheritance—at least in the present set of Camelina plants that contain and appear to maintain an active set of Cas9 and sgRNA genes. Careful analysis of the DNA sequences in the mutant genes shown in Data Set S2–S4 suggest, as detailed below, that while some mutant *FAD2* genes observed in the present study may be inherited from one generation to the next, the large number of different mutations observed in DNA from a single sample of leaves or seeds from an individual plant points to a contribution of somatic cell mutations to the pool of DNA sequence variants observed. Specifically, cataloguing all types of mutations observed from the Amplicon‐Seq data resulted in, on average, 59 different mutation types per sample, evenly distributed between the three homoeologous sequences (Data Set S4). We have also noted that the proportion of indel sequences was consistently higher in leaf samples (71.6%) compared to seed samples (58.5%), suggesting CRISPR/Cas9 activity is higher in somatic tissues than in germinal tissues (Data Set S3 and summarized in Figure S7). This is in agreement with our earlier observations and conclusions regarding Cas9/sgRNA‐derived genetic mosaicism in somatic cells (Jiang *et al*., 2014) and similar conclusions by others (e.g. Feng *et al*., 2014). Thus, the changes in fatty acid composition observed in individual seeds (Data Set S5) could be due to the effects of germ‐line mutations combined with *FAD2* mutations occurring in somatic cells during seed development. The latter would give rise to a mosaic of overlapping patches of somatic cells with varying combinations of mutations in the six copies of the *FAD2* genes. This conclusion is further strengthened by our observations that when fatty acid composition is determined for individual T_3_ seeds from a single T_2_ plant or individual T_4_ seeds from a single T_3_ plant (Figures 4, 5 and Data Set S5), there can be noticeable variability in oleic acid levels from seed to seed (e.g. 39%, 40%, 43%, 45% and 55% for seeds from plant T1‐5‐1‐1)—a potential reflection of a mosaic of somatic cells in individual seeds that carry variable numbers of inactivated *FAD2* genes.

Germ‐line mutations may be detected by the Mendelian inheritance of unique mutations in Cas9‐positive plants or by the inheritance of Cas9‐induced mutation in Cas9‐negative plants. In the Amplicon‐Seq data set from leaves of 27 individual Cas9‐positive *C. sativa* plants, there are many cases in which a unique Cas9‐induced mutation occurs in over 25% of the reads from a homologous pair (Data Set S4), suggesting that the mutation may have been inherited from one of the parents. On the other hand, many of these mutations are of a very common type and the high percentage could result from multiple somatic mutations as well. To better quantify the presence of germ‐line mutations, we analysed Cas9‐negative plants that were derived from a Cas9‐positive *C. sativa* plant, T1‐5 (Figure S8). Lack of the Cas9 gene in 20 nonfluorescent (i.e. ‘black’) seed progeny in each of three different T1‐5 lines (i.e. T1‐5‐1‐7B, T1‐5‐1‐8B, T1‐5‐1‐9B) was verified by PCR analyses (Figure S9). Sanger DNA sequence chromatograms indicate that some progeny from T1‐5‐1‐8B contain a mutation in the B homoeolog and some progeny from T1‐5‐1‐9B contain a mutation in the A homoeolog (Figure S10b). No mutation was detected in the other pools or in the C homoeolog (Figure S10c). From the pools that contained mutations, individual plants were sequenced and could easily be classified as ‘WT’, ‘homozygous A insertion’ or ‘heterozygous’ (Figure S10a). From these allelic ratios, we inferred that two of the 18 FAD2 homoeologous copies, or 11.1%, carried a mutation in the three Cas9‐negative parents. Additionally, as demonstrated by this germ‐line inheritance, we have generated Cas9‐negative *C*. *sativa* plants that contain homozygous FAD2 knockouts in homologous pairs (Figure S10b).

While the presence of *FAD2* gene knockouts in the germ‐line may explain a substantial portion of the increase in oleic acid content observed, the high abundance of somatic cell mutations in developing seeds [documented by the large number of different Cas9/sgRNA‐generated mutations in every line tested (Data Set S4)] suggests that *FAD2* knockout mutations in somatic cells also may contribute to increases in oleic acid concentrations.

Cas9/sgRNA activity may contribute to changes in oil composition via mechanisms other than reduced expression. This is consistent with previous observations that *FAD2* functions as a homodimeric enzyme (Lou *et al*., 2014) and that expression of nonfunctional mutants of *FAD2* in cotton (Chapman *et al*., 2001, 2008) or closely related desaturases, epoxygenases, and hydroxylases in a variety of other plant species (Broun and Somerville, 1997; Broun *et al*., 1998; Cahoon *et al*., 1999; Singh *et al*., 2001) can cause inhibition of *FAD2* desaturase activity—presumably through formation of nonfunctional heterodimers (i.e. ‘subunit poisoning’). In these species, such dominant negative inhibition of *FAD2* activity results in striking increases in oleic acid and decreases in linoleic and linolenic acids. (Broun and Somerville, 1997; Broun *et al*., 1998; Cahoon *et al*., 1999; Chapman *et al*., 2001, 2008; Lou *et al*., 2014; Singh *et al*., 2001). Thus, because the Cas9/sgRNA constructs used in the present studies primarily cause frame‐shift mutations in the 5′ region of the targeted *FAD2* genes, it is likely that polypeptides are produced that contain authentic N‐terminal *FAD2* domains. It is possible that such molecules can bind with functional *FAD2* monomers and partially inhibit desaturase activity. Future experimentation to test this hypothesis and, more importantly, future analyses of new generations of mutant Camelina plants containing homozygous or biallelic knockouts of *FAD2* genes will be needed to provide a better understanding of the high oleic acid phenotypes presently observed.

A potentially significant advantage of using gene editing techniques such as ZFNs, TALNs and Cas9/sgRNAs is that once the desired gene editing event(s) have been obtained, the editing genes can be eliminated by simple cross‐breeding techniques and the desirable phenotype stabilized by production of homozygous progeny (e.g. Li *et al*., 2012; reviewed in Weeks *et al*., 2015 and Petolino and Kumar, 2015). A lesson from the present study is that obtaining the desired phenotypic stability in some allohexaploid crops, such as Camelina, likely will require several generations in which the Cas9/sgRNA genes remain present and active. The rapid screening of seeds carrying active Cas9 and sgRNA genes by virtue of their easily detectable DsRed fluorescence is an attractive tool for achieving this goal. One potential future approach to gaining a better, but not complete, picture in regard to this uncertainty will be to analyse DNA from pollen samples from individual plants (or, if technically possible, from single pollen grains) to better determine the complement of wild‐type and mutant *FAD2* genes and the ratio between them. Alternatively, it is possible that germ‐line mutations will be achieved more efficiently if the editing genes are driven by a different promoter, with documented expression in meristematic tissues. For example, homozygous mutants were recently obtained in a single generation in *A. thaliana* using a variety of promoters including an egg cell‐specific promoter (Wang *et al*., 2015), the *YAO* promoter that is active in meristematic cells (Yan *et al*., 2015), the anther‐specific *DD45* gene promoter (Mao *et al*., 2016) and the *INCURVATA2* gene promoter (Hyun *et al*., 2015).

## Conclusions

Several important conclusions can be drawn from the present study. The Cas9/sgRNA system for gene editing is active in Camelina over multiple generations. Mutations in the *FAD2* genes of Camelina can result in significant increases in oleic acid content and concomitant large decreases in linoleic and linolenic acid content. Based on results with other oil seed crops, future Camelina lines emanating from this continuing long‐term project that are homozygous for inactivated *FAD2* genes likely will exhibit even larger decreases in levels of long‐chain polyunsaturated fatty acids and improved oleic acid content. Future projects aimed at producing plants that also contain Cas9/sgRNA‐mediated knockouts of the fatty acid elongase, *FAE1,* genes should lead to additional boosts in oleic acid content of Camelina seeds.

## Experimental procedures

Creation and analyses of transgenic Arabidopsis and Camelina plants and seeds: *A. thaliana* ecotype (Col‐0) and *C. sativa*, cv. Suneson, were transformed using the floral dip method with vectors containing Cas9/sgRNA constructs targeting the respective Arabidopsis and Camelina *FAD2* genes. Transgenic plants were self‐pollinated, and leaves and seeds were collected from sequential generations of plants to determine by PCR/restriction enzyme analyses and DNA sequencing if gene editing events had occurred at the gene target sites. The fatty acid compositions of seeds were determined using gas chromatography. Mutant FAD2 gene DNA sequences (somatic and germ‐line) in Camelina lines containing (Cas9‐positive) and lacking (Cas9‐negative) functional Cas9 and sgRNA genes were obtained by various combinations of Sanger DNA sequencing and Illumina Amplicon‐MiSeq DNA sequencing techniques. A search for potential off‐target gene modifications was conducted using Sanger DNA sequencing of three DNA sites most closely related to the target sites within the Camelina *FAD2* genes. Details of each of these procedures are provided in Supporting Information.

## Author contributions

WJ developed methodology and performed or guided the experimentation. WJ and DPW designed the experiments. PL performed the Amplicon‐Seq experiments. PL and IMH analysed the Amplicon‐Seq data. WJ, DPW, EBC, LC and IMH analysed and interpreted the data and wrote the manuscript. DPW, EBC and LC are principal investigators on grants supporting this research.

## Supporting information


**Figure S1** Enzymatic conversion of oleic acid to linoleic and linolenic acid by fatty acid desaturase 2, the enzyme whose gene, *FAD2*, is the target for inactivation by Cas9/sgRNA genes.
**Figure S2** PCR/Restriction Enzyme (PCR/RE) analysis of total DNA extracted from leaves of Arabidopsis T1 plants expressing Cas9/sgRNAs.
**Figure S3** Comparison of the rates of PCR recombinants during PCR amplification of Camelina *FAD2* gene sequences using non‐homoeologous specific amplification primers.
**Figure S4** Inheritance and homozygous condition of Cas9/sgRNA‐mediated mutations at the R2 *Bbv*CI target site of the *FAD2* gene in T2 and T3 progeny of two transgenic Arabidopsis lines.
**Figure S5** Seed oil profiles in the best performing individual T4 seeds of Camelina plants transformed using Cas9/sgRNA targeting the R1, R2 and F1 sites in *FAD2* genes.
**Figure S6** DNA sequencing of potential Off‐Target sites.
**Figure S7** Model of CRISPR‐Cas9 action in hexaploid Camelina.
**Figure S8** Lineage of *Camelina sativa* plants used to analyzed germline mutations.
**Figure S9** Detection of the Cas9 transgene sequence in 20 individual progeny (unlabeled lanes) of each of 3 black seeds.
**Figure S10** Detection of germline FAD2 mutations.
**Table S1** Primers upstream and downstream of the target sites in *FAD2* genes in Arabidopsis and Camelina.
**Table S2** Rates of gene sequence chimera formation during PCR amplification of *FAD2* gene DNA sequences.
**Appendix S1** DNA sequences of binary vectors used in this study.

**Data Set S1** Transgenic Arabidopsis and Camelina plants created for this study.

**Data Set S2** Overview of Sanger DNA sequencing data from Camelina FAD2 gene target sites.

**Data Set S3** Analyses of DNA sequences from wild‐type Camelina plants and plants expressing CRISPR/Cas9.

**Data Set S4** Abundance of various mutations in wild‐type Camelina plants and transgenic Camelina plants expressing CRISPR/Cas9.

**Data Set S5** Fatty acid composition of seeds from wild‐type Arabidopsis and Camelina and from transgenic plants expressing CRISPR/Cas9.
