# Supplementary material for: Significant enhancement of fatty acid composition in seeds of the allohexaploid, Camelina sativa, using CRISPR/Cas9 gene editing
Source: Plant Biotechnol J. 2017 Jan 12;15(5):648–57. doi: 10.1111/pbi.12663 (PMC5399004; doi:10.1111/pbi.12663)
Supplement: Supplementary file 1 — Figure S1 Enzymatic conversion of oleic acid to linoleic and linolenic acid by fatty acid desaturase 2, the enzyme whose gene, FAD2, is the target for inactivation by Cas9/sgRNA genes. Figure S2 PCR/Restriction Enzyme (PCR/RE) analysis of total DNA extracted from leaves of Arabidopsis T1 plants expressing Cas9/sgRNAs. Figure S3 Comparison of the rates of PCR recombinants during PCR amplification of Camelina FAD2 gene sequences using non‐homoeologous specific amplification primers. Figure S4 Inheritance and homozygous condition of Cas9/sgRNA‐mediated mutations at the R2 BbvCI target site of the FAD2 gene in T2 and T3 progeny of two transgenic Arabidopsis lines. Figure S5 Seed oil profiles in the best performing individual T4 seeds of Camelina plants transformed using Cas9/sgRNA targeting the R1, R2 and F1 sites in FAD2 genes. Figure S6 DNA sequencing of potential Off‐Target sites. Figure S7 Model of CRISPR‐Cas9 action in hexaploid Camelina. Figure S8 Lineage of Camelina sativa plants used to analyzed germline mutations. Figure S9 Detection of the Cas9 transgene sequence in 20 individual progeny (unlabeled lanes) of each of 3 black seeds. Figure S10 Detection of germline FAD2 mutations. Table S1 Primers upstream and downstream of the target sites in FAD2 genes in Arabidopsis and Camelina. Table S2 Rates of gene sequence chimera formation during PCR amplification of FAD2 gene DNA sequences. Appendix S1 DNA sequences of binary vectors used in this study. [file PBI-15-648-s005.docx]

**Significant enhancement of fatty acid composition in seeds of the allohexaploid, *Camelina sativa*, using CRISPR/Cas9 gene editing**

Wen Zhi Jiang, Isabelle M. Henry, Peter G. Lynagh, Luca Comai, Edgar B. Cahoon, and Donald P. Weeks

**Supporting Information**

**Supporting Information Figures**

**
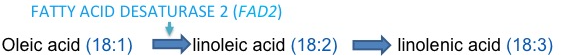
**

**SI Figure S1.** **Enzymatic conversion of oleic acid to linoleic and linolenic acid by fatty acid desaturase 2, the enzyme whose gene, *FAD2*, is the target for inactivation by Cas9/sgRNA genes.**


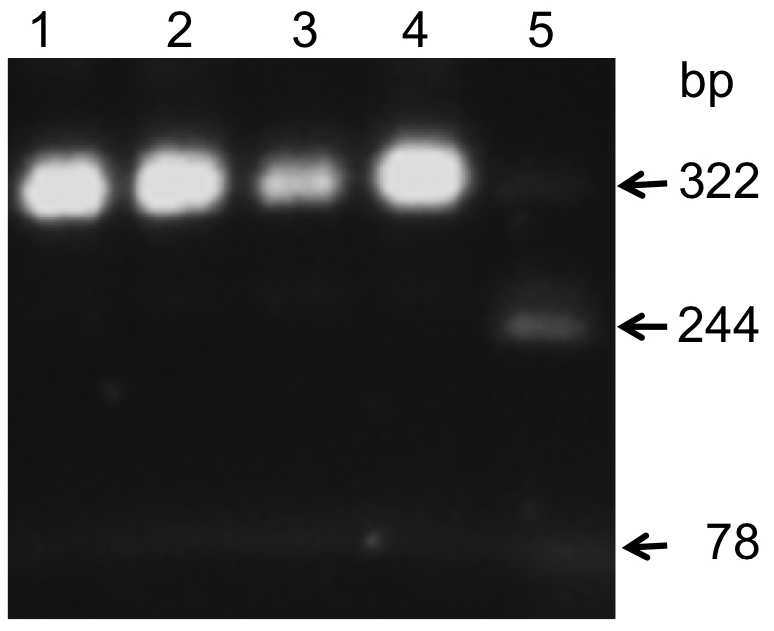


**SI Figure S2.** **PCR/Restriction Enzyme (PCR/RE) analysis of total DNA extracted from leaves of four Arabidopsis T1 plants expressing Cas9/sgRNAs** (targeting the FAD2 R2 site) demonstrating Cas9/sgRNA-mediated FAD2 gene mutagenesis. Bottom arrows indicate the expected ~244bp and 78bp DNA fragments resulting from *BbvCI* cleavage of the ~322 bp PCR product amplified from a FAD2 gene R2 site containing a nonmodified *BbvCI* restriction site. Lanes 1 to 4: DNA extracted from four different T1 transgenic plants and digested with *BbvCI* both prior and after PCR amplification, Lane 5: DNA extracted from a nontransgenic WT control plant and digested with *BbvCI* after PCR amplification.


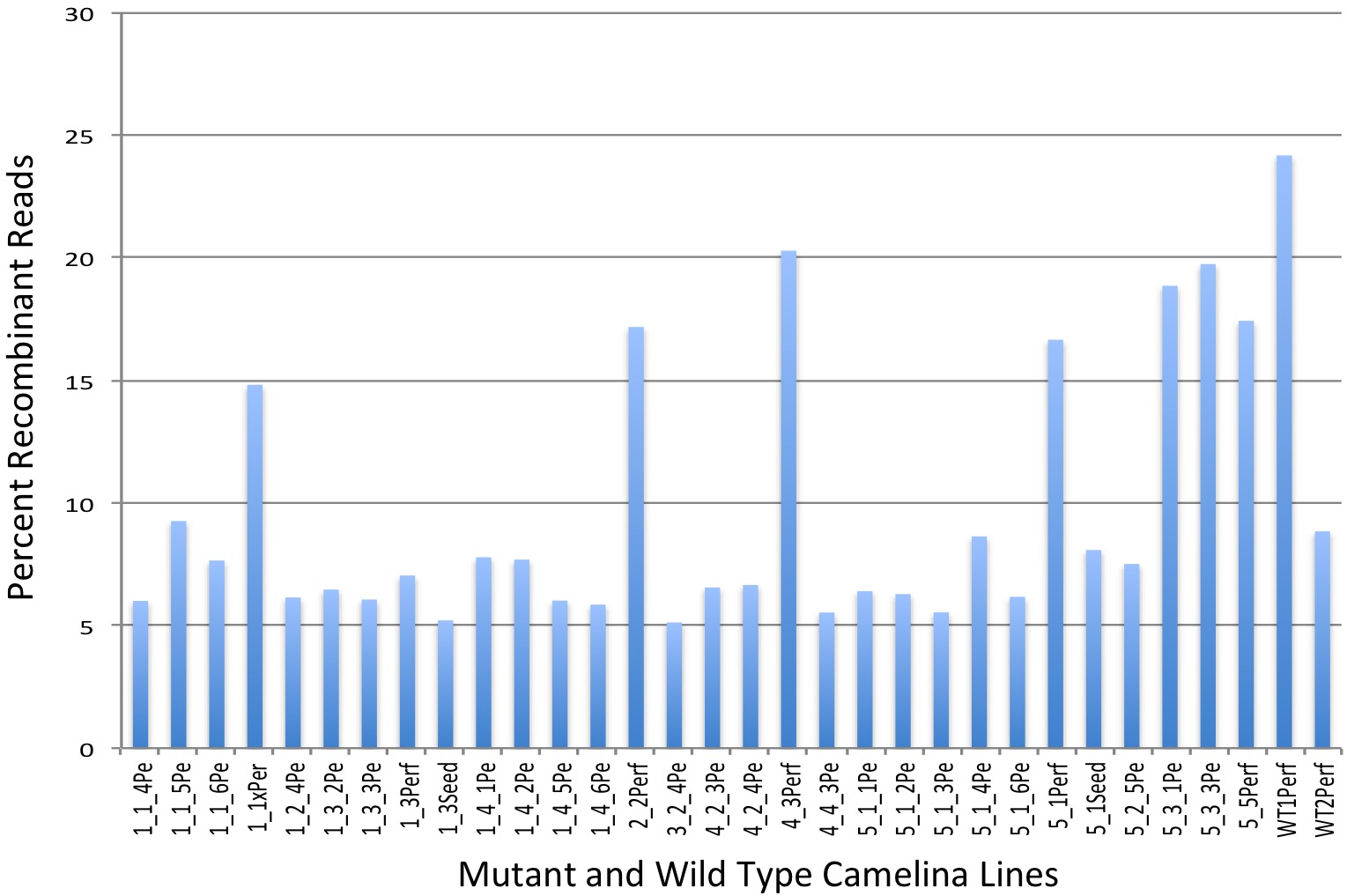


**SI Figure S3. Comparison of the rates of PCR recombinants during PCR amplification of Camelina *FAD2* gene sequences using non-homoeologous specific amplification primers.** Wild type and transgenic samples containing Cas9/sgRNA-generated mutations were analyzed. Percent recombinant reads calculated as the Number of Recombinant Reads/(Number of Recombinant Reads + Number of Wild Type Reads).

**
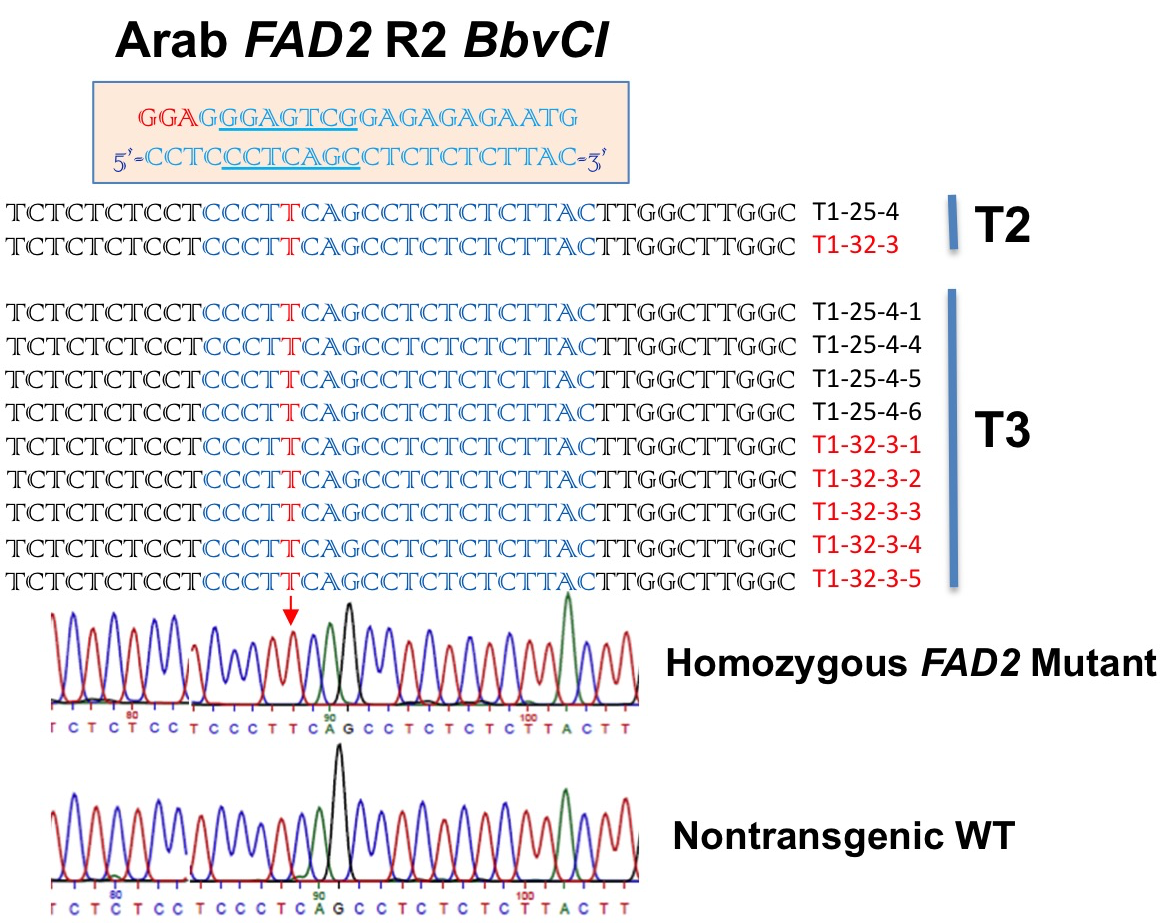
**

**SI Figure S4. Inheritance and homozygous condition of Cas9/sgRNA-mediated mutations at the R2 *BbvCI* target site of the *FAD2* gene in T2 andT3 progeny of two transgenic Arabidopsis lines** (derived from parental lines T1- 25 and T1-32). Target DNA sequences shown were obtained from PCR amplicons of the target area of DNA isolated from leaves of a single plant. For the T2 and T3 plants shown, only a single DNA sequencing trace pattern was obtained for all plants (top tracing), demonstrating that both *FAD2* gene alleles were identical in each plant (i.e., the sequencing tracing of PCR-amplified DNA from the two divergent *FAD2* gene alleles of a heterozygous plant would change from a congruent tracing upstream of the mutation site in one of the alleles to two non-congruent traces downstream of the mutation site). PAM site (AGG) of Cas9/sgRNA target site shown in red.

**
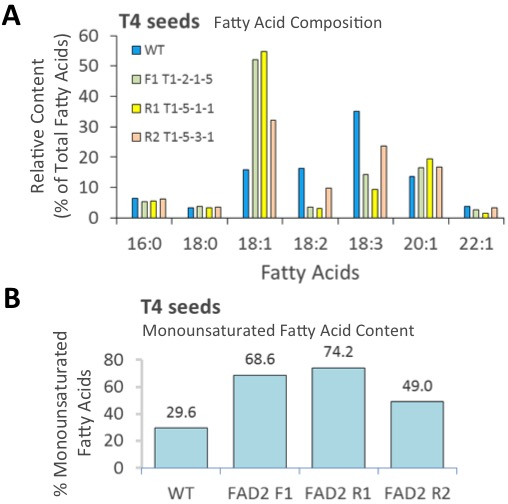
**

**SI Figure S5**. **Seed oil profiles in the best performing individual T4 seeds of Camelina plants transformed using Cas9/sgRNA targeting the R1, R2 and F1 sites in FAD2 genes.** A) Seed oil profiles in T4 seed from T3 plant T1-2-1-5 containing CsFAD2 genes targeted at site F1 (green color), in T1-5-1-1 targeted at site R1 (yellow), and in T1-5-3-1 targeted at site R2 (light red) are shown. WT: wild type control (blue). B) Total monounsaturated fatty acids (C18:1+C20:1 +C22:1) in the same best performing T4 seeds.


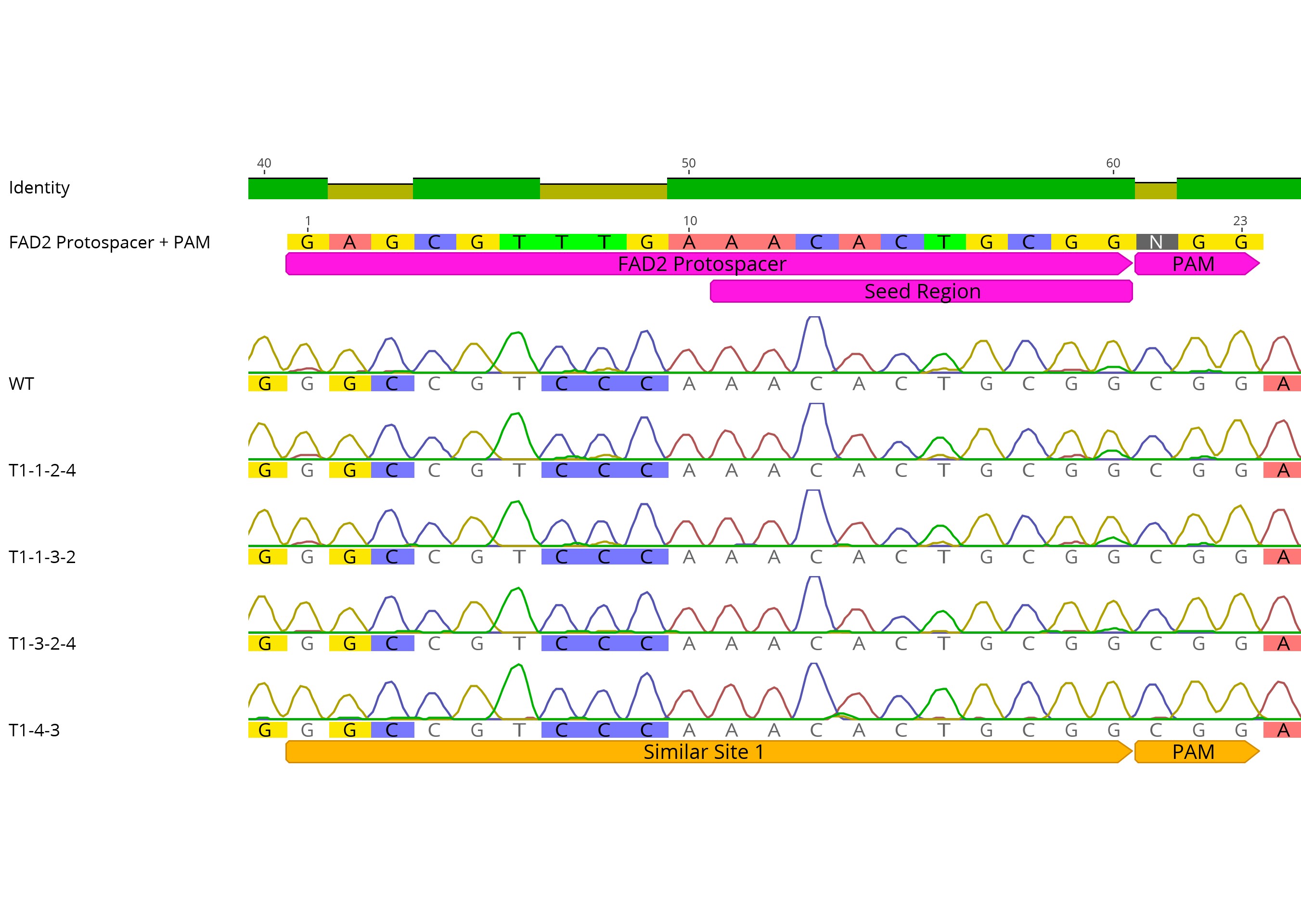


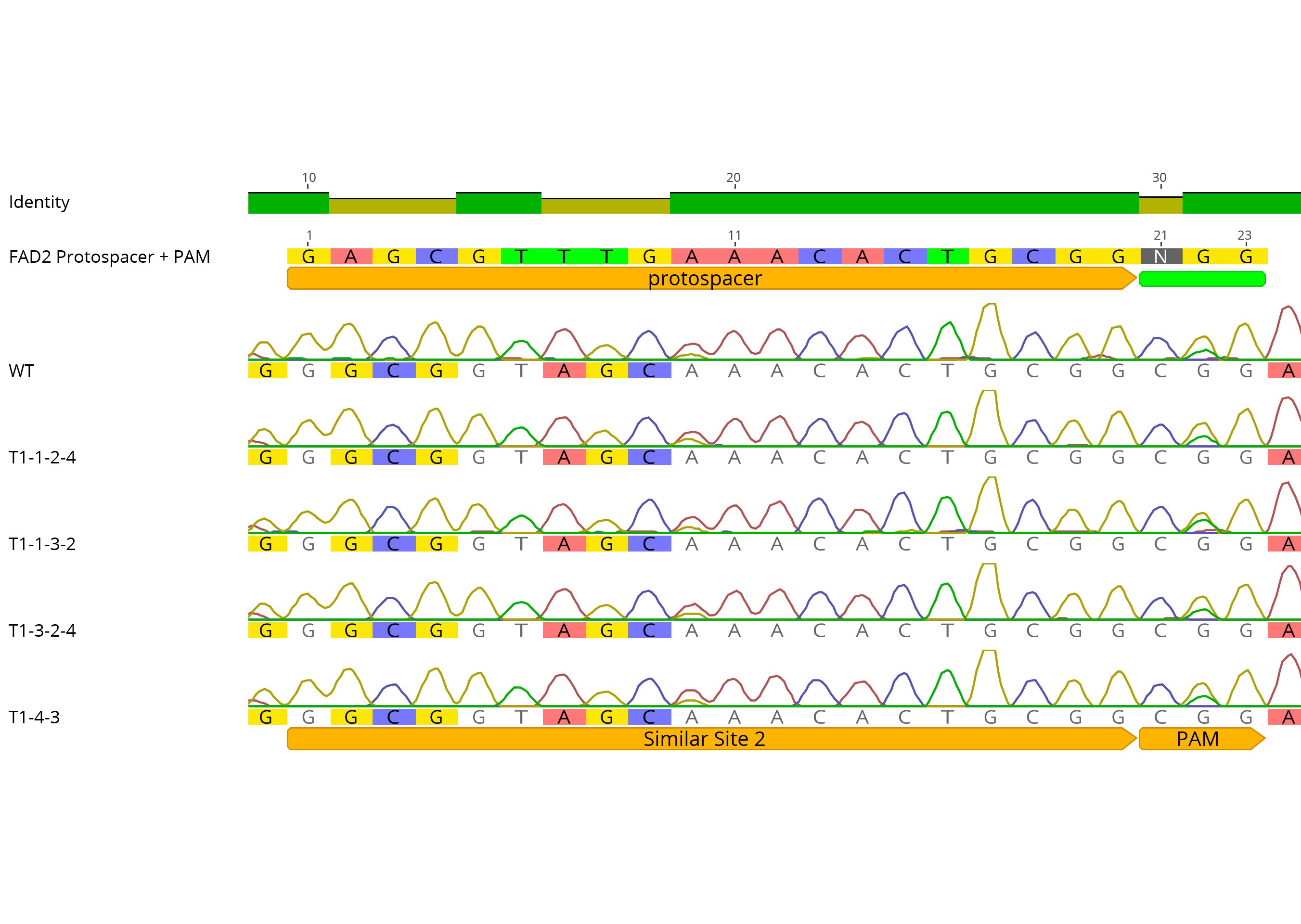
**
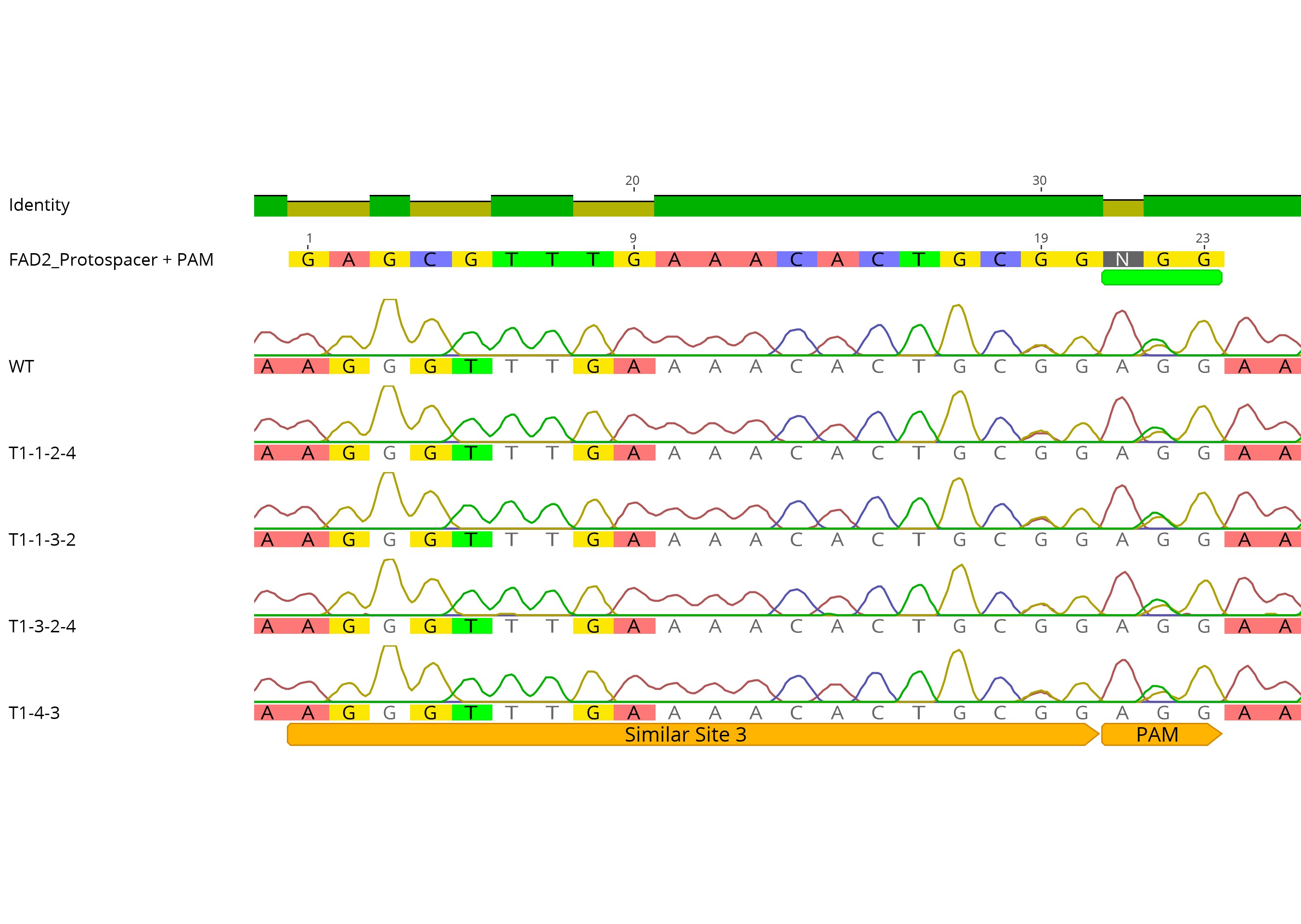
**

**SI Figure S6. DNA Sequencing of Potetial Off-Target Sites.** Sanger DNA sequences of 3 possible off-target sites in 4 lines that carry many on-target mutations. The WT and transgenic lines have indistinguishable chromatograms, suggesting that off-target mutations are not present.

**
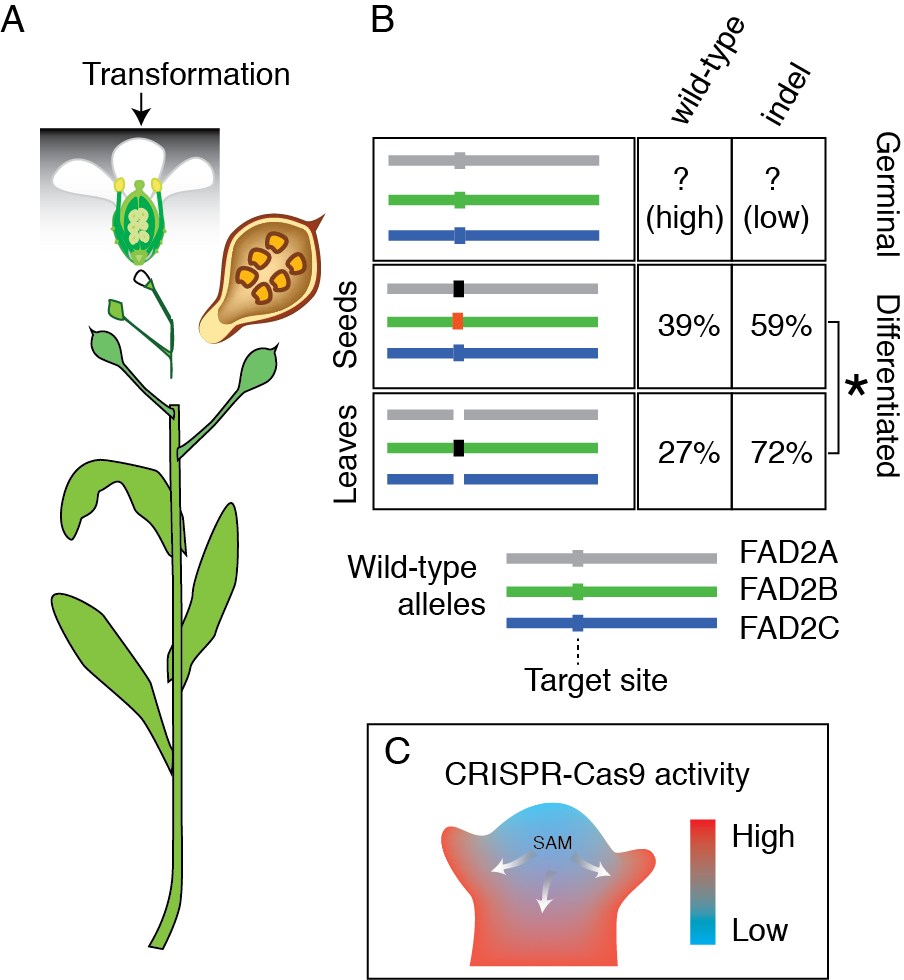
**

**SI Figure S7.** **Model of CRISPR-Cas9 action in hexaploid Camelina**.

**A.** Developmental stages of Camelina. **B.** Number of WT and Cas9 mutant alleles detected. Mutant alleles are more common in leaves (~72%) than in seed (~59%, *chi square test, P<0.0001). **C.** Hypothetical lower expression and/or activity of CRISPR-Cas9 in the shoot apical meristem (SAM) is consistent with the results and would explain the preponderant germinal transmission of wild-type alleles of the three target genes. The white arrows represent the transition of meristem cells into zones forming differentiated tissues and organs. Gametes originate from meristematic flower primordia.


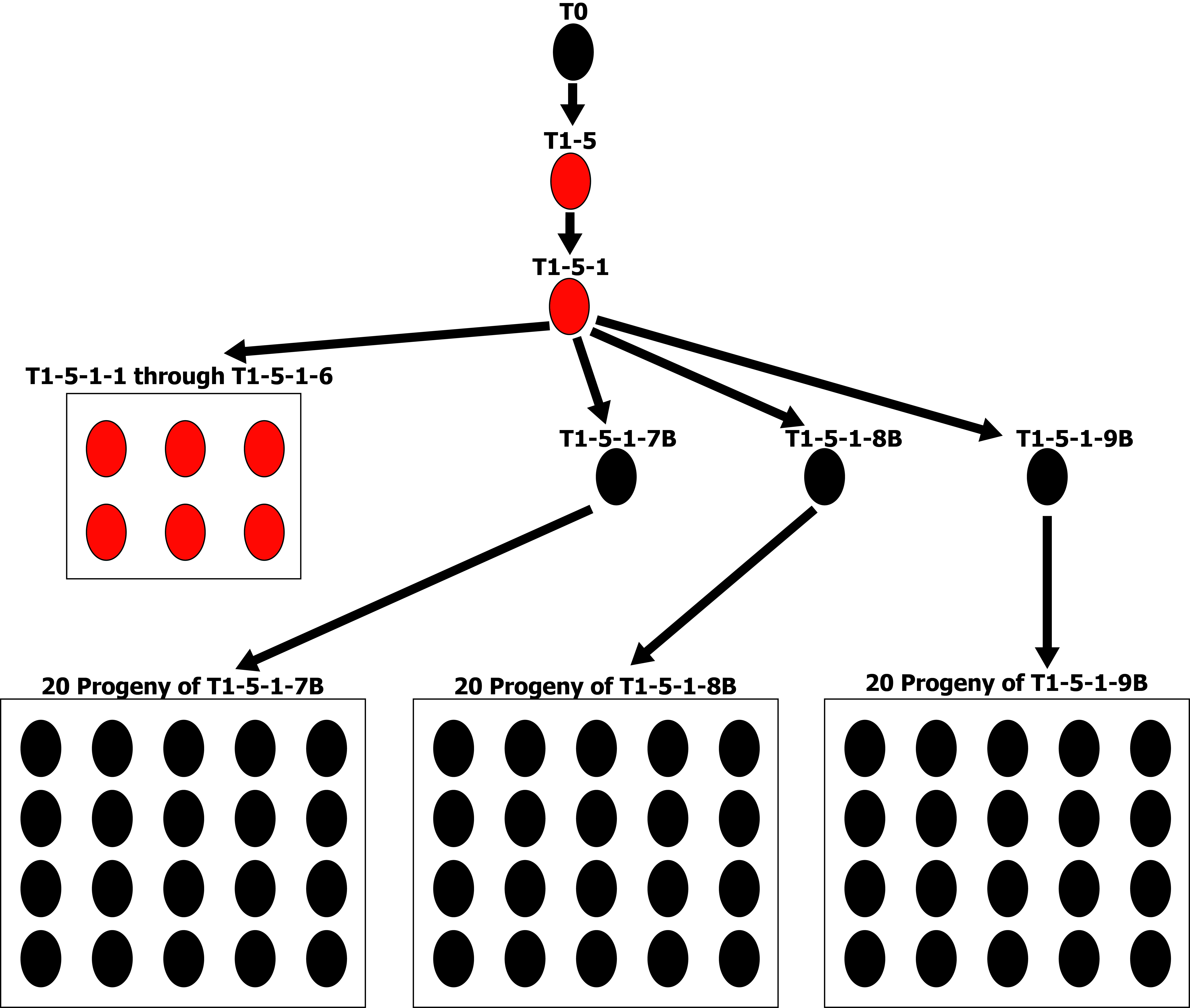


**SI Figure S8.** **Lineage of *Camelina sativa* plants used to analyze germline mutations**. Red ovals represent plants that are positive for the dsRED gene and the adjacent Cas-9 and sgRNA genes (verified by PCR analysis). Black oval represent seeds lacking the dsRed gene and adjacent Cas-9 and sgRNA genes (verified by PCR analysis – see SI Figure S8). *FAD2* gene amplicons from dsRED-positive plants were both Sanger sequenced and deep sequenced using Ampli-Seq. *FAD2* gene amplicons from progeny of dsRED-negative plants were Sanger sequenced.

**
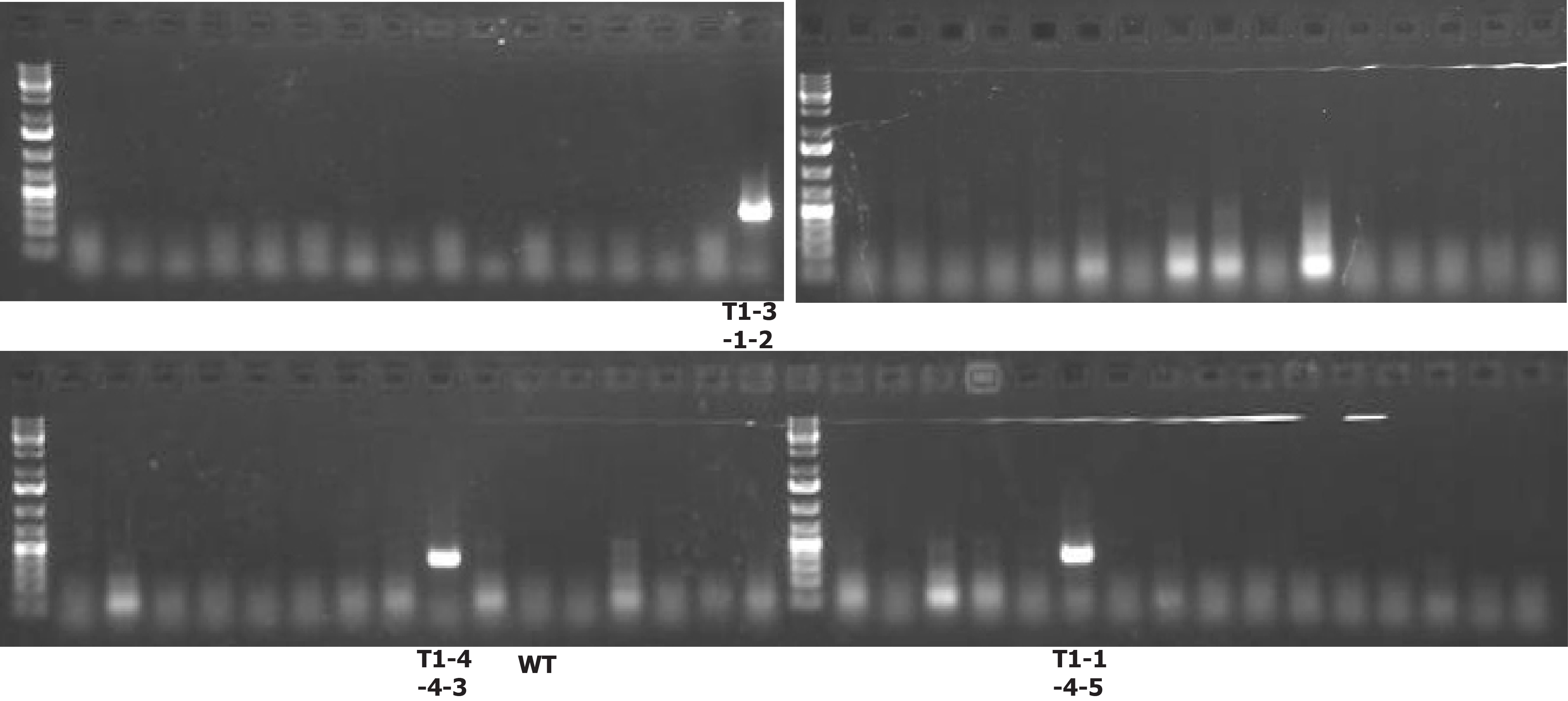
**

**SI Figure S9.** Lack of detection of the Cas9 transgene sequence in 20 individual progeny (unlabeled lanes) of each of 3 non-fluorescent (i.e., “black”) seeds: T1-5-1-7B, T1-5-1-8B, T1-5-1-9B. The positive controls are T1-4-4-3, T1-1-4-5 and T1-3-1-2, and the negative control is a WT individual. None of the 60 individual non-fluorescent progeny carried the Cas9 transgene.


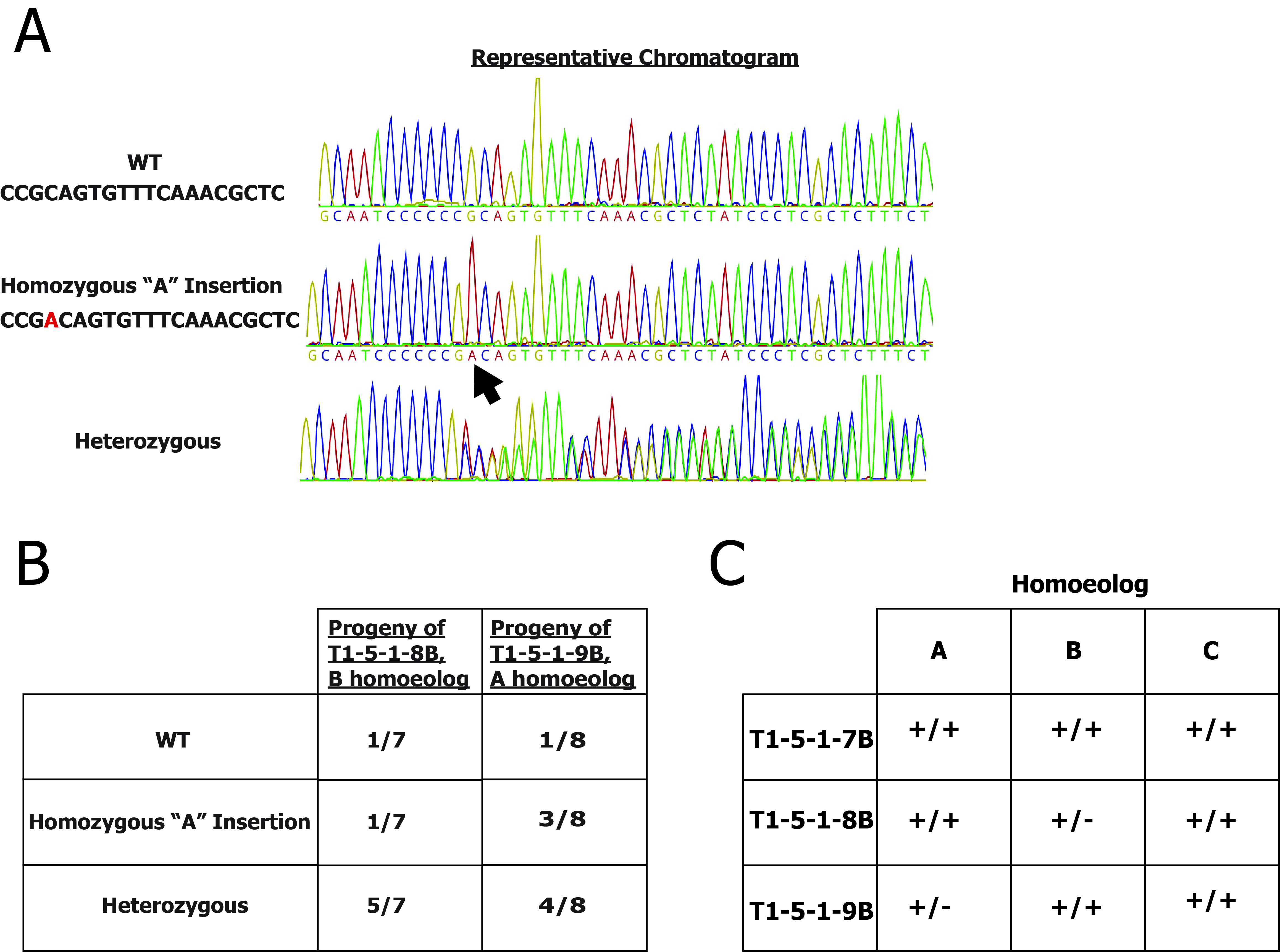


**SI Figure S10**. **Detection of germline FAD2 mutations**.

A. In the germline analysis all chromatograms from individual plants could be clearly designated as non-mutated (WT), containing an “A” insertion (“T” in the Ampliseq analysis) or heterozygous.

B. Frequency of mutated individuals.

C. FAD2 genotype of the three dsRED-negative plants (T1-5-1-7B, T1-5-1-8B and T1-5-9B), as inferred from the sequencing of their progeny. Two of the 18 alleles are mutated. These are presumed to be germline mutations. +, presence of a WT *FAD2*-containing homoeolog in T1-5 progeny lines; -, presence of a germline-inherited homoeolog containing a mutant, nonfunction *FAD2* gene in T1-5 progeny lines.

**Table S1. Primers upstream and downstream of the target sites in *FAD2* genes in Arabidopsis and Camelina** used for PCR amplification and Sanger sequencing of the target region. Letters in red indicate the recognition sites for the restriction enzymes, *SacI* and *BamHI*.

| **Site/restriction** | **Upstream primer (5’-3’)** | **Downstream primer (5’-3’)** | **Restriction Fragment (bp)** | **PCR fragment**  **(bp)** |
| --- | --- | --- | --- | --- |
| Arab FAD2/R1  *TauI* | TTTTTGAGCTCATGGGTGCAGGTGGAAGAATGC | TTTTTGGATCCAAGCATGAGGCTATAATGATGTC | 137/82 | 219 |
| Arab FAD2/R2  *BbvCI* | TTTTTGAGCTCATGGGTGCAGGTGGAAGAATGC | TTTTTGGATCCGATACCAGTTAGGACACAGCCTTGAC | 244/78 | 322 |
| Arab FAD2/F1  *AvaI* | TTTTTGAGCTCGAGATGAAGTATTTGTCCCAAAGCAG | TTTTTGGATCCGTAGATGGGAGCGTTGGGGAAG | 116/100 | 216 |
| Came FAD2/R1  *BtsI* | TTTTTGAGCTCATGGGTGCAGGTGGAAGAATGC | TTTTTGGATCCTTGGTGGCGACGTAGTAGAAGCAGGAG | 147/93 | 240 |
| Came FAD2/R2  *BbvCI* | TTTTTGAGCTCCACCGCAGTGTTTCAAACGCTCTATC | TTTTTGGATCCCTATGACCCAGACACCGGTTAGGAC | 123/88 | 211 |
| Came FAD2/F1  *AvaI* | TTTTTGAGCTCGAGATGAAGTATTTGTCCCAAAGCAG | TTTTTGGATCCGACGTTAAAGGCCAAGTAC | 116/43 | 159 |
| Came  Off Target 1 | TTATTGTGTCTCCACAAATGC | GAACAAAGTTGAAGTCTATGG |  | 290 |
| Came  Off Target 2 | ATGAGACACCCGAAGAGCAC | ACTTTGACGCCGACTGGTCTC |  | 359 |
| Came  Off Target 3 | CTCCAAACCCTCATTAAGACC | ATTGTTGTACTGTTGTGATAC |  | 292 |
| Came FAD2/R1  Homoeolog A | CGACTGTGTCATCGAGCCAC | TTCTCTGCTCTTTTTGTTCG |  | 601 |
| Came FAD2/R1  Homoeolog B | ACCACTTGATAGCTGATTTC | AAAATTTGTCAATTAGTGAT |  | 598 |
| Came FAD2/R1  Homoeolog C | GGCCAAGCCAAGTAAGAGAG | ATGCAAAATTTGTCAATTCC |  | 311 |

**SI Table S2**. **Rates of gene sequence chimera formation during PCR amplification of *FAD2* gene DNA sequences** using DNA samples obtained from wild type Camelina plants and plants expressing Cas9 and sgRNA genes - as determined with Illumina Amplicon-seq DNA sequencing.


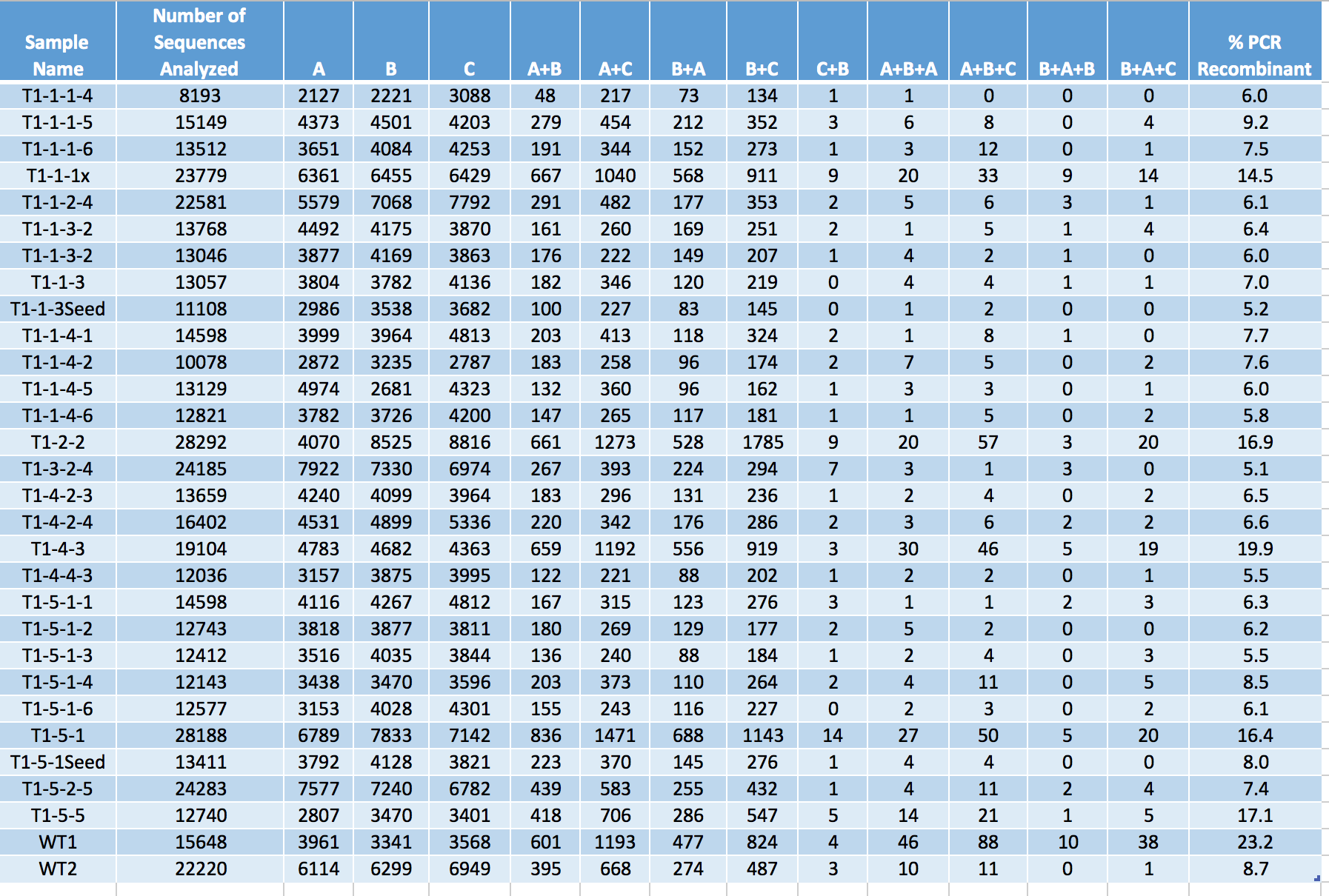


**Experimental Procedures**

**Preparation of Plant Materials**

*Arabidopsis thaliana* ecotype (Col-0) seeds were sown on soil and were stratified for 3 days at 4ºC. Seeds were geminated and plants were grown as described previously (Jiang et al., 2014). For transformation of Arabidopsis, *Agrobacterium tumefaciens*, strain C58cc, carrying the Cas9/sgRNA genes, were grown overnight at 27ºC with shaking at 200 RPM in 5 mL of Luria–Bertani (LB) medium supplemented with appropriate antibiotics. The next afternoon, 1.5 mL of the saturated bacterial culture was transferred to 500 mL of LB medium containing appropriate antibiotics and grown with shaking overnight at 27ºC. Cells were harvested the next day by centrifugation and diluted in 5% sucrose buffer to a final OD_600_ of 0.8. Silwet L-77 was added to a final concentration of 0.01%. Inflorescence clusters of Arabidopsis plants were dipped into the bacterial culture following the procedure of Zhang et al. (2006).

Camelina (*C. sativa*) cv. Suneson were grown with 14-h day length (24–26 °C) and 8-h dark (18–20 °C) with natural and supplemental lighting at 400–500 lmoles/m^2^/s in greenhouse condition (Nguyen et al., 2013). Transgenic Camelina lines were generated using *Agrobacterium* *tumefaciens* as described by Lu and Kang (2008). Both DsRed-positive Arabidopsis and Camelina seeds were identified using a green LED flashlight and a red camera filter lens to detect fluorescent seeds (Lu and Kang, 2008)

**Construction of Plant Expression Vectors**

*Arabidopsis thaliana* ecotype (Col-0) contains one copy of the fatty acid desaturase 2 gene (*FAD2*) gene (NCBI Reference Sequence: NM_112047.3). Three 19 to 20 nucleotide (nt) Cas9/sgRNA19 target sites were chosen near the 5’ terminus of the *FAD2* gene. Each was chosen to contain a restriction enzyme cleavage site (*TauI, BbvCI* or *AvaI*) within the predicted Cas9/sgRNA cleavage site immediately (3 base pairs) upstream of the PAM site. The target sites and sequences are show in Figure 1. *C. sativa* (Camelina) cv. Suneson, contains three copies each of the *FAD2* genes [Hutcheon et al., 2010; Kang et al., 2011; Nguyen et al., 2013; Camelina acyl lipid metabolism gene database (www.camelinagenome.org)]. Similarly, three identical target sites near the 5’ terminus of *FAD2* genes (with *BtsI, BbcVI* and *AvaI* cleavage sites, respectively) were chosen for sgRNA targeting (Figure 1).

A hygromycin resistance (hygro^r^) gene (GenBank accession no. AF234296.1; protein identifier [ID], AAF65337.1) under the control of the cauliflower mosaic virus (CaMV) *35S* gene promoter and terminator regions and an ER red fluorescence protein DsRed2 gene (GenBank: EU016077.1) under the control of the cassava mosaic virus (CsMV) *35S* gene promoter and terminator regions were used as markers for selection of transgenic Arabidopsis or Camelina plants. The CaMV 35S promoter region and termination region from the *Agrobacterium tumefaciens* T-DNA nopoline synthase gene was used to drive and terminate the expression of the Cas9 gene from *Streptococcus pyogenes* (Jiang et al., 2013). Transcription of the sgRNA gene is controlled by the Arabidopsis U6 gene promoter (U6P) and termination (U6T) regions and the DsRed2 red fluorescence gene by cassava mosaic virus (Cs) *35S* promoter and termination regions.

Design and construction of the Cas9, sgRNA for targeting different sites in *FAD2* genes in Arabidopsis and Camelina and the *Agrobacterium tumefaciens* binary vector containing these gene constructs were as described earlier (Jiang et al., 2013). Detailed target sequences and the arrangement of genes and regulatory elements within the T-DNA region of the binary vector are displayed in Figure 1. Notably, two target sequence in the *FAD2* gene are identical for both Arabidopsis and Camelina. The full DNA sequences of these binary vectors employed are listed in Supporting Information.

**Analyses of sgRNA target area using PCR amplification and restriction enzyme digestion to detect mutagenized DNA**

RE/PCR analyses of amplified regions of target genes in transgenic Arabidopsis and Camelina plants were performed as described previously (Jiang et al., 2013 and 2014). Upstream and downstream paired primers for PCR amplification of the sgRNA target regions in the *FAD2* genes as well as restriction enzymes used for the cleavage analyses of the PCR products are provided in Table S1. Typically, a mixture of genomic DNA from 3 plants was used as template for performing PCR amplifications for RE/PCR analyses for T1 and some T2 plants.

**Sanger-type DNA sequencing of PCR amplified Cas9/sgRNA target sites or of cloned PCR amplicons of target sites**

For most of the T2 and all T3 Arabidopsis plants, genomic DNA isolated from a single plant was used as template for PCR amplification and the amplified fragments were subjected to direct Sanger DNA sequencing. In other cases, the PCR amplicons were cloned into pBlueScript for subsequent Sanger DNA sequencing (Eurofins, Huntsville, AL 35805 USA).

**Search for Off-Target Mutations**

Twelve possible off-target sites were identified by creating a list of all sites in the *C. sativa* genome that have a PAM site and complete homology with the 10 bp “seed region” that is the 3’ end of the FAD2/R1 protospacer (Jiang et al., 2015). From those 12 sites, the three sites with the fewest mismatches to the rest of the protospacer were chosen for Sanger sequencing (SI Figure S6). There is only one copy of each of the 3 chosen off-target sites in the *C. sativa* hexaploid genome. PCR primers are listed in Table S1. The DNA templates are WT and Cas9-positive *C. sativa* gDNA from plants T1-1-2-4, T1-1-3-2, T1-3-2-4 and T1-4-3, which were shown to contain many mutations in the Ampliseq analysis (Data Set #4).

**Illumina Amplicon-MiSeq DNA sequencing and data analyses**

A subset of the DNA samples used for Sanger DNA sequencing were also used for Illumina Amplicon-MiSeq DNA sequencing. Forward and reverse primers for PCR amplification of a 394 bp sequence (350 bps after primer removal) containing the Cas9/sgRNA target site in the Camelina *FAD2* R1 target region were as follows: 5’-adaptor -GGAAGGAATGGAAGATAAGACC and 5’-adaptor -ATGGGTGCAGGTGGAAGAATGC. Amplicons were then subjected to a second round of PCR to add to add Illumina-compatible adaptors, as described previously (Jacobs et al., 2015). Libraries were submitted for 250PE sequencing on the Illumina MiSeq sequencer. DNA sequencing reads from 30 DNA samples from plants expressing the Cas9 and sgRNA genes and from 2 technical replicates of a wild type plant were demultiplexed according to their index and using custom python scripts. Next, pairs of reads for which the ends overlapped were assembled to create full-length amplicons. Primer sequences were trimmed and the resulting sequences were catalogued. For each sample, all of the sequences were analyzed for the presence of indels at the target site and the combination of homoeologous SNPs along the length of the sequence.

Recombinant sequences were identified as follows. Within each sample, the SNP combination was established for each read (average of ~16,000 reads per sample). For each SNP combination, the number of reads were counted. If a SNP combination was present in ≤ 10 reads across all samples, those sequences were removed from the analysis. Sequences containing at least 2 SNPs from only one homoeolog were designated as originating from that homoeolog; (for example AAAnnnAnnnnnnnAnnnn, where A are unambiguously from the A homoeologs and the rest of the sequence cannot be assigned to a particular homoeologs (n)). Sequences containing SNPs from 2 to 3 homoeologs, with at least 2 SNPs in a row for two homoeologs, were designated as Recombinant (for example AAAnnnAnnnnnBnnnnBn). The remaining reads were categorized as Ambiguous. Ambiguous reads were removed from the analysis. Removing ambiguous reads did not have a major effect. The percent of recombinant reads was calculated as: Number of Recombinant Reads/(Number of Recombinant Reads + Number of Wild Type Reads)X100%.

Analysis of the action of CRISPR/Cas9 was performed as follows. Only sequences that could be assigned to a specific homoeolog were retained. Each sequence was then reduced to a 35 bp fragment centered around the cut site, by searching for flanking kmers on either side of the target site ("GCTTTCTTCA" and "GAGAAAGAGC"). If one or both of these two kmers could not be found, the full-length sequence was retained. Identical sequences were pooled and the number of instance of each type were recorded, for each sample (see Supplementary Material, Table of Targets). Only sequences that were found at least 20 times total were analyzed. Each unique sequence was then analyzed further to determine the type of variation it presented (insertion, deletion, SNP or WT sequence). The composition of insertions and substitutions was visually compiled by comparing the mutated sequences to the corresponding WT sequence. Read count summaries are presented in the SI Data Set #4.

**Mutant identification, plant propagation and seeds fatty acid composition assays**

For both Arabidopsis and Camelina T1 plants, RE/PCR analyses or direct sequencing with PCR amplified targeting DNA fragments were evaluated for potential mutagenized DNA at Cas9/sgRNA target sites of *FAD2* genes. Typically, seeds of each of twelve to eighteen of these mutant T1 lines were propagated to the T2 generation. In turn, six to twelve individual plants of each T2 line were assessed using RE/PCR analysis or direct sequencing to detect mutations at the DNA target area. Seeds of T2 plants with verified mutations were propagated to the T3 generation. In turn, T4 seeds from 6 to18 individual T3 plants from each line were evaluated for potential changes in their fatty acid composition (see below).. For those seeds with significantly higher oleic acid content compared to wild type seeds, the Cas9/sgRNA target site of the *FAD2* gene was analyzed for mutations using clones of DNA fragments PCR amplified from DNA extracted from the corresponding single T3 plant leaf tissue. Simultaneously, some of the T2 parents of the single T3 plants were analyzed for mutations at the *FAD2* target area in leaf tissue. The fatty acid composition of T3 seeds from these same plants was determined.

**Detection of Germline Mutations**

Progeny of Cas9-positive T1-5-1 were qualified as Cas9 positive or negative based on dsRED seed fluorescence. Three non-fluorescent seeds (T1-5-1-7B, T1-5-1-8B, T1-5-1-9B) germinated and were grown and selfed. Twenty progeny from each were then grown and analyzed for germinal mutations (SI Figure S8). PCR analysis confirmed the lack of Cas9 transgene in all 60 of these progeny (SI Figure S9). Next, each of the three sets of twenty Cas9-negative plants was divided into 5 pools of 4 plants. For each pool, the FAD2 target site was Sanger sequenced with homoeolog-specific primers that are listed in Table S1. For pools that showed mutations, randomly chosen individual plants were sequenced with the homoeolog-specific primers (SI Figure S9).

**Analysis of Arabidopsis and Camelina seed fatty acid composition**

Total fatty acid content of seeds was measured as described previously (Nguyen et al., 2013). Trimethylsulphonium hydroxide (TMSH; Butte, 1983) was used for fatty acid methyl esters (FAMEs) transesterification. In brief, single seeds from transgenic Camelina plants or multiple seeds (5-7) from an individual transgenic Arabidopsis plant were directly crushed in 50 µL of TMSH in glass GC vials before the addition of 400 µL of heptane. The sample vials were agitated at room temperature for one hour and FAMEs were analyzed by gas chromatography as described (Cahoon et al., 2006).

**Supporting Information References**

Butte, W.J. (1983) Rapid method for the determination of fatty acid profiles from fats and oils using trimethyl-sulphonium hydroxide for transesterification. *J. Chrom.* **261**, 142–145.

Cahoon, E.B., Dietrich, C.R., Meyer, K., Damude, H.G., Dyer, J.M., Kinney, A.J. (2006) Conjugated fatty acids accumulate to high levels in phospholipids of metabolically engineered soybean and Arabidopsis seeds. *Phytochemistry* **67**, 1166–1176.

Hutcheon, C., Ditt, R.F., Beilstein, M., Comai, L., Schroeder, J., Goldstein, E., Shewmaker, C.K., Nguyen, T., De Rocher1, J., Kiser, J. (2010) Polyploid genome of Camelina sativa revealed by isolation of fatty acid synthesis genes. *BMC Plant Biology* **10**, 233 (doi:10.1186/1471-2229-10-233).

Jacobs, T.B., LaFayette, P.R., Schmitz, R.J., Parrott, W.A. (2015) Targeted genome modifications in soybean with CRISPR/Cas9. *BMC Biotechnology* **15:16**, 1-10 (doi**:**10.1186/s12896-015-0131-2).

Jiang, W., Brueggeman, A.J., Horken, K.M., Plucinak, T.M., Weeks, D.P. (2014) Successful transient expression of Cas9/sgRNA genes in *Chlamydomonas reinhardtii*. *Eukaryotic Cells* doi: **10**, 1128/EC.00213-14).

Jiang, W., Yang, B., Weeks, D.P. (2014) Efficient CRISPR/Cas9-Mediated Gene Editing in Arabidopsis thaliana and Inheritance of Modified Genes in the T2 and T3 Generations. *PLoS ONE* **9(6)**, e99225. doi:10.1371/journal.pone.0099225.

Jiang, W., Zhou, H., Bi, H., Fromm, M., Yang, B., Weeks, D.P. (2013) Demonstration of CRISPR/Cas9/sgRNA-mediated targeted gene modification in Arabidopsis, tobacco, sorghum and rice. *Nucleic. Acids Res*. doi: **10**, 1093/nar/gkt780

Kang, J., Snapp, A.R., Lu, C. (2011) Identification of three genes encoding microsomal oleate desaturases (FAD2) from the oilseed crop *Camelina sativa*. *Plant Physiology* *and Biochemistry* **49**, 223-229.

Lu, C., Kang, J. (2008) Generation of transgenic plants of a potential oilseed

crop *Camelina sativa* by Agrobacterium-mediated transformation. *Plant Cell*

*Rep*. **27**, 273–278.

Nguyen, H.T., Silva, J.E., Podicheti, R., Macrander, J., Yang, W., Nazarenus, T.J., Nam, J-W., Jaworski, J.G., Lu, C., Scheffler, B.E., Mockaitis, K., Cahoon, E.B. (2013) Camelina seed transcriptome: a tool for meal and oil improvement and translational research. *Plant Biotechnology Journal* **11**, 759–769.

Zhang, X., Henriques, R., Lin, S., Niu, Q., Chua, N. (2006) Agrobacterium-mediated transformation of *Arabidopsis thaliana* using the floral dip method. *Nature Protocols* **1**, 1-16.

**DNA sequences of binary vectors used in this study**

**DNA sequence of binary vector containing Cas9/sgRNA genes targeting the Arabidopsis FAD2 R1 (*TauI*) site**

AATTCCATGGAGTCAAAGATTCAAATAGAGGACCTAACAGAACTCGCCGTAAAGACTGGCGAACAGTTCATACAGAGTCTCTTACGACTCAATGACAAGAAGAAAATCTTCGTCAACATGGTGGAGCACGACACACTTGTCTACTCCAAAAATATCAAAGATACAGTCTCAGAAGACCAAAGGGCAATTGAGACTTTTCAACAAAGGGTAATATCCGGAAACCTCCTCGGATTCCATTGCCCAGCTATCTGTCACTTTATTGTGAAGATAGTGGAAAAGGAAGGTGGCTCCTACAAATGCCATCATTGCGATAAAGGAAAGGCCATCGTTGAAGATGCCTCTGCCGACAGTGGTCCCAAAGATGGACCCCCACCCACGAGGAGCATCGTGGAAAAAGAAGACGTTCCAACCACGTCTTCAAAGCAAGTGGATTGATGTGATATCTCCACTGACGTAAGGGATGACGCACAATCCCACTATCCTTCGCAAGACCCTTCCTCTATATAAGGAAGTTCATTTCATTTGGAGAGAACACGGGGGACTCTTGACCATGGTAGATCTGACTAGTGATTACAAGGACGATGATGACAAGAAAGACTATAAAGATGACGATGATAAGCATATGGACAAGAAGTACAGCATCGGCCTGGACATCGGCACGAACTCGGTGGGCTGGGCGGTGATCACGGACGAGTACAAGGTGCCCTCCAAGAAGTTCAAGGTGCTGGGCAACACCGACCGCCACTCGATCAAGAAGAACCTGATCGGCGCCCTGCTGTTCGACTCCGGCGAGACCGCCGAGGCGACGCGCCTGAAGCGCACCGCGCGTCGCCGCTACACGCGTCGCAAGAACCGCATCTGCTACCTGCAGGAGATCTTCAGCAACGAGATGGCCAAGGTGGACGACTCGTTCTTCCACCGCCTGGAGGAGTCCTTCCTGGTGGAGGAAGACAAGAAGCACGAGCGCCACCCCATCTTCGGCAACATCGTGGACGAGGTGGCCTACCACGAGAAGTACCCGACGATCTACCACCTGCGCAAGAAGCTGGTGGACAGCACCGACAAGGCGGACCTGCGCCTGATCTACCTGGCCCTGGCGCACATGATCAAGTTCCGCGGCCACTTCCTGATCGAGGGCGACCTGAACCCCGACAACTCGGACGTGGACAAGCTGTTCATCCAGCTGGTGCAGACCTACAACCAGCTGTTCGAGGAGAACCCGATCAACGCCTCCGGCGTGGACGCCAAGGCGATCCTGAGCGCGCGCCTGTCCAAGAGCCGTCGCCTGGAGAACCTGATCGCCCAGCTGCCCGGCGAGAAGAAGAACGGCCTGTTCGGCAACCTGATCGCGCTGTCGCTGGGCCTGACGCCGAACTTCAAGTCCAACTTCGACCTGGCCGAGGACGCGAAGCTGCAGCTGAGCAAGGACACCTACGACGACGACCTGGACAACCTGCTGGCCCAGATCGGCGACCAGTACGCGGACCTGTTCCTGGCCGCGAAGAACCTGTCGGACGCCATCCTGCTGTCCGACATCCTGCGCGTGAACACCGAGATCACGAAGGCCCCCCTGTCGGCGTCCATGATCAAGCGCTACGACGAGCACCACCAGGACCTGACCCTGCTGAAGGCGCTGGTGCGCCAGCAGCTGCCGGAGAAGTACAAGGAGATCTTCTTCGACCAGAGCAAGAACGGCTACGCCGGCTACATCGACGGCGGCGCGTCGCAAGAGGAGTTCTACAAGTTCATCAAGCCCATCCTGGAGAAGATGGACGGCACGGAGGAGCTGCTGGTGAAGCTGAACCGCGAGGACCTGCTGCGCAAGCAGCGCACCTTCGACAACGGCAGCATCCCCCACCAGATCCACCTGGGCGAGCTGCACGCCATCCTGCGTCGCCAAGAGGACTTCTACCCGTTCCTGAAGGACAACCGCGAGAAGATCGAGAAGATCCTGACGTTCCGCATCCCCTACTACGTGGGCCCGCTGGCCCGCGGCAACAGCCGCTTCGCGTGGATGACCCGCAAGTCGGAGGAGACCATCACGCCCTGGAACTTCGAGGAAGTGGTGGACAAGGGCGCCAGCGCGCAGTCGTTCATCGAGCGCATGACCAACTTCGACAAGAACCTGCCCAACGAGAAGGTGCTGCCGAAGCACTCCCTGCTGTACGAGTACTTCACCGTGTACAACGAGCTGACGAAGGTGAAGTACGTGACCGAGGGCATGCGCAAGCCCGCCTTCCTGAGCGGCGAGCAGAAGAAGGCGATCGTGGACCTGCTGTTCAAGACCAACCGCAAGGTGACGGTGAAGCAGCTGAAAGAGGACTACTTCAAGAAGATCGAGTGCTTCGACAGCGTGGAGATCTCGGGCGTGGAGGACCGCTTCAACGCCAGCCTGGGCACCTACCACGACCTGCTGAAGATCATCAAGGACAAGGACTTCCTGGACAACGAGGAGAACGAGGACATCCTGGAGGACATCGTGCTGACCCTGACGCTGTTCGAGGACCGCGAGATGATCGAGGAGCGCCTGAAGACGTACGCCCACCTGTTCGACGACAAGGTGATGAAGCAGCTGAAGCGTCGCCGCTACACCGGCTGGGGCCGCCTGAGCCGCAAGCTGATCAACGGCATCCGCGACAAGCAGTCCGGCAAGACCATCCTGGACTTCCTGAAGAGCGACGGCTTCGCGAACCGCAACTTCATGCAGCTGATCCACGACGACTCGCTGACCTTCAAAGAGGACATCCAGAAGGCCCAGGTGTCGGGCCAGGGCGACTCCCTGCACGAGCACATCGCCAACCTGGCGGGCTCCCCCGCGATCAAGAAGGGCATCCTGCAGACCGTGAAGGTGGTGGACGAGCTGGTGAAGGTGATGGGCCGCCACAAGCCGGAGAACATCGTGATCGAGATGGCCCGCGAGAACCAGACCACGCAGAAGGGCCAGAAGAACAGCCGCGAGCGCATGAAGCGCATCGAGGAAGGCATCAAGGAGCTGGGCTCGCAGATCCTGAAGGAGCACCCCGTGGAGAACACCCAGCTGCAGAACGAGAAGCTGTACCTGTACTACCTGCAGAACGGCCGCGACATGTACGTGGACCAGGAGCTGGACATCAACCGCCTGTCCGACTACGACGTGGACCACATCGTGCCCCAGAGCTTCCTGAAGGACGACTCGATCGACAACAAGGTGCTGACCCGCAGCGACAAGAACCGCGGCAAGAGCGACAACGTGCCGTCGGAGGAAGTGGTGAAGAAGATGAAGAACTACTGGCGCCAGCTGCTGAACGCCAAGCTGATCACGCAGCGCAAGTTCGACAACCTGACCAAGGCCGAGCGCGGTGGCCTGTCGGAGCTGGACAAGGCGGGCTTCATCAAGCGCCAGCTGGTGGAGACCCGCCAGATCACGAAGCACGTGGCGCAGATCCTGGACTCCCGCATGAACACGAAGTACGACGAGAACGACAAGCTGATCCGCGAGGTGAAGGTGATCACCCTGAAGTCCAAGCTGGTCAGCGACTTCCGCAAGGACTTCCAGTTCTACAAGGTGCGCGAGATCAACAACTACCACCACGCCCACGACGCGTACCTGAACGCCGTGGTGGGCACCGCGCTGATCAAGAAGTACCCCAAGCTGGAGAGCGAGTTCGTGTACGGCGACTACAAGGTGTACGACGTGCGCAAGATGATCGCCAAGTCGGAGCAGGAGATCGGCAAGGCCACCGCGAAGTACTTCTTCTACTCCAACATCATGAACTTCTTCAAGACCGAGATCACGCTGGCCAACGGCGAGATCCGCAAGCGCCCGCTGATCGAGACCAACGGCGAGACGGGCGAGATCGTGTGGGACAAGGGCCGCGACTTCGCGACCGTGCGCAAGGTGCTGAGCATGCCCCAGGTGAACATCGTGAAGAAGACCGAGGTGCAGACGGGCGGCTTCTCCAAGGAGAGCATCCTGCCGAAGCGCAACTCGGACAAGCTGATCGCCCGCAAGAAGGACTGGGACCCCAAGAAGTACGGCGGCTTCGACTCCCCGACCGTGGCCTACAGCGTGCTGGTGGTGGCGAAGGTGGAGAAGGGCAAGTCCAAGAAGCTGAAGAGCGTGAAGGAGCTGCTGGGCATCACCATCATGGAGCGCAGCTCGTTCGAGAAGAACCCCATCGACTTCCTGGAGGCCAAGGGCTACAAAGAGGTGAAGAAGGACCTGATCATCAAGCTGCCGAAGTACTCGCTGTTCGAGCTGGAGAACGGCCGCAAGCGCATGCTGGCCTCCGCGGGCGAGCTGCAGAAGGGCAACGAGCTGGCCCTGCCCAGCAAGTACGTGAACTTCCTGTACCTGGCGTCCCACTACGAGAAGCTGAAGGGCTCGCCGGAGGACAACGAGCAGAAGCAGCTGTTCGTGGAGCAGCACAAGCACTACCTGGACGAGATCATCGAGCAGATCTCGGAGTTCTCCAAGCGCGTGATCCTGGCCGACGCGAACCTGGACAAGGTGCTGAGCGCCTACAACAAGCACCGCGACAAGCCCATCCGCGAGCAGGCGGAGAACATCATCCACCTGTTCACCCTGACGAACCTGGGCGCCCCGGCCGCGTTCAAGTACTTCGACACCACGATCGACCGCAAGCGCTACACCTCCACGAAAGAGGTGCTGGACGCGACCCTGATCCACCAGAGCATCACCGGCCTGTACGAGACGCGCATCGACCTGAGCCAGCTGGGCGGCGACTCCCGCGCGGACCCGAAGAAGAAGCGCAAGGTGTAAGAATTAATTCGGATCGATCCATTGGTGACCAGCTCGAATTTCCCCGATCGTTCAAACATTTGGCAATAAAGTTTCTTAAGATTGAATCCTGTTGCCGGTCTTGCGATGATTATCATATAATTTCTGTTGAATTACGTTAAGCATGTAATAATTAACATGTAATGCATGACGTTATTTATGAGATGGGTTTTTATGATTAGAGTCCCGCAATTATACATTTAATACGCGATAGAAAACAAAATATAGCGCGCAAACTAGGATAAATTATCGCGCGCGGTGTCATCTATGTTACTAGATCGGGGGTACCCGTTGAACAACGGAAACTCGACTTGCCTTCCGCACAATACATCATTTCTTCTTAGCTTTTTTTCTTCTTCTTCGTTCATACAGTTTTTTTTTGTTTATCAGCTTACATTTTCTTGAACCGTAGCTTTCGTTTTCTTCTTTTTAACTTTCCATTCGGAGTTTTTGTATCTTGTTTCATAGTTTGTCCCAGGATTAGAATGATTAGGCATCGAACCTTCAAGAATTTGATTGAATAAAACATCTTCATTCTTAAGATATGAAGATAATCTTCAAAAGGCCCCTGGGAATCTGAAAGAAGAGAAGCAGGCCCATTTATATGGGAAAGAACAATAGTATTTCTTATATAGGCCCATTTAAGTTGAAAACAATCTTCAAAAGTCCCACATCGCTTAGATAAGAAAACGAAGCTGAGTTTATATACAGCTAGAGTCGAAGTAGTGATTGAGCGTTTGAAACAATGCGGCGTTTTAGAGCTAGAAATAGCAAGTTAAAATAAGGCTAGTCCGTTATCAACTTGAAAAAGTGGCACCGAGTCGGTGCTTTTTTTTTTTGCAAAATTTTCCAGATCGATTTCTTCTTCCTCTGTTCTTCGGCGTTCAATTTCTGGGTTTTTCTCTTCGTTTTCTGTAACTGAAACCTAAAATTTGACCTAAAAAAAATCTCAAATAATATGATTCAGTGGTTTTGTACTTTTCAGTTAGTTGAGTTTTGCAGTTCCGATGAGATAAACCAATAACTTTGCTTAGATCTAATTCATTCCGTTACACCTCTGATGGAGATGGAAGGTTCTTAATAATGATGCCATTTTTTGGGTAATAATTTTGAATTAGAATCAAGGGTATAAGATTCATAATTAACATCACTTAAGCAAAGTTCGTAATATACGACCACAGGATATAATTTTTGGTCTAGAGTCGACCTGCAGGCATGCAAGCTTGGCACTGGCCGTCGTTTTACAACGTCGTGACTGGGAAAACCCTGGCGTTACCCAACTTAATCGCCTTGCAGCACATCCCCCTTTCGCCAGCTGGCGTAATAGCGAAGAGGCCCGCACCGATCGCCCTTCCCAACAGTTGCGCAGCCTGAATGGCGAATGCTAGAGCAGCTTGAGCTTGGATCAGATTGTCGTTTCCCGCCTTCAGTTTAAACTATCAGTGTTTGACAGGATATATTGGCGGGTAAACCTAAGAGAAAAGAGCGTTTATTAGAATAACGGATATTTAAAAGGGCGTGAAAAGGTTTATCCGTTCGTCCATTTGTATGTGCATGCCAACCACAGGGTTCCCCTCGGGATCAAAGTACTTTGATCCAACCCCTCCGCTGCTATAGTGCAGTCGGCTTCTGACGTTCAGTGCAGCCGTCTTCTGAAAACGACATGTCGCACAAGTCCTAAGTTACGCGACAGGCTGCCGCCCTGCCCTTTTCCTGGCGTTTTCTTGTCGCGTGTTTTAGTCGCATAAAGTAGAATACTTGCGACTAGAACCGGAGACATTACGCCATGAACAAGAGCGCCGCCGCTGGCCTGCTGGGCTATGCCCGCGTCAGCACCGACGACCAGGACTTGACCAACCAACGGGCCGAACTGCACGCGGCCGGCTGCACCAAGCTGTTTTCCGAGAAGATCACCGGCACCAGGCGCGACCGCCCGGAGCTGGCCAGGATGCTTGACCACCTACGCCCTGGCGACGTTGTGACAGTGACCAGGCTAGACCGCCTGGCCCGCAGCACCCGCGACCTACTGGACATTGCCGAGCGCATCCAGGAGGCCGGCGCGGGCCTGCGTAGCCTGGCAGAGCCGTGGGCCGACACCACCACGCCGGCCGGCCGCATGGTGTTGACCGTGTTCGCCGGCATTGCCGAGTTCGAGCGTTCCCTAATCATCGACCGCACCCGGAGCGGGCGCGAGGCCGCCAAGGCCCGAGGCGTGAAGTTTGGCCCCCGCCCTACCCTCACCCCGGCACAGATCGCGCACGCCCGCGAGCTGATCGACCAGGAAGGCCGCACCGTGAAAGAGGCGGCTGCACTGCTTGGCGTGCATCGCTCGACCCTGTACCGCGCACTTGAGCGCAGCGAGGAAGTGACGCCCACCGAGGCCAGGCGGCGCGGTGCCTTCCGTGAGGACGCATTGACCGAGGCCGACGCCCTGGCGGCCGCCGAGAATGAACGCCAAGAGGAACAAGCATGAAACCGCACCAGGACGGCCAGGACGAACCGTTTTTCATTACCGAAGAGATCGAGGCGGAGATGATCGCGGCCGGGTACGTGTTCGAGCCGCCCGCGCACGTCTCAACCGTGCGGCTGCATGAAATCCTGGCCGGTTTGTCTGATGCCAAGCTGGCGGCCTGGCCGGCCAGCTTGGCCGCTGAAGAAACCGAGCGCCGCCGTCTAAAAAGGTGATGTGTATTTGAGTAAAACAGCTTGCGTCATGCGGTCGCTGCGTATATGATGCGATGAGTAAATAAACAAATACGCAAGGGGAACGCATGAAGGTTATCGCTGTACTTAACCAGAAAGGCGGGTCAGGCAAGACGACCATCGCAACCCATCTAGCCCGCGCCCTGCAACTCGCCGGGGCCGATGTTCTGTTAGTCGATTCCGATCCCCAGGGCAGTGCCCGCGATTGGGCGGCCGTGCGGGAAGATCAACCGCTAACCGTTGTCGGCATCGACCGCCCGACGATTGACCGCGACGTGAAGGCCATCGGCCGGCGCGACTTCGTAGTGATCGACGGAGCGCCCCAGGCGGCGGACTTGGCTGTGTCCGCGATCAAGGCAGCCGACTTCGTGCTGATTCCGGTGCAGCCAAGCCCTTACGACATATGGGCCACCGCCGACCTGGTGGAGCTGGTTAAGCAGCGCATTGAGGTCACGGATGGAAGGCTACAAGCGGCCTTTGTCGTGTCGCGGGCGATCAAAGGCACGCGCATCGGCGGTGAGGTTGCCGAGGCGCTGGCCGGGTACGAGCTGCCCATTCTTGAGTCCCGTATCACGCAGCGCGTGAGCTACCCAGGCACTGCCGCCGCCGGCACAACCGTTCTTGAATCAGAACCCGAGGGCGACGCTGCCCGCGAGGTCCAGGCGCTGGCCGCTGAAATTAAATCAAAACTCATTTGAGTTAATGAGGTAAAGAGAAAATGAGCAAAAGCACAAACACGCTAAGTGCCGGCCGTCCGAGCGCACGCAGCAGCAAGGCTGCAACGTTGGCCAGCCTGGCAGACACGCCAGCCATGAAGCGGGTCAACTTTCAGTTGCCGGCGGAGGATCACACCAAGCTGAAGATGTACGCGGTACGCCAAGGCAAGACCATTACCGAGCTGCTATCTGAATACATCGCGCAGCTACCAGAGTAAATGAGCAAATGAATAAATGAGTAGATGAATTTTAGCGGCTAAAGGAGGCGGCATGGAAAATCAAGAACAACCAGGCACCGACGCCGTGGAATGCCCCATGTGTGGAGGAACGGGCGGTTGGCCAGGCGTAAGCGGCTGGGTTGTCTGCCGGCCCTGCAATGGCACTGGAACCCCCAAGCCCGAGGAATCGGCGTGACGGTCGCAAACCATCCGGCCCGGTACAAATCGGCGCGGCGCTGGGTGATGACCTGGTGGAGAAGTTGAAGGCCGCGCAGGCCGCCCAGCGGCAACGCATCGAGGCAGAAGCACGCCCCGGTGAATCGTGGCAAGCGGCCGCTGATCGAATCCGCAAAGAATCCCGGCAACCGCCGGCAGCCGGTGCGCCGTCGATTAGGAAGCCGCCCAAGGGCGACGAGCAACCAGATTTTTTCGTTCCGATGCTCTATGACGTGGGCACCCGCGATAGTCGCAGCATCATGGACGTGGCCGTTTTCCGTCTGTCGAAGCGTGACCGACGAGCTGGCGAGGTGATCCGCTACGAGCTTCCAGACGGGCACGTAGAGGTTTCCGCAGGGCCGGCCGGCATGGCCAGTGTGTGGGATTACGACCTGGTACTGATGGCGGTTTCCCATCTAACCGAATCCATGAACCGATACCGGGAAGGGAAGGGAGACAAGCCCGGCCGCGTGTTCCGTCCACACGTTGCGGACGTACTCAAGTTCTGCCGGCGAGCCGATGGCGGAAAGCAGAAAGACGACCTGGTAGAAACCTGCATTCGGTTAAACACCACGCACGTTGCCATGCAGCGTACGAAGAAGGCCAAGAACGGCCGCCTGGTGACGGTATCCGAGGGTGAAGCCTTGATTAGCCGCTACAAGATCGTAAAGAGCGAAACCGGGCGGCCGGAGTACATCGAGATCGAGCTAGCTGATTGGATGTACCGCGAGATCACAGAAGGCAAGAACCCGGACGTGCTGACGGTTCACCCCGATTACTTTTTGATCGATCCCGGCATCGGCCGTTTTCTCTACCGCCTGGCACGCCGCGCCGCAGGCAAGGCAGAAGCCAGATGGTTGTTCAAGACGATCTACGAACGCAGTGGCAGCGCCGGAGAGTTCAAGAAGTTCTGTTTCACCGTGCGCAAGCTGATCGGGTCAAATGACCTGCCGGAGTACGATTTGAAGGAGGAGGCGGGGCAGGCTGGCCCGATCCTAGTCATGCGCTACCGCAACCTGATCGAGGGCGAAGCATCCGCCGGTTCCTAATGTACGGAGCAGATGCTAGGGCAAATTGCCCTAGCAGGGGAAAAAGGTCGAAAAGGTCTCTTTCCTGTGGATAGCACGTACATTGGGAACCCAAAGCCGTACATTGGGAACCGGAACCCGTACATTGGGAACCCAAAGCCGTACATTGGGAACCGGTCACACATGTAAGTGACTGATATAAAAGAGAAAAAAGGCGATTTTTCCGCCTAAAACTCTTTAAAACTTATTAAAACTCTTAAAACCCGCCTGGCCTGTGCATAACTGTCTGGCCAGCGCACAGCCGAAGAGCTGCAAAAAGCGCCTACCCTTCGGTCGCTGCGCTCCCTACGCCCCGCCGCTTCGCGTCGGCCTATCGCGGCCGCTGGCCGCTCAAAAATGGCTGGCCTACGGCCAGGCAATCTACCAGGGCGCGGACAAGCCGCGCCGTCGCCACTCGACCGCCGGCGCCCACATCAAGGCACCCTGCCTCGCGCGTTTCGGTGATGACGGTGAAAACCTCTGACACATGCAGCTCCCGGAGACGGTCACAGCTTGTCTGTAAGCGGATGCCGGGAGCAGACAAGCCCGTCAGGGCGCGTCAGCGGGTGTTGGCGGGTGTCGGGGCGCAGCCATGACCCAGTCACGTAGCGATAGCGGAGTGTATACTGGCTTAACTATGCGGCATCAGAGCAGATTGTACTGAGAGTGCACCATATGCGGTGTGAAATACCGCACAGATGCGTAAGGAGAAAATACCGCATCAGGCGCTCTTCCGCTTCCTCGCTCACTGACTCGCTGCGCTCGGTCGTTCGGCTGCGGCGAGCGGTATCAGCTCACTCAAAGGCGGTAATACGGTTATCCACAGAATCAGGGGATAACGCAGGAAAGAACATGTGAGCAAAAGGCCAGCAAAAGGCCAGGAACCGTAAAAAGGCCGCGTTGCTGGCGTTTTTCCATAGGCTCCGCCCCCCTGACGAGCATCACAAAAATCGACGCTCAAGTCAGAGGTGGCGAAACCCGACAGGACTATAAAGATACCAGGCGTTTCCCCCTGGAAGCTCCCTCGTGCGCTCTCCTGTTCCGACCCTGCCGCTTACCGGATACCTGTCCGCCTTTCTCCCTTCGGGAAGCGTGGCGCTTTCTCATAGCTCACGCTGTAGGTATCTCAGTTCGGTGTAGGTCGTTCGCTCCAAGCTGGGCTGTGTGCACGAACCCCCCGTTCAGCCCGACCGCTGCGCCTTATCCGGTAACTATCGTCTTGAGTCCAACCCGGTAAGACACGACTTATCGCCACTGGCAGCAGCCACTGGTAACAGGATTAGCAGAGCGAGGTATGTAGGCGGTGCTACAGAGTTCTTGAAGTGGTGGCCTAACTACGGCTACACTAGAAGGACAGTATTTGGTATCTGCGCTCTGCTGAAGCCAGTTACCTTCGGAAAAAGAGTTGGTAGCTCTTGATCCGGCAAACAAACCACCGCTGGTAGCGGTGGTTTTTTTGTTTGCAAGCAGCAGATTACGCGCAGAAAAAAAGGATCTCAAGAAGATCCTTTGATCTTTTCTACGGGGTCTGACGCTCAGTGGAACGAAAACTCACGTTAAGGGATTTTGGTCATGCATTCTAGGTACTAAAACAATTCATCCAGTAAAATATAATATTTTATTTTCTCCCAATCAGGCTTGATCCCCAGTAAGTCAAAAAATAGCTCGACATACTGTTCTTCCCCGATATCCTCCCTGATCGACCGGACGCAGAAGGCAATGTCATACCACTTGTCCGCCCTGCCGCTTCTCCCAAGATCAATAAAGCCACTTACTTTGCCATCTTTCACAAAGATGTTGCTGTCTCCCAGGTCGCCGTGGGAAAAGACAAGTTCCTCTTCGGGCTTTTCCGTCTTTAAAAAATCATACAGCTCGCGCGGATCTTTAAATGGAGTGTCTTCTTCCCAGTTTTCGCAATCCACATCGGCCAGATCGTTATTCAGTAAGTAATCCAATTCGGCTAAGCGGCTGTCTAAGCTATTCGTATAGGGACAATCCGATATGTCGATGGAGTGAAAGAGCCTGATGCACTCCGCATACAGCTCGATAATCTTTTCAGGGCTTTGTTCATCTTCATACTCTTCCGAGCAAAGGACGCCATCGGCCTCACTCATGAGCAGATTGCTCCAGCCATCATGCCGTTCAAAGTGCAGGACCTTTGGAACAGGCAGCTTTCCTTCCAGCCATAGCATCATGTCCTTTTCCCGTTCCACATCATAGGTGGTCCCTTTATACCGGCTGTCCGTCATTTTTAAATATAGGTTTTCATTTTCTCCCACCAGCTTATATACCTTAGCAGGAGACATTCCTTCCGTATCTTTTACGCAGCGGTATTTTTCGATCAGTTTTTTCAATTCCGGTGATATTCTCATTTTAGCCATTTATTATTTCCTTCCTCTTTTCTACAGTATTTAAAGATACCCCAAGAAGCTAATTATAACAAGACGAACTCCAATTCACTGTTCCTTGCATTCTAAAACCTTAAATACCAGAAAACAGCTTTTTCAAAGTTGTTTTCAAAGTTGGCGTATAACATAGTATCGACGGAGCCGATTTTGAAACCGCGGTGATCACAGGCAGCAACGCTCTGTCATCGTTACAATCAACATGCTACCCTCCGCGAGATCATCCGTGTTTCAAACCCGGCAGCTTAGTTGCCGTTCTTCCGAATAGCATCGGTAACATGAGCAAAGTCTGCCGCCTTACAACGGCTCTCCCGCTGACGCCGTCCCGGACTGATGGGCTGCCTGTATCGAGTGGTGATTTTGTGCCGAGCTGCCGGTCGGGGAGCTGTTGGCTGGCTGGTGGCAGGATATATTGTGGTGTAAACAAATTGACGCTTAGACAACTTAATAACACATTGCGGACGTTTTTAATGTACTGAATTAACGCCGAATTAATTCGGGGGATCTGGATTTTAGTACTGGATTTTGGTTTTAGGAATTAGAAATTTTATTGATAGAAGTATTTTACAAATACAAATACATACTAAGGGTTTCTTATATGCTCAACACATGAGCGAAACCCTATAGGAACCCTAATTCCCTTATCTGGGAACTACTCACACATTATTATGGAGAAACTCGAGCTTGTCGATCGACAGATCCGGTCGGCATCTACTCTATTTCTTTGCCCTCGGACGAGTGCTGGGGCGTCGGTTTCCACTATCGGCGAGTACTTCTACACAGCCATCGGTCCAGACGGCCGCGCTTCTGCGGGCGATTTGTGTACGCCCGACAGTCCCGGCTCCGGATCGGACGATTGCGTCGCATCGACCCTGCGCCCAAGCTGCATCATCGAAATTGCCGTCAACCAAGCTCTGATAGAGTTGGTCAAGACCAATGCGGAGCATATACGCCCGGAGTCGTGGCGATCCTGCAAGCTCCGGATGCCTCCGCTCGAAGTAGCGCGTCTGCTGCTCCATACAAGCCAACCACGGCCTCCAGAAGAAGATGTTGGCGACCTCGTATTGGGAATCCCCGAACATCGCCTCGCTCCAGTCAATGACCGCTGTTATGCGGCCATTGTCCGTCAGGACATTGTTGGAGCCGAAATCCGCGTGCACGAGGTGCCGGACTTCGGGGCAGTCCTCGGCCCAAAGCATCAGCTCATCGAGAGCCTGCGCGACGGACGCACTGACGGTGTCGTCCATCACAGTTTGCCAGTGATACACATGGGGATCAGCAATCGCGCATATGAAATCACGCCATGTAGTGTATTGACCGATTCCTTGCGGTCCGAATGGGCCGAACCCGCTCGTCTGGCTAAGATCGGCCGCAGCGATCGCATCCATAGCCTCCGCGACCGGTTGTAGAACAGCGGGCAGTTCGGTTTCAGGCAGGTCTTGCAACGTGACACCCTGTGCACGGCGGGAGATGCAATAGGTCAGGCTCTCGCTAAACTCCCCAATGTCAAGCACTTCCGGAATCGGGAGCGCGGCCGATGCAAAGTGCCGATAAACATAACGATCTTTGTAGAAACCATCGGCGCAGCTATTTACCCGCAGGACATATCCACGCCCTCCTACATCGAAGCTGAAAGCACGAGATTCTTCGCCCTCCGAGAGCTGCATCAGGTCGGAGACGCTGTCGAACTTTTCGATCAGAAACTTCTCGACAGACGTCGCGGTGAGTTCAGGCTTTTTCATATCTCATTGCCCCCCGGGATCTGCGAAAGCTCGAGAGAGATAGATTTGTAGAGAGAGACTGGTGATTTCAGCGTGTCCTCTCCAAATGAAATGAACTTCCTTATATAGAGGAAGGTCTTGCGAAGGATAGTGGGATTGTGCGTCATCCCTTACGTCAGTGGAGATATCACATCAATCCACTTGCTTTGAAGACGTGGTTGGAACGTCTTCTTTTTCCACGATGCTCCTCGTGGGTGGGGGTCCATCTTTGGGACCACTGTCGGCAGAGGCATCTTGAACGATAGCCTTTCCTTTATCGCAATGATGGCATTTGTAGGTGCCACCTTCCTTTTCTACTGTCCTTTTGATGAAGTGACAGATAGCTGGGCAATGGAATCCGAGGAGGTTTCCCGATATTACCCTTTGTTGAAAAGTCTCAATAGCCCTTTGGTCTTCTGAGACTGTATCTTTGATATTCTTGGAGTAGACGAGAGTGTCGTGCTCCACCATGTTATCACATCAATCCACTTGCTTTGAAGACGTGGTTGGAACGTCTTCTTTTTCCACGATGCTCCTCGTGGGTGGGGGTCCATCTTTGGGACCACTGTCGGCAGAGGCATCTTGAACGATAGCCTTTCCTTTATCGCAATGATGGCATTTGTAGGTGCCACCTTCCTTTTCTACTGTCCTTTTGATGAAGTGACAGATAGCTGGGCAATGGAATCCGAGGAGGTTTCCCGATATTACCCTTTGTTGAAAAGTCTCAATAGCCCTTTGGTCTTCTGAGACTGTATCTTTGATATTCTTGGAGTAGACGAGAGTGTCGTGCTCCACCATGTTGGCAAGCTGCTCTAGCCAATACGCAAACCGCCTCTCCCCGCGCGTTGGCCGATTCATTAATGCAGCTGGCACGACAGGTTTCCCGACTGGAAAGCGGGCAGTGAGCGCAACGCAATTAATGTGAGTTAGCTCACTCATTAGGCACCCCAGGCTTTACACTTTATGCTTCCGGCTCGTATGTTGTGTGGAATTGTGAGCGGATAACAATTTCACACAGGAAACAGCTATGACCATGATTACG

**DNA sequence of binary vector containing Cas9/sgRNA genes targeting the Camelina FAD2 R1 (*BtsI*) site**

AATTCCATGGAGTCAAAGATTCAAATAGAGGACCTAACAGAACTCGCCGTAAAGACTGGCGAACAGTTCATACAGAGTCTCTTACGACTCAATGACAAGAAGAAAATCTTCGTCAACATGGTGGAGCACGACACACTTGTCTACTCCAAAAATATCAAAGATACAGTCTCAGAAGACCAAAGGGCAATTGAGACTTTTCAACAAAGGGTAATATCCGGAAACCTCCTCGGATTCCATTGCCCAGCTATCTGTCACTTTATTGTGAAGATAGTGGAAAAGGAAGGTGGCTCCTACAAATGCCATCATTGCGATAAAGGAAAGGCCATCGTTGAAGATGCCTCTGCCGACAGTGGTCCCAAAGATGGACCCCCACCCACGAGGAGCATCGTGGAAAAAGAAGACGTTCCAACCACGTCTTCAAAGCAAGTGGATTGATGTGATATCTCCACTGACGTAAGGGATGACGCACAATCCCACTATCCTTCGCAAGACCCTTCCTCTATATAAGGAAGTTCATTTCATTTGGAGAGAACACGGGGGACTCTTGACCATGGTAGATCTGACTAGTGATTACAAGGACGATGATGACAAGAAAGACTATAAAGATGACGATGATAAGCATATGGACAAGAAGTACAGCATCGGCCTGGACATCGGCACGAACTCGGTGGGCTGGGCGGTGATCACGGACGAGTACAAGGTGCCCTCCAAGAAGTTCAAGGTGCTGGGCAACACCGACCGCCACTCGATCAAGAAGAACCTGATCGGCGCCCTGCTGTTCGACTCCGGCGAGACCGCCGAGGCGACGCGCCTGAAGCGCACCGCGCGTCGCCGCTACACGCGTCGCAAGAACCGCATCTGCTACCTGCAGGAGATCTTCAGCAACGAGATGGCCAAGGTGGACGACTCGTTCTTCCACCGCCTGGAGGAGTCCTTCCTGGTGGAGGAAGACAAGAAGCACGAGCGCCACCCCATCTTCGGCAACATCGTGGACGAGGTGGCCTACCACGAGAAGTACCCGACGATCTACCACCTGCGCAAGAAGCTGGTGGACAGCACCGACAAGGCGGACCTGCGCCTGATCTACCTGGCCCTGGCGCACATGATCAAGTTCCGCGGCCACTTCCTGATCGAGGGCGACCTGAACCCCGACAACTCGGACGTGGACAAGCTGTTCATCCAGCTGGTGCAGACCTACAACCAGCTGTTCGAGGAGAACCCGATCAACGCCTCCGGCGTGGACGCCAAGGCGATCCTGAGCGCGCGCCTGTCCAAGAGCCGTCGCCTGGAGAACCTGATCGCCCAGCTGCCCGGCGAGAAGAAGAACGGCCTGTTCGGCAACCTGATCGCGCTGTCGCTGGGCCTGACGCCGAACTTCAAGTCCAACTTCGACCTGGCCGAGGACGCGAAGCTGCAGCTGAGCAAGGACACCTACGACGACGACCTGGACAACCTGCTGGCCCAGATCGGCGACCAGTACGCGGACCTGTTCCTGGCCGCGAAGAACCTGTCGGACGCCATCCTGCTGTCCGACATCCTGCGCGTGAACACCGAGATCACGAAGGCCCCCCTGTCGGCGTCCATGATCAAGCGCTACGACGAGCACCACCAGGACCTGACCCTGCTGAAGGCGCTGGTGCGCCAGCAGCTGCCGGAGAAGTACAAGGAGATCTTCTTCGACCAGAGCAAGAACGGCTACGCCGGCTACATCGACGGCGGCGCGTCGCAAGAGGAGTTCTACAAGTTCATCAAGCCCATCCTGGAGAAGATGGACGGCACGGAGGAGCTGCTGGTGAAGCTGAACCGCGAGGACCTGCTGCGCAAGCAGCGCACCTTCGACAACGGCAGCATCCCCCACCAGATCCACCTGGGCGAGCTGCACGCCATCCTGCGTCGCCAAGAGGACTTCTACCCGTTCCTGAAGGACAACCGCGAGAAGATCGAGAAGATCCTGACGTTCCGCATCCCCTACTACGTGGGCCCGCTGGCCCGCGGCAACAGCCGCTTCGCGTGGATGACCCGCAAGTCGGAGGAGACCATCACGCCCTGGAACTTCGAGGAAGTGGTGGACAAGGGCGCCAGCGCGCAGTCGTTCATCGAGCGCATGACCAACTTCGACAAGAACCTGCCCAACGAGAAGGTGCTGCCGAAGCACTCCCTGCTGTACGAGTACTTCACCGTGTACAACGAGCTGACGAAGGTGAAGTACGTGACCGAGGGCATGCGCAAGCCCGCCTTCCTGAGCGGCGAGCAGAAGAAGGCGATCGTGGACCTGCTGTTCAAGACCAACCGCAAGGTGACGGTGAAGCAGCTGAAAGAGGACTACTTCAAGAAGATCGAGTGCTTCGACAGCGTGGAGATCTCGGGCGTGGAGGACCGCTTCAACGCCAGCCTGGGCACCTACCACGACCTGCTGAAGATCATCAAGGACAAGGACTTCCTGGACAACGAGGAGAACGAGGACATCCTGGAGGACATCGTGCTGACCCTGACGCTGTTCGAGGACCGCGAGATGATCGAGGAGCGCCTGAAGACGTACGCCCACCTGTTCGACGACAAGGTGATGAAGCAGCTGAAGCGTCGCCGCTACACCGGCTGGGGCCGCCTGAGCCGCAAGCTGATCAACGGCATCCGCGACAAGCAGTCCGGCAAGACCATCCTGGACTTCCTGAAGAGCGACGGCTTCGCGAACCGCAACTTCATGCAGCTGATCCACGACGACTCGCTGACCTTCAAAGAGGACATCCAGAAGGCCCAGGTGTCGGGCCAGGGCGACTCCCTGCACGAGCACATCGCCAACCTGGCGGGCTCCCCCGCGATCAAGAAGGGCATCCTGCAGACCGTGAAGGTGGTGGACGAGCTGGTGAAGGTGATGGGCCGCCACAAGCCGGAGAACATCGTGATCGAGATGGCCCGCGAGAACCAGACCACGCAGAAGGGCCAGAAGAACAGCCGCGAGCGCATGAAGCGCATCGAGGAAGGCATCAAGGAGCTGGGCTCGCAGATCCTGAAGGAGCACCCCGTGGAGAACACCCAGCTGCAGAACGAGAAGCTGTACCTGTACTACCTGCAGAACGGCCGCGACATGTACGTGGACCAGGAGCTGGACATCAACCGCCTGTCCGACTACGACGTGGACCACATCGTGCCCCAGAGCTTCCTGAAGGACGACTCGATCGACAACAAGGTGCTGACCCGCAGCGACAAGAACCGCGGCAAGAGCGACAACGTGCCGTCGGAGGAAGTGGTGAAGAAGATGAAGAACTACTGGCGCCAGCTGCTGAACGCCAAGCTGATCACGCAGCGCAAGTTCGACAACCTGACCAAGGCCGAGCGCGGTGGCCTGTCGGAGCTGGACAAGGCGGGCTTCATCAAGCGCCAGCTGGTGGAGACCCGCCAGATCACGAAGCACGTGGCGCAGATCCTGGACTCCCGCATGAACACGAAGTACGACGAGAACGACAAGCTGATCCGCGAGGTGAAGGTGATCACCCTGAAGTCCAAGCTGGTCAGCGACTTCCGCAAGGACTTCCAGTTCTACAAGGTGCGCGAGATCAACAACTACCACCACGCCCACGACGCGTACCTGAACGCCGTGGTGGGCACCGCGCTGATCAAGAAGTACCCCAAGCTGGAGAGCGAGTTCGTGTACGGCGACTACAAGGTGTACGACGTGCGCAAGATGATCGCCAAGTCGGAGCAGGAGATCGGCAAGGCCACCGCGAAGTACTTCTTCTACTCCAACATCATGAACTTCTTCAAGACCGAGATCACGCTGGCCAACGGCGAGATCCGCAAGCGCCCGCTGATCGAGACCAACGGCGAGACGGGCGAGATCGTGTGGGACAAGGGCCGCGACTTCGCGACCGTGCGCAAGGTGCTGAGCATGCCCCAGGTGAACATCGTGAAGAAGACCGAGGTGCAGACGGGCGGCTTCTCCAAGGAGAGCATCCTGCCGAAGCGCAACTCGGACAAGCTGATCGCCCGCAAGAAGGACTGGGACCCCAAGAAGTACGGCGGCTTCGACTCCCCGACCGTGGCCTACAGCGTGCTGGTGGTGGCGAAGGTGGAGAAGGGCAAGTCCAAGAAGCTGAAGAGCGTGAAGGAGCTGCTGGGCATCACCATCATGGAGCGCAGCTCGTTCGAGAAGAACCCCATCGACTTCCTGGAGGCCAAGGGCTACAAAGAGGTGAAGAAGGACCTGATCATCAAGCTGCCGAAGTACTCGCTGTTCGAGCTGGAGAACGGCCGCAAGCGCATGCTGGCCTCCGCGGGCGAGCTGCAGAAGGGCAACGAGCTGGCCCTGCCCAGCAAGTACGTGAACTTCCTGTACCTGGCGTCCCACTACGAGAAGCTGAAGGGCTCGCCGGAGGACAACGAGCAGAAGCAGCTGTTCGTGGAGCAGCACAAGCACTACCTGGACGAGATCATCGAGCAGATCTCGGAGTTCTCCAAGCGCGTGATCCTGGCCGACGCGAACCTGGACAAGGTGCTGAGCGCCTACAACAAGCACCGCGACAAGCCCATCCGCGAGCAGGCGGAGAACATCATCCACCTGTTCACCCTGACGAACCTGGGCGCCCCGGCCGCGTTCAAGTACTTCGACACCACGATCGACCGCAAGCGCTACACCTCCACGAAAGAGGTGCTGGACGCGACCCTGATCCACCAGAGCATCACCGGCCTGTACGAGACGCGCATCGACCTGAGCCAGCTGGGCGGCGACTCCCGCGCGGACCCGAAGAAGAAGCGCAAGGTGTAAGAATTAATTCGGATCGATCCATTGGTGACCAGCTCGAATTTCCCCGATCGTTCAAACATTTGGCAATAAAGTTTCTTAAGATTGAATCCTGTTGCCGGTCTTGCGATGATTATCATATAATTTCTGTTGAATTACGTTAAGCATGTAATAATTAACATGTAATGCATGACGTTATTTATGAGATGGGTTTTTATGATTAGAGTCCCGCAATTATACATTTAATACGCGATAGAAAACAAAATATAGCGCGCAAACTAGGATAAATTATCGCGCGCGGTGTCATCTATGTTACTAGATCGGGGGTACCCGTTGAACAACGGAAACTCGACTTGCCTTCCGCACAATACATCATTTCTTCTTAGCTTTTTTTCTTCTTCTTCGTTCATACAGTTTTTTTTTGTTTATCAGCTTACATTTTCTTGAACCGTAGCTTTCGTTTTCTTCTTTTTAACTTTCCATTCGGAGTTTTTGTATCTTGTTTCATAGTTTGTCCCAGGATTAGAATGATTAGGCATCGAACCTTCAAGAATTTGATTGAATAAAACATCTTCATTCTTAAGATATGAAGATAATCTTCAAAAGGCCCCTGGGAATCTGAAAGAAGAGAAGCAGGCCCATTTATATGGGAAAGAACAATAGTATTTCTTATATAGGCCCATTTAAGTTGAAAACAATCTTCAAAAGTCCCACATCGCTTAGATAAGAAAACGAAGCTGAGTTTATATACAGCTAGAGTCGAAGTAGTGATTGAGCGTTTGAAACACTGCGGGTTTTAGAGCTAGAAATAGCAAGTTAAAATAAGGCTAGTCCGTTATCAACTTGAAAAAGTGGCACCGAGTCGGTGCTTTTTTTTTTTGCAAAATTTTCCAGATCGATTTCTTCTTCCTCTGTTCTTCGGCGTTCAATTTCTGGGTTTTTCTCTTCGTTTTCTGTAACTGAAACCTAAAATTTGACCTAAAAAAAATCTCAAATAATATGATTCAGTGGTTTTGTACTTTTCAGTTAGTTGAGTTTTGCAGTTCCGATGAGATAAACCAATAACTTTGCTTAGATCTAATTCATTCCGTTACACCTCTGATGGAGATGGAAGGTTCTTAATAATGATGCCATTTTTTGGGTAATAATTTTGAATTAGAATCAAGGGTATAAGATTCATAATTAACATCACTTAAGCAAAGTTCGTAATATACGACCACAGGATATAATTTTTGGTCTAGAGTCGACCTGCAGGCATGCAAGCTTGGCACTGGCCGTCGTTTTACAACGTCGTGACTGGGAAAACCCTGGCGTTACCCAACTTAATCGCCTTGCAGCACATCCCCCTTTCGCCAGCTGGCGTAATAGCGAAGAGGCCCGCACCGATCGCCCTTCCCAACAGTTGCGCAGCCTGAATGGCGAATGCTAGAGCAGCTTGAGCTTGGATCAGATTGTCGTTTCCCGCCTTCAGTTTAAACTATCAGTGTTTGACAGGATATATTGGCGGGTAAACCTAAGAGAAAAGAGCGTTTATTAGAATAACGGATATTTAAAAGGGCGTGAAAAGGTTTATCCGTTCGTCCATTTGTATGTGCATGCCAACCACAGGGTTCCCCTCGGGATCAAAGTACTTTGATCCAACCCCTCCGCTGCTATAGTGCAGTCGGCTTCTGACGTTCAGTGCAGCCGTCTTCTGAAAACGACATGTCGCACAAGTCCTAAGTTACGCGACAGGCTGCCGCCCTGCCCTTTTCCTGGCGTTTTCTTGTCGCGTGTTTTAGTCGCATAAAGTAGAATACTTGCGACTAGAACCGGAGACATTACGCCATGAACAAGAGCGCCGCCGCTGGCCTGCTGGGCTATGCCCGCGTCAGCACCGACGACCAGGACTTGACCAACCAACGGGCCGAACTGCACGCGGCCGGCTGCACCAAGCTGTTTTCCGAGAAGATCACCGGCACCAGGCGCGACCGCCCGGAGCTGGCCAGGATGCTTGACCACCTACGCCCTGGCGACGTTGTGACAGTGACCAGGCTAGACCGCCTGGCCCGCAGCACCCGCGACCTACTGGACATTGCCGAGCGCATCCAGGAGGCCGGCGCGGGCCTGCGTAGCCTGGCAGAGCCGTGGGCCGACACCACCACGCCGGCCGGCCGCATGGTGTTGACCGTGTTCGCCGGCATTGCCGAGTTCGAGCGTTCCCTAATCATCGACCGCACCCGGAGCGGGCGCGAGGCCGCCAAGGCCCGAGGCGTGAAGTTTGGCCCCCGCCCTACCCTCACCCCGGCACAGATCGCGCACGCCCGCGAGCTGATCGACCAGGAAGGCCGCACCGTGAAAGAGGCGGCTGCACTGCTTGGCGTGCATCGCTCGACCCTGTACCGCGCACTTGAGCGCAGCGAGGAAGTGACGCCCACCGAGGCCAGGCGGCGCGGTGCCTTCCGTGAGGACGCATTGACCGAGGCCGACGCCCTGGCGGCCGCCGAGAATGAACGCCAAGAGGAACAAGCATGAAACCGCACCAGGACGGCCAGGACGAACCGTTTTTCATTACCGAAGAGATCGAGGCGGAGATGATCGCGGCCGGGTACGTGTTCGAGCCGCCCGCGCACGTCTCAACCGTGCGGCTGCATGAAATCCTGGCCGGTTTGTCTGATGCCAAGCTGGCGGCCTGGCCGGCCAGCTTGGCCGCTGAAGAAACCGAGCGCCGCCGTCTAAAAAGGTGATGTGTATTTGAGTAAAACAGCTTGCGTCATGCGGTCGCTGCGTATATGATGCGATGAGTAAATAAACAAATACGCAAGGGGAACGCATGAAGGTTATCGCTGTACTTAACCAGAAAGGCGGGTCAGGCAAGACGACCATCGCAACCCATCTAGCCCGCGCCCTGCAACTCGCCGGGGCCGATGTTCTGTTAGTCGATTCCGATCCCCAGGGCAGTGCCCGCGATTGGGCGGCCGTGCGGGAAGATCAACCGCTAACCGTTGTCGGCATCGACCGCCCGACGATTGACCGCGACGTGAAGGCCATCGGCCGGCGCGACTTCGTAGTGATCGACGGAGCGCCCCAGGCGGCGGACTTGGCTGTGTCCGCGATCAAGGCAGCCGACTTCGTGCTGATTCCGGTGCAGCCAAGCCCTTACGACATATGGGCCACCGCCGACCTGGTGGAGCTGGTTAAGCAGCGCATTGAGGTCACGGATGGAAGGCTACAAGCGGCCTTTGTCGTGTCGCGGGCGATCAAAGGCACGCGCATCGGCGGTGAGGTTGCCGAGGCGCTGGCCGGGTACGAGCTGCCCATTCTTGAGTCCCGTATCACGCAGCGCGTGAGCTACCCAGGCACTGCCGCCGCCGGCACAACCGTTCTTGAATCAGAACCCGAGGGCGACGCTGCCCGCGAGGTCCAGGCGCTGGCCGCTGAAATTAAATCAAAACTCATTTGAGTTAATGAGGTAAAGAGAAAATGAGCAAAAGCACAAACACGCTAAGTGCCGGCCGTCCGAGCGCACGCAGCAGCAAGGCTGCAACGTTGGCCAGCCTGGCAGACACGCCAGCCATGAAGCGGGTCAACTTTCAGTTGCCGGCGGAGGATCACACCAAGCTGAAGATGTACGCGGTACGCCAAGGCAAGACCATTACCGAGCTGCTATCTGAATACATCGCGCAGCTACCAGAGTAAATGAGCAAATGAATAAATGAGTAGATGAATTTTAGCGGCTAAAGGAGGCGGCATGGAAAATCAAGAACAACCAGGCACCGACGCCGTGGAATGCCCCATGTGTGGAGGAACGGGCGGTTGGCCAGGCGTAAGCGGCTGGGTTGTCTGCCGGCCCTGCAATGGCACTGGAACCCCCAAGCCCGAGGAATCGGCGTGACGGTCGCAAACCATCCGGCCCGGTACAAATCGGCGCGGCGCTGGGTGATGACCTGGTGGAGAAGTTGAAGGCCGCGCAGGCCGCCCAGCGGCAACGCATCGAGGCAGAAGCACGCCCCGGTGAATCGTGGCAAGCGGCCGCTGATCGAATCCGCAAAGAATCCCGGCAACCGCCGGCAGCCGGTGCGCCGTCGATTAGGAAGCCGCCCAAGGGCGACGAGCAACCAGATTTTTTCGTTCCGATGCTCTATGACGTGGGCACCCGCGATAGTCGCAGCATCATGGACGTGGCCGTTTTCCGTCTGTCGAAGCGTGACCGACGAGCTGGCGAGGTGATCCGCTACGAGCTTCCAGACGGGCACGTAGAGGTTTCCGCAGGGCCGGCCGGCATGGCCAGTGTGTGGGATTACGACCTGGTACTGATGGCGGTTTCCCATCTAACCGAATCCATGAACCGATACCGGGAAGGGAAGGGAGACAAGCCCGGCCGCGTGTTCCGTCCACACGTTGCGGACGTACTCAAGTTCTGCCGGCGAGCCGATGGCGGAAAGCAGAAAGACGACCTGGTAGAAACCTGCATTCGGTTAAACACCACGCACGTTGCCATGCAGCGTACGAAGAAGGCCAAGAACGGCCGCCTGGTGACGGTATCCGAGGGTGAAGCCTTGATTAGCCGCTACAAGATCGTAAAGAGCGAAACCGGGCGGCCGGAGTACATCGAGATCGAGCTAGCTGATTGGATGTACCGCGAGATCACAGAAGGCAAGAACCCGGACGTGCTGACGGTTCACCCCGATTACTTTTTGATCGATCCCGGCATCGGCCGTTTTCTCTACCGCCTGGCACGCCGCGCCGCAGGCAAGGCAGAAGCCAGATGGTTGTTCAAGACGATCTACGAACGCAGTGGCAGCGCCGGAGAGTTCAAGAAGTTCTGTTTCACCGTGCGCAAGCTGATCGGGTCAAATGACCTGCCGGAGTACGATTTGAAGGAGGAGGCGGGGCAGGCTGGCCCGATCCTAGTCATGCGCTACCGCAACCTGATCGAGGGCGAAGCATCCGCCGGTTCCTAATGTACGGAGCAGATGCTAGGGCAAATTGCCCTAGCAGGGGAAAAAGGTCGAAAAGGTCTCTTTCCTGTGGATAGCACGTACATTGGGAACCCAAAGCCGTACATTGGGAACCGGAACCCGTACATTGGGAACCCAAAGCCGTACATTGGGAACCGGTCACACATGTAAGTGACTGATATAAAAGAGAAAAAAGGCGATTTTTCCGCCTAAAACTCTTTAAAACTTATTAAAACTCTTAAAACCCGCCTGGCCTGTGCATAACTGTCTGGCCAGCGCACAGCCGAAGAGCTGCAAAAAGCGCCTACCCTTCGGTCGCTGCGCTCCCTACGCCCCGCCGCTTCGCGTCGGCCTATCGCGGCCGCTGGCCGCTCAAAAATGGCTGGCCTACGGCCAGGCAATCTACCAGGGCGCGGACAAGCCGCGCCGTCGCCACTCGACCGCCGGCGCCCACATCAAGGCACCCTGCCTCGCGCGTTTCGGTGATGACGGTGAAAACCTCTGACACATGCAGCTCCCGGAGACGGTCACAGCTTGTCTGTAAGCGGATGCCGGGAGCAGACAAGCCCGTCAGGGCGCGTCAGCGGGTGTTGGCGGGTGTCGGGGCGCAGCCATGACCCAGTCACGTAGCGATAGCGGAGTGTATACTGGCTTAACTATGCGGCATCAGAGCAGATTGTACTGAGAGTGCACCATATGCGGTGTGAAATACCGCACAGATGCGTAAGGAGAAAATACCGCATCAGGCGCTCTTCCGCTTCCTCGCTCACTGACTCGCTGCGCTCGGTCGTTCGGCTGCGGCGAGCGGTATCAGCTCACTCAAAGGCGGTAATACGGTTATCCACAGAATCAGGGGATAACGCAGGAAAGAACATGTGAGCAAAAGGCCAGCAAAAGGCCAGGAACCGTAAAAAGGCCGCGTTGCTGGCGTTTTTCCATAGGCTCCGCCCCCCTGACGAGCATCACAAAAATCGACGCTCAAGTCAGAGGTGGCGAAACCCGACAGGACTATAAAGATACCAGGCGTTTCCCCCTGGAAGCTCCCTCGTGCGCTCTCCTGTTCCGACCCTGCCGCTTACCGGATACCTGTCCGCCTTTCTCCCTTCGGGAAGCGTGGCGCTTTCTCATAGCTCACGCTGTAGGTATCTCAGTTCGGTGTAGGTCGTTCGCTCCAAGCTGGGCTGTGTGCACGAACCCCCCGTTCAGCCCGACCGCTGCGCCTTATCCGGTAACTATCGTCTTGAGTCCAACCCGGTAAGACACGACTTATCGCCACTGGCAGCAGCCACTGGTAACAGGATTAGCAGAGCGAGGTATGTAGGCGGTGCTACAGAGTTCTTGAAGTGGTGGCCTAACTACGGCTACACTAGAAGGACAGTATTTGGTATCTGCGCTCTGCTGAAGCCAGTTACCTTCGGAAAAAGAGTTGGTAGCTCTTGATCCGGCAAACAAACCACCGCTGGTAGCGGTGGTTTTTTTGTTTGCAAGCAGCAGATTACGCGCAGAAAAAAAGGATCTCAAGAAGATCCTTTGATCTTTTCTACGGGGTCTGACGCTCAGTGGAACGAAAACTCACGTTAAGGGATTTTGGTCATGCATTCTAGGTACTAAAACAATTCATCCAGTAAAATATAATATTTTATTTTCTCCCAATCAGGCTTGATCCCCAGTAAGTCAAAAAATAGCTCGACATACTGTTCTTCCCCGATATCCTCCCTGATCGACCGGACGCAGAAGGCAATGTCATACCACTTGTCCGCCCTGCCGCTTCTCCCAAGATCAATAAAGCCACTTACTTTGCCATCTTTCACAAAGATGTTGCTGTCTCCCAGGTCGCCGTGGGAAAAGACAAGTTCCTCTTCGGGCTTTTCCGTCTTTAAAAAATCATACAGCTCGCGCGGATCTTTAAATGGAGTGTCTTCTTCCCAGTTTTCGCAATCCACATCGGCCAGATCGTTATTCAGTAAGTAATCCAATTCGGCTAAGCGGCTGTCTAAGCTATTCGTATAGGGACAATCCGATATGTCGATGGAGTGAAAGAGCCTGATGCACTCCGCATACAGCTCGATAATCTTTTCAGGGCTTTGTTCATCTTCATACTCTTCCGAGCAAAGGACGCCATCGGCCTCACTCATGAGCAGATTGCTCCAGCCATCATGCCGTTCAAAGTGCAGGACCTTTGGAACAGGCAGCTTTCCTTCCAGCCATAGCATCATGTCCTTTTCCCGTTCCACATCATAGGTGGTCCCTTTATACCGGCTGTCCGTCATTTTTAAATATAGGTTTTCATTTTCTCCCACCAGCTTATATACCTTAGCAGGAGACATTCCTTCCGTATCTTTTACGCAGCGGTATTTTTCGATCAGTTTTTTCAATTCCGGTGATATTCTCATTTTAGCCATTTATTATTTCCTTCCTCTTTTCTACAGTATTTAAAGATACCCCAAGAAGCTAATTATAACAAGACGAACTCCAATTCACTGTTCCTTGCATTCTAAAACCTTAAATACCAGAAAACAGCTTTTTCAAAGTTGTTTTCAAAGTTGGCGTATAACATAGTATCGACGGAGCCGATTTTGAAACCGCGGTGATCACAGGCAGCAACGCTCTGTCATCGTTACAATCAACATGCTACCCTCCGCGAGATCATCCGTGTTTCAAACCCGGCAGCTTAGTTGCCGTTCTTCCGAATAGCATCGGTAACATGAGCAAAGTCTGCCGCCTTACAACGGCTCTCCCGCTGACGCCGTCCCGGACTGATGGGCTGCCTGTATCGAGTGGTGATTTTGTGCCGAGCTGCCGGTCGGGGAGCTGTTGGCTGGCTGGTGGCAGGATATATTGTGGTGTAAACAAATTGACGCTTAGACAACTTAATAACACATTGCGGACGTTTTTAATGTACTGAATTAACGCCGAATTAATTCGGGGGATCTGGATTTTAGTACTGGATTTTGGTTTTAGGAATTAGAAATTTTATTGATAGAAGTATTTTACAAATACAAATACATACTAAGGGTTTCTTATATGCTCAACACATGAGCGAAACCCTATAGGAACCCTAATTCCCTTATCTGGGAACTACTCACACATTATTATGGAGAAACTCGAGCTTGTCGATCGACAGATCCTACAGGAACAGGTGGTGGCGGCCCTCGGTGCGCTCGTACTGCTCCACGATGGTGTAGTCCTCGTTGTGGGAGGTGATGTCCAGCTTGGCGTCCACGTAGTAGTAGCCGGGCAGCTGCACGGGCTTCTTGGCCATGTAGATGGACTTGAACTCCACCAGGTAGTGGCCGCCGTCCTTCAGCTTCAGGGCCTTGTGGGTCTCGCCCTTCAGCACGCCGTCGCGGGGGTACAGGCGCTCGGTGGAGGCCTCCCAGCCCATGGTCTTCTTCTGCATCACGGGGCCGTCGGAGGGGAAGTTCACGCCGATGAACTTCACCTTGTAGATGAAGCAGCCGTCCTGCAGGGAGGAGTCCTGGGTCACGGTCGCCACGCCGCCGTCCTCGAAGTTCATCACGCGCTCCCACTTGAAGCCCTCGGGGAAGGACAGCTTCTTGTAGTCGGGGATGTCGGCGGGGTGCTTCACGTACACCTTGGAGCCGTACTGGAACTGGGGGGACAGGATGTCCCAGGCGAAGGGCAGGGGGCCGCCCTTGGTCACCTTCAGCTTCACGGTGTTGTGGCCCTCGTAGGGGCGGCCCTCGCCCTCGCCCTCGATCTCGAACTCGTGGCCGTTCACGGTGCCCTCCATGCGCACCTTGAAGCGCATGAACTCGGTGATGACGTTCTCGGAGGAGGCCATGGTTTCGATCCACTTTCTTACAAATTTCTCTGAAGTTGTATCCTCAGTACTTCAAAGAAAATAGCTTACACCAATTTTTTCTTGTTTTCACAAATGCCGAACTTGGTTCCTTATATAGGAAAACTCAAGGGCAAAAATGACACGGAAAAATATAAAAGGATAAGTAGTGGGGGATAAGATTCCTTTGTGATAAGGTTACTTTCCGCCCTTACATTTTCCACCTTACATGTGTCCTCTATGTCTCTTTCACAATCACCGACCTTATCTTCTTCTTTTCATTGTTGTCGTCAGTGCTTACGTCTTCAAGATTCTTTTCTTCGCCTGGTTCTTCTTTTTCAATTTCTACGTATTCTTCTTCGTATTCTGGCAGTATAGGATCTTGTATCTGTACATTCTTCATTTTTGAACATAGGTTGCATATGTGCCGCATATTGATCTGCTTCTTGCTGAGCTCACATAATACTTCCATAGTTTTTCCCGTAAACATTGGATTCTTGATGCTACATCTTGGATAATTACCTTCTACTAGTCG

**DNA sequence of binary vector containing Cas9/sgRNA genes targeting both the Arabidopsis and the Camelina FAD2 R2 site, *BbvCI***

AATTCCATGGAGTCAAAGATTCAAATAGAGGACCTAACAGAACTCGCCGTAAAGACTGGCGAACAGTTCATACAGAGTCTCTTACGACTCAATGACAAGAAGAAAATCTTCGTCAACATGGTGGAGCACGACACACTTGTCTACTCCAAAAATATCAAAGATACAGTCTCAGAAGACCAAAGGGCAATTGAGACTTTTCAACAAAGGGTAATATCCGGAAACCTCCTCGGATTCCATTGCCCAGCTATCTGTCACTTTATTGTGAAGATAGTGGAAAAGGAAGGTGGCTCCTACAAATGCCATCATTGCGATAAAGGAAAGGCCATCGTTGAAGATGCCTCTGCCGACAGTGGTCCCAAAGATGGACCCCCACCCACGAGGAGCATCGTGGAAAAAGAAGACGTTCCAACCACGTCTTCAAAGCAAGTGGATTGATGTGATATCTCCACTGACGTAAGGGATGACGCACAATCCCACTATCCTTCGCAAGACCCTTCCTCTATATAAGGAAGTTCATTTCATTTGGAGAGAACACGGGGGACTCTTGACCATGGTAGATCTGACTAGTGATTACAAGGACGATGATGACAAGAAAGACTATAAAGATGACGATGATAAGCATATGGACAAGAAGTACAGCATCGGCCTGGACATCGGCACGAACTCGGTGGGCTGGGCGGTGATCACGGACGAGTACAAGGTGCCCTCCAAGAAGTTCAAGGTGCTGGGCAACACCGACCGCCACTCGATCAAGAAGAACCTGATCGGCGCCCTGCTGTTCGACTCCGGCGAGACCGCCGAGGCGACGCGCCTGAAGCGCACCGCGCGTCGCCGCTACACGCGTCGCAAGAACCGCATCTGCTACCTGCAGGAGATCTTCAGCAACGAGATGGCCAAGGTGGACGACTCGTTCTTCCACCGCCTGGAGGAGTCCTTCCTGGTGGAGGAAGACAAGAAGCACGAGCGCCACCCCATCTTCGGCAACATCGTGGACGAGGTGGCCTACCACGAGAAGTACCCGACGATCTACCACCTGCGCAAGAAGCTGGTGGACAGCACCGACAAGGCGGACCTGCGCCTGATCTACCTGGCCCTGGCGCACATGATCAAGTTCCGCGGCCACTTCCTGATCGAGGGCGACCTGAACCCCGACAACTCGGACGTGGACAAGCTGTTCATCCAGCTGGTGCAGACCTACAACCAGCTGTTCGAGGAGAACCCGATCAACGCCTCCGGCGTGGACGCCAAGGCGATCCTGAGCGCGCGCCTGTCCAAGAGCCGTCGCCTGGAGAACCTGATCGCCCAGCTGCCCGGCGAGAAGAAGAACGGCCTGTTCGGCAACCTGATCGCGCTGTCGCTGGGCCTGACGCCGAACTTCAAGTCCAACTTCGACCTGGCCGAGGACGCGAAGCTGCAGCTGAGCAAGGACACCTACGACGACGACCTGGACAACCTGCTGGCCCAGATCGGCGACCAGTACGCGGACCTGTTCCTGGCCGCGAAGAACCTGTCGGACGCCATCCTGCTGTCCGACATCCTGCGCGTGAACACCGAGATCACGAAGGCCCCCCTGTCGGCGTCCATGATCAAGCGCTACGACGAGCACCACCAGGACCTGACCCTGCTGAAGGCGCTGGTGCGCCAGCAGCTGCCGGAGAAGTACAAGGAGATCTTCTTCGACCAGAGCAAGAACGGCTACGCCGGCTACATCGACGGCGGCGCGTCGCAAGAGGAGTTCTACAAGTTCATCAAGCCCATCCTGGAGAAGATGGACGGCACGGAGGAGCTGCTGGTGAAGCTGAACCGCGAGGACCTGCTGCGCAAGCAGCGCACCTTCGACAACGGCAGCATCCCCCACCAGATCCACCTGGGCGAGCTGCACGCCATCCTGCGTCGCCAAGAGGACTTCTACCCGTTCCTGAAGGACAACCGCGAGAAGATCGAGAAGATCCTGACGTTCCGCATCCCCTACTACGTGGGCCCGCTGGCCCGCGGCAACAGCCGCTTCGCGTGGATGACCCGCAAGTCGGAGGAGACCATCACGCCCTGGAACTTCGAGGAAGTGGTGGACAAGGGCGCCAGCGCGCAGTCGTTCATCGAGCGCATGACCAACTTCGACAAGAACCTGCCCAACGAGAAGGTGCTGCCGAAGCACTCCCTGCTGTACGAGTACTTCACCGTGTACAACGAGCTGACGAAGGTGAAGTACGTGACCGAGGGCATGCGCAAGCCCGCCTTCCTGAGCGGCGAGCAGAAGAAGGCGATCGTGGACCTGCTGTTCAAGACCAACCGCAAGGTGACGGTGAAGCAGCTGAAAGAGGACTACTTCAAGAAGATCGAGTGCTTCGACAGCGTGGAGATCTCGGGCGTGGAGGACCGCTTCAACGCCAGCCTGGGCACCTACCACGACCTGCTGAAGATCATCAAGGACAAGGACTTCCTGGACAACGAGGAGAACGAGGACATCCTGGAGGACATCGTGCTGACCCTGACGCTGTTCGAGGACCGCGAGATGATCGAGGAGCGCCTGAAGACGTACGCCCACCTGTTCGACGACAAGGTGATGAAGCAGCTGAAGCGTCGCCGCTACACCGGCTGGGGCCGCCTGAGCCGCAAGCTGATCAACGGCATCCGCGACAAGCAGTCCGGCAAGACCATCCTGGACTTCCTGAAGAGCGACGGCTTCGCGAACCGCAACTTCATGCAGCTGATCCACGACGACTCGCTGACCTTCAAAGAGGACATCCAGAAGGCCCAGGTGTCGGGCCAGGGCGACTCCCTGCACGAGCACATCGCCAACCTGGCGGGCTCCCCCGCGATCAAGAAGGGCATCCTGCAGACCGTGAAGGTGGTGGACGAGCTGGTGAAGGTGATGGGCCGCCACAAGCCGGAGAACATCGTGATCGAGATGGCCCGCGAGAACCAGACCACGCAGAAGGGCCAGAAGAACAGCCGCGAGCGCATGAAGCGCATCGAGGAAGGCATCAAGGAGCTGGGCTCGCAGATCCTGAAGGAGCACCCCGTGGAGAACACCCAGCTGCAGAACGAGAAGCTGTACCTGTACTACCTGCAGAACGGCCGCGACATGTACGTGGACCAGGAGCTGGACATCAACCGCCTGTCCGACTACGACGTGGACCACATCGTGCCCCAGAGCTTCCTGAAGGACGACTCGATCGACAACAAGGTGCTGACCCGCAGCGACAAGAACCGCGGCAAGAGCGACAACGTGCCGTCGGAGGAAGTGGTGAAGAAGATGAAGAACTACTGGCGCCAGCTGCTGAACGCCAAGCTGATCACGCAGCGCAAGTTCGACAACCTGACCAAGGCCGAGCGCGGTGGCCTGTCGGAGCTGGACAAGGCGGGCTTCATCAAGCGCCAGCTGGTGGAGACCCGCCAGATCACGAAGCACGTGGCGCAGATCCTGGACTCCCGCATGAACACGAAGTACGACGAGAACGACAAGCTGATCCGCGAGGTGAAGGTGATCACCCTGAAGTCCAAGCTGGTCAGCGACTTCCGCAAGGACTTCCAGTTCTACAAGGTGCGCGAGATCAACAACTACCACCACGCCCACGACGCGTACCTGAACGCCGTGGTGGGCACCGCGCTGATCAAGAAGTACCCCAAGCTGGAGAGCGAGTTCGTGTACGGCGACTACAAGGTGTACGACGTGCGCAAGATGATCGCCAAGTCGGAGCAGGAGATCGGCAAGGCCACCGCGAAGTACTTCTTCTACTCCAACATCATGAACTTCTTCAAGACCGAGATCACGCTGGCCAACGGCGAGATCCGCAAGCGCCCGCTGATCGAGACCAACGGCGAGACGGGCGAGATCGTGTGGGACAAGGGCCGCGACTTCGCGACCGTGCGCAAGGTGCTGAGCATGCCCCAGGTGAACATCGTGAAGAAGACCGAGGTGCAGACGGGCGGCTTCTCCAAGGAGAGCATCCTGCCGAAGCGCAACTCGGACAAGCTGATCGCCCGCAAGAAGGACTGGGACCCCAAGAAGTACGGCGGCTTCGACTCCCCGACCGTGGCCTACAGCGTGCTGGTGGTGGCGAAGGTGGAGAAGGGCAAGTCCAAGAAGCTGAAGAGCGTGAAGGAGCTGCTGGGCATCACCATCATGGAGCGCAGCTCGTTCGAGAAGAACCCCATCGACTTCCTGGAGGCCAAGGGCTACAAAGAGGTGAAGAAGGACCTGATCATCAAGCTGCCGAAGTACTCGCTGTTCGAGCTGGAGAACGGCCGCAAGCGCATGCTGGCCTCCGCGGGCGAGCTGCAGAAGGGCAACGAGCTGGCCCTGCCCAGCAAGTACGTGAACTTCCTGTACCTGGCGTCCCACTACGAGAAGCTGAAGGGCTCGCCGGAGGACAACGAGCAGAAGCAGCTGTTCGTGGAGCAGCACAAGCACTACCTGGACGAGATCATCGAGCAGATCTCGGAGTTCTCCAAGCGCGTGATCCTGGCCGACGCGAACCTGGACAAGGTGCTGAGCGCCTACAACAAGCACCGCGACAAGCCCATCCGCGAGCAGGCGGAGAACATCATCCACCTGTTCACCCTGACGAACCTGGGCGCCCCGGCCGCGTTCAAGTACTTCGACACCACGATCGACCGCAAGCGCTACACCTCCACGAAAGAGGTGCTGGACGCGACCCTGATCCACCAGAGCATCACCGGCCTGTACGAGACGCGCATCGACCTGAGCCAGCTGGGCGGCGACTCCCGCGCGGACCCGAAGAAGAAGCGCAAGGTGTAAGAATTAATTCGGATCGATCCATTGGTGACCAGCTCGAATTTCCCCGATCGTTCAAACATTTGGCAATAAAGTTTCTTAAGATTGAATCCTGTTGCCGGTCTTGCGATGATTATCATATAATTTCTGTTGAATTACGTTAAGCATGTAATAATTAACATGTAATGCATGACGTTATTTATGAGATGGGTTTTTATGATTAGAGTCCCGCAATTATACATTTAATACGCGATAGAAAACAAAATATAGCGCGCAAACTAGGATAAATTATCGCGCGCGGTGTCATCTATGTTACTAGATCGGGGGTACCCGTTGAACAACGGAAACTCGACTTGCCTTCCGCACAATACATCATTTCTTCTTAGCTTTTTTTCTTCTTCTTCGTTCATACAGTTTTTTTTTGTTTATCAGCTTACATTTTCTTGAACCGTAGCTTTCGTTTTCTTCTTTTTAACTTTCCATTCGGAGTTTTTGTATCTTGTTTCATAGTTTGTCCCAGGATTAGAATGATTAGGCATCGAACCTTCAAGAATTTGATTGAATAAAACATCTTCATTCTTAAGATATGAAGATAATCTTCAAAAGGCCCCTGGGAATCTGAAAGAAGAGAAGCAGGCCCATTTATATGGGAAAGAACAATAGTATTTCTTATATAGGCCCATTTAAGTTGAAAACAATCTTCAAAAGTCCCACATCGCTTAGATAAGAAAACGAAGCTGAGTTTATATACAGCTAGAGTCGAAGTAGTGATTGTAAGAGAGAGGCTGAGGGGTTTTAGAGCTAGAAATAGCAAGTTAAAATAAGGCTAGTCCGTTATCAACTTGAAAAAGTGGCACCGAGTCGGTGCTTTTTTTTTTTGCAAAATTTTCCAGATCGATTTCTTCTTCCTCTGTTCTTCGGCGTTCAATTTCTGGGTTTTTCTCTTCGTTTTCTGTAACTGAAACCTAAAATTTGACCTAAAAAAAATCTCAAATAATATGATTCAGTGGTTTTGTACTTTTCAGTTAGTTGAGTTTTGCAGTTCCGATGAGATAAACCAATAACTTTGCTTAGATCTAATTCATTCCGTTACACCTCTGATGGAGATGGAAGGTTCTTAATAATGATGCCATTTTTTGGGTAATAATTTTGAATTAGAATCAAGGGTATAAGATTCATAATTAACATCACTTAAGCAAAGTTCGTAATATACGACCACAGGATATAATTTTTGGTCTAGAGTCGACCTGCAGGCATGCAAGCTTGGCACTGGCCGTCGTTTTACAACGTCGTGACTGGGAAAACCCTGGCGTTACCCAACTTAATCGCCTTGCAGCACATCCCCCTTTCGCCAGCTGGCGTAATAGCGAAGAGGCCCGCACCGATCGCCCTTCCCAACAGTTGCGCAGCCTGAATGGCGAATGCTAGAGCAGCTTGAGCTTGGATCAGATTGTCGTTTCCCGCCTTCAGTTTAAACTATCAGTGTTTGACAGGATATATTGGCGGGTAAACCTAAGAGAAAAGAGCGTTTATTAGAATAACGGATATTTAAAAGGGCGTGAAAAGGTTTATCCGTTCGTCCATTTGTATGTGCATGCCAACCACAGGGTTCCCCTCGGGATCAAAGTACTTTGATCCAACCCCTCCGCTGCTATAGTGCAGTCGGCTTCTGACGTTCAGTGCAGCCGTCTTCTGAAAACGACATGTCGCACAAGTCCTAAGTTACGCGACAGGCTGCCGCCCTGCCCTTTTCCTGGCGTTTTCTTGTCGCGTGTTTTAGTCGCATAAAGTAGAATACTTGCGACTAGAACCGGAGACATTACGCCATGAACAAGAGCGCCGCCGCTGGCCTGCTGGGCTATGCCCGCGTCAGCACCGACGACCAGGACTTGACCAACCAACGGGCCGAACTGCACGCGGCCGGCTGCACCAAGCTGTTTTCCGAGAAGATCACCGGCACCAGGCGCGACCGCCCGGAGCTGGCCAGGATGCTTGACCACCTACGCCCTGGCGACGTTGTGACAGTGACCAGGCTAGACCGCCTGGCCCGCAGCACCCGCGACCTACTGGACATTGCCGAGCGCATCCAGGAGGCCGGCGCGGGCCTGCGTAGCCTGGCAGAGCCGTGGGCCGACACCACCACGCCGGCCGGCCGCATGGTGTTGACCGTGTTCGCCGGCATTGCCGAGTTCGAGCGTTCCCTAATCATCGACCGCACCCGGAGCGGGCGCGAGGCCGCCAAGGCCCGAGGCGTGAAGTTTGGCCCCCGCCCTACCCTCACCCCGGCACAGATCGCGCACGCCCGCGAGCTGATCGACCAGGAAGGCCGCACCGTGAAAGAGGCGGCTGCACTGCTTGGCGTGCATCGCTCGACCCTGTACCGCGCACTTGAGCGCAGCGAGGAAGTGACGCCCACCGAGGCCAGGCGGCGCGGTGCCTTCCGTGAGGACGCATTGACCGAGGCCGACGCCCTGGCGGCCGCCGAGAATGAACGCCAAGAGGAACAAGCATGAAACCGCACCAGGACGGCCAGGACGAACCGTTTTTCATTACCGAAGAGATCGAGGCGGAGATGATCGCGGCCGGGTACGTGTTCGAGCCGCCCGCGCACGTCTCAACCGTGCGGCTGCATGAAATCCTGGCCGGTTTGTCTGATGCCAAGCTGGCGGCCTGGCCGGCCAGCTTGGCCGCTGAAGAAACCGAGCGCCGCCGTCTAAAAAGGTGATGTGTATTTGAGTAAAACAGCTTGCGTCATGCGGTCGCTGCGTATATGATGCGATGAGTAAATAAACAAATACGCAAGGGGAACGCATGAAGGTTATCGCTGTACTTAACCAGAAAGGCGGGTCAGGCAAGACGACCATCGCAACCCATCTAGCCCGCGCCCTGCAACTCGCCGGGGCCGATGTTCTGTTAGTCGATTCCGATCCCCAGGGCAGTGCCCGCGATTGGGCGGCCGTGCGGGAAGATCAACCGCTAACCGTTGTCGGCATCGACCGCCCGACGATTGACCGCGACGTGAAGGCCATCGGCCGGCGCGACTTCGTAGTGATCGACGGAGCGCCCCAGGCGGCGGACTTGGCTGTGTCCGCGATCAAGGCAGCCGACTTCGTGCTGATTCCGGTGCAGCCAAGCCCTTACGACATATGGGCCACCGCCGACCTGGTGGAGCTGGTTAAGCAGCGCATTGAGGTCACGGATGGAAGGCTACAAGCGGCCTTTGTCGTGTCGCGGGCGATCAAAGGCACGCGCATCGGCGGTGAGGTTGCCGAGGCGCTGGCCGGGTACGAGCTGCCCATTCTTGAGTCCCGTATCACGCAGCGCGTGAGCTACCCAGGCACTGCCGCCGCCGGCACAACCGTTCTTGAATCAGAACCCGAGGGCGACGCTGCCCGCGAGGTCCAGGCGCTGGCCGCTGAAATTAAATCAAAACTCATTTGAGTTAATGAGGTAAAGAGAAAATGAGCAAAAGCACAAACACGCTAAGTGCCGGCCGTCCGAGCGCACGCAGCAGCAAGGCTGCAACGTTGGCCAGCCTGGCAGACACGCCAGCCATGAAGCGGGTCAACTTTCAGTTGCCGGCGGAGGATCACACCAAGCTGAAGATGTACGCGGTACGCCAAGGCAAGACCATTACCGAGCTGCTATCTGAATACATCGCGCAGCTACCAGAGTAAATGAGCAAATGAATAAATGAGTAGATGAATTTTAGCGGCTAAAGGAGGCGGCATGGAAAATCAAGAACAACCAGGCACCGACGCCGTGGAATGCCCCATGTGTGGAGGAACGGGCGGTTGGCCAGGCGTAAGCGGCTGGGTTGTCTGCCGGCCCTGCAATGGCACTGGAACCCCCAAGCCCGAGGAATCGGCGTGACGGTCGCAAACCATCCGGCCCGGTACAAATCGGCGCGGCGCTGGGTGATGACCTGGTGGAGAAGTTGAAGGCCGCGCAGGCCGCCCAGCGGCAACGCATCGAGGCAGAAGCACGCCCCGGTGAATCGTGGCAAGCGGCCGCTGATCGAATCCGCAAAGAATCCCGGCAACCGCCGGCAGCCGGTGCGCCGTCGATTAGGAAGCCGCCCAAGGGCGACGAGCAACCAGATTTTTTCGTTCCGATGCTCTATGACGTGGGCACCCGCGATAGTCGCAGCATCATGGACGTGGCCGTTTTCCGTCTGTCGAAGCGTGACCGACGAGCTGGCGAGGTGATCCGCTACGAGCTTCCAGACGGGCACGTAGAGGTTTCCGCAGGGCCGGCCGGCATGGCCAGTGTGTGGGATTACGACCTGGTACTGATGGCGGTTTCCCATCTAACCGAATCCATGAACCGATACCGGGAAGGGAAGGGAGACAAGCCCGGCCGCGTGTTCCGTCCACACGTTGCGGACGTACTCAAGTTCTGCCGGCGAGCCGATGGCGGAAAGCAGAAAGACGACCTGGTAGAAACCTGCATTCGGTTAAACACCACGCACGTTGCCATGCAGCGTACGAAGAAGGCCAAGAACGGCCGCCTGGTGACGGTATCCGAGGGTGAAGCCTTGATTAGCCGCTACAAGATCGTAAAGAGCGAAACCGGGCGGCCGGAGTACATCGAGATCGAGCTAGCTGATTGGATGTACCGCGAGATCACAGAAGGCAAGAACCCGGACGTGCTGACGGTTCACCCCGATTACTTTTTGATCGATCCCGGCATCGGCCGTTTTCTCTACCGCCTGGCACGCCGCGCCGCAGGCAAGGCAGAAGCCAGATGGTTGTTCAAGACGATCTACGAACGCAGTGGCAGCGCCGGAGAGTTCAAGAAGTTCTGTTTCACCGTGCGCAAGCTGATCGGGTCAAATGACCTGCCGGAGTACGATTTGAAGGAGGAGGCGGGGCAGGCTGGCCCGATCCTAGTCATGCGCTACCGCAACCTGATCGAGGGCGAAGCATCCGCCGGTTCCTAATGTACGGAGCAGATGCTAGGGCAAATTGCCCTAGCAGGGGAAAAAGGTCGAAAAGGTCTCTTTCCTGTGGATAGCACGTACATTGGGAACCCAAAGCCGTACATTGGGAACCGGAACCCGTACATTGGGAACCCAAAGCCGTACATTGGGAACCGGTCACACATGTAAGTGACTGATATAAAAGAGAAAAAAGGCGATTTTTCCGCCTAAAACTCTTTAAAACTTATTAAAACTCTTAAAACCCGCCTGGCCTGTGCATAACTGTCTGGCCAGCGCACAGCCGAAGAGCTGCAAAAAGCGCCTACCCTTCGGTCGCTGCGCTCCCTACGCCCCGCCGCTTCGCGTCGGCCTATCGCGGCCGCTGGCCGCTCAAAAATGGCTGGCCTACGGCCAGGCAATCTACCAGGGCGCGGACAAGCCGCGCCGTCGCCACTCGACCGCCGGCGCCCACATCAAGGCACCCTGCCTCGCGCGTTTCGGTGATGACGGTGAAAACCTCTGACACATGCAGCTCCCGGAGACGGTCACAGCTTGTCTGTAAGCGGATGCCGGGAGCAGACAAGCCCGTCAGGGCGCGTCAGCGGGTGTTGGCGGGTGTCGGGGCGCAGCCATGACCCAGTCACGTAGCGATAGCGGAGTGTATACTGGCTTAACTATGCGGCATCAGAGCAGATTGTACTGAGAGTGCACCATATGCGGTGTGAAATACCGCACAGATGCGTAAGGAGAAAATACCGCATCAGGCGCTCTTCCGCTTCCTCGCTCACTGACTCGCTGCGCTCGGTCGTTCGGCTGCGGCGAGCGGTATCAGCTCACTCAAAGGCGGTAATACGGTTATCCACAGAATCAGGGGATAACGCAGGAAAGAACATGTGAGCAAAAGGCCAGCAAAAGGCCAGGAACCGTAAAAAGGCCGCGTTGCTGGCGTTTTTCCATAGGCTCCGCCCCCCTGACGAGCATCACAAAAATCGACGCTCAAGTCAGAGGTGGCGAAACCCGACAGGACTATAAAGATACCAGGCGTTTCCCCCTGGAAGCTCCCTCGTGCGCTCTCCTGTTCCGACCCTGCCGCTTACCGGATACCTGTCCGCCTTTCTCCCTTCGGGAAGCGTGGCGCTTTCTCATAGCTCACGCTGTAGGTATCTCAGTTCGGTGTAGGTCGTTCGCTCCAAGCTGGGCTGTGTGCACGAACCCCCCGTTCAGCCCGACCGCTGCGCCTTATCCGGTAACTATCGTCTTGAGTCCAACCCGGTAAGACACGACTTATCGCCACTGGCAGCAGCCACTGGTAACAGGATTAGCAGAGCGAGGTATGTAGGCGGTGCTACAGAGTTCTTGAAGTGGTGGCCTAACTACGGCTACACTAGAAGGACAGTATTTGGTATCTGCGCTCTGCTGAAGCCAGTTACCTTCGGAAAAAGAGTTGGTAGCTCTTGATCCGGCAAACAAACCACCGCTGGTAGCGGTGGTTTTTTTGTTTGCAAGCAGCAGATTACGCGCAGAAAAAAAGGATCTCAAGAAGATCCTTTGATCTTTTCTACGGGGTCTGACGCTCAGTGGAACGAAAACTCACGTTAAGGGATTTTGGTCATGCATTCTAGGTACTAAAACAATTCATCCAGTAAAATATAATATTTTATTTTCTCCCAATCAGGCTTGATCCCCAGTAAGTCAAAAAATAGCTCGACATACTGTTCTTCCCCGATATCCTCCCTGATCGACCGGACGCAGAAGGCAATGTCATACCACTTGTCCGCCCTGCCGCTTCTCCCAAGATCAATAAAGCCACTTACTTTGCCATCTTTCACAAAGATGTTGCTGTCTCCCAGGTCGCCGTGGGAAAAGACAAGTTCCTCTTCGGGCTTTTCCGTCTTTAAAAAATCATACAGCTCGCGCGGATCTTTAAATGGAGTGTCTTCTTCCCAGTTTTCGCAATCCACATCGGCCAGATCGTTATTCAGTAAGTAATCCAATTCGGCTAAGCGGCTGTCTAAGCTATTCGTATAGGGACAATCCGATATGTCGATGGAGTGAAAGAGCCTGATGCACTCCGCATACAGCTCGATAATCTTTTCAGGGCTTTGTTCATCTTCATACTCTTCCGAGCAAAGGACGCCATCGGCCTCACTCATGAGCAGATTGCTCCAGCCATCATGCCGTTCAAAGTGCAGGACCTTTGGAACAGGCAGCTTTCCTTCCAGCCATAGCATCATGTCCTTTTCCCGTTCCACATCATAGGTGGTCCCTTTATACCGGCTGTCCGTCATTTTTAAATATAGGTTTTCATTTTCTCCCACCAGCTTATATACCTTAGCAGGAGACATTCCTTCCGTATCTTTTACGCAGCGGTATTTTTCGATCAGTTTTTTCAATTCCGGTGATATTCTCATTTTAGCCATTTATTATTTCCTTCCTCTTTTCTACAGTATTTAAAGATACCCCAAGAAGCTAATTATAACAAGACGAACTCCAATTCACTGTTCCTTGCATTCTAAAACCTTAAATACCAGAAAACAGCTTTTTCAAAGTTGTTTTCAAAGTTGGCGTATAACATAGTATCGACGGAGCCGATTTTGAAACCGCGGTGATCACAGGCAGCAACGCTCTGTCATCGTTACAATCAACATGCTACCCTCCGCGAGATCATCCGTGTTTCAAACCCGGCAGCTTAGTTGCCGTTCTTCCGAATAGCATCGGTAACATGAGCAAAGTCTGCCGCCTTACAACGGCTCTCCCGCTGACGCCGTCCCGGACTGATGGGCTGCCTGTATCGAGTGGTGATTTTGTGCCGAGCTGCCGGTCGGGGAGCTGTTGGCTGGCTGGTGGCAGGATATATTGTGGTGTAAACAAATTGACGCTTAGACAACTTAATAACACATTGCGGACGTTTTTAATGTACTGAATTAACGCCGAATTAATTCGGGGGATCTGGATTTTAGTACTGGATTTTGGTTTTAGGAATTAGAAATTTTATTGATAGAAGTATTTTACAAATACAAATACATACTAAGGGTTTCTTATATGCTCAACACATGAGCGAAACCCTATAGGAACCCTAATTCCCTTATCTGGGAACTACTCACACATTATTATGGAGAAACTCGAGCTTGTCGATCGACAGATCCTACAGGAACAGGTGGTGGCGGCCCTCGGTGCGCTCGTACTGCTCCACGATGGTGTAGTCCTCGTTGTGGGAGGTGATGTCCAGCTTGGCGTCCACGTAGTAGTAGCCGGGCAGCTGCACGGGCTTCTTGGCCATGTAGATGGACTTGAACTCCACCAGGTAGTGGCCGCCGTCCTTCAGCTTCAGGGCCTTGTGGGTCTCGCCCTTCAGCACGCCGTCGCGGGGGTACAGGCGCTCGGTGGAGGCCTCCCAGCCCATGGTCTTCTTCTGCATCACGGGGCCGTCGGAGGGGAAGTTCACGCCGATGAACTTCACCTTGTAGATGAAGCAGCCGTCCTGCAGGGAGGAGTCCTGGGTCACGGTCGCCACGCCGCCGTCCTCGAAGTTCATCACGCGCTCCCACTTGAAGCCCTCGGGGAAGGACAGCTTCTTGTAGTCGGGGATGTCGGCGGGGTGCTTCACGTACACCTTGGAGCCGTACTGGAACTGGGGGGACAGGATGTCCCAGGCGAAGGGCAGGGGGCCGCCCTTGGTCACCTTCAGCTTCACGGTGTTGTGGCCCTCGTAGGGGCGGCCCTCGCCCTCGCCCTCGATCTCGAACTCGTGGCCGTTCACGGTGCCCTCCATGCGCACCTTGAAGCGCATGAACTCGGTGATGACGTTCTCGGAGGAGGCCATGGTTTCGATCCACTTTCTTACAAATTTCTCTGAAGTTGTATCCTCAGTACTTCAAAGAAAATAGCTTACACCAATTTTTTCTTGTTTTCACAAATGCCGAACTTGGTTCCTTATATAGGAAAACTCAAGGGCAAAAATGACACGGAAAAATATAAAAGGATAAGTAGTGGGGGATAAGATTCCTTTGTGATAAGGTTACTTTCCGCCCTTACATTTTCCACCTTACATGTGTCCTCTATGTCTCTTTCACAATCACCGACCTTATCTTCTTCTTTTCATTGTTGTCGTCAGTGCTTACGTCTTCAAGATTCTTTTCTTCGCCTGGTTCTTCTTTTTCAATTTCTACGTATTCTTCTTCGTATTCTGGCAGTATAGGATCTTGTATCTGTACATTCTTCATTTTTGAACATAGGTTGCATATGTGCCGCATATTGATCTGCTTCTTGCTGAGCTCACATAATACTTCCATAGTTTTTCCCGTAAACATTGGATTCTTGATGCTACATCTTGGATAATTACCTTCTACTAGTCG

**DNA sequence of binary vector containing Cas9/sgRNA genes targeting both the Arabidopsis and the Camelina FAD2 F1 site (*AvaI*)**

AATTCCATGGAGTCAAAGATTCAAATAGAGGACCTAACAGAACTCGCCGTAAAGACTGGCGAACAGTTCATACAGAGTCTCTTACGACTCAATGACAAGAAGAAAATCTTCGTCAACATGGTGGAGCACGACACACTTGTCTACTCCAAAAATATCAAAGATACAGTCTCAGAAGACCAAAGGGCAATTGAGACTTTTCAACAAAGGGTAATATCCGGAAACCTCCTCGGATTCCATTGCCCAGCTATCTGTCACTTTATTGTGAAGATAGTGGAAAAGGAAGGTGGCTCCTACAAATGCCATCATTGCGATAAAGGAAAGGCCATCGTTGAAGATGCCTCTGCCGACAGTGGTCCCAAAGATGGACCCCCACCCACGAGGAGCATCGTGGAAAAAGAAGACGTTCCAACCACGTCTTCAAAGCAAGTGGATTGATGTGATATCTCCACTGACGTAAGGGATGACGCACAATCCCACTATCCTTCGCAAGACCCTTCCTCTATATAAGGAAGTTCATTTCATTTGGAGAGAACACGGGGGACTCTTGACCATGGTAGATCTGACTAGTGATTACAAGGACGATGATGACAAGAAAGACTATAAAGATGACGATGATAAGCATATGGACAAGAAGTACAGCATCGGCCTGGACATCGGCACGAACTCGGTGGGCTGGGCGGTGATCACGGACGAGTACAAGGTGCCCTCCAAGAAGTTCAAGGTGCTGGGCAACACCGACCGCCACTCGATCAAGAAGAACCTGATCGGCGCCCTGCTGTTCGACTCCGGCGAGACCGCCGAGGCGACGCGCCTGAAGCGCACCGCGCGTCGCCGCTACACGCGTCGCAAGAACCGCATCTGCTACCTGCAGGAGATCTTCAGCAACGAGATGGCCAAGGTGGACGACTCGTTCTTCCACCGCCTGGAGGAGTCCTTCCTGGTGGAGGAAGACAAGAAGCACGAGCGCCACCCCATCTTCGGCAACATCGTGGACGAGGTGGCCTACCACGAGAAGTACCCGACGATCTACCACCTGCGCAAGAAGCTGGTGGACAGCACCGACAAGGCGGACCTGCGCCTGATCTACCTGGCCCTGGCGCACATGATCAAGTTCCGCGGCCACTTCCTGATCGAGGGCGACCTGAACCCCGACAACTCGGACGTGGACAAGCTGTTCATCCAGCTGGTGCAGACCTACAACCAGCTGTTCGAGGAGAACCCGATCAACGCCTCCGGCGTGGACGCCAAGGCGATCCTGAGCGCGCGCCTGTCCAAGAGCCGTCGCCTGGAGAACCTGATCGCCCAGCTGCCCGGCGAGAAGAAGAACGGCCTGTTCGGCAACCTGATCGCGCTGTCGCTGGGCCTGACGCCGAACTTCAAGTCCAACTTCGACCTGGCCGAGGACGCGAAGCTGCAGCTGAGCAAGGACACCTACGACGACGACCTGGACAACCTGCTGGCCCAGATCGGCGACCAGTACGCGGACCTGTTCCTGGCCGCGAAGAACCTGTCGGACGCCATCCTGCTGTCCGACATCCTGCGCGTGAACACCGAGATCACGAAGGCCCCCCTGTCGGCGTCCATGATCAAGCGCTACGACGAGCACCACCAGGACCTGACCCTGCTGAAGGCGCTGGTGCGCCAGCAGCTGCCGGAGAAGTACAAGGAGATCTTCTTCGACCAGAGCAAGAACGGCTACGCCGGCTACATCGACGGCGGCGCGTCGCAAGAGGAGTTCTACAAGTTCATCAAGCCCATCCTGGAGAAGATGGACGGCACGGAGGAGCTGCTGGTGAAGCTGAACCGCGAGGACCTGCTGCGCAAGCAGCGCACCTTCGACAACGGCAGCATCCCCCACCAGATCCACCTGGGCGAGCTGCACGCCATCCTGCGTCGCCAAGAGGACTTCTACCCGTTCCTGAAGGACAACCGCGAGAAGATCGAGAAGATCCTGACGTTCCGCATCCCCTACTACGTGGGCCCGCTGGCCCGCGGCAACAGCCGCTTCGCGTGGATGACCCGCAAGTCGGAGGAGACCATCACGCCCTGGAACTTCGAGGAAGTGGTGGACAAGGGCGCCAGCGCGCAGTCGTTCATCGAGCGCATGACCAACTTCGACAAGAACCTGCCCAACGAGAAGGTGCTGCCGAAGCACTCCCTGCTGTACGAGTACTTCACCGTGTACAACGAGCTGACGAAGGTGAAGTACGTGACCGAGGGCATGCGCAAGCCCGCCTTCCTGAGCGGCGAGCAGAAGAAGGCGATCGTGGACCTGCTGTTCAAGACCAACCGCAAGGTGACGGTGAAGCAGCTGAAAGAGGACTACTTCAAGAAGATCGAGTGCTTCGACAGCGTGGAGATCTCGGGCGTGGAGGACCGCTTCAACGCCAGCCTGGGCACCTACCACGACCTGCTGAAGATCATCAAGGACAAGGACTTCCTGGACAACGAGGAGAACGAGGACATCCTGGAGGACATCGTGCTGACCCTGACGCTGTTCGAGGACCGCGAGATGATCGAGGAGCGCCTGAAGACGTACGCCCACCTGTTCGACGACAAGGTGATGAAGCAGCTGAAGCGTCGCCGCTACACCGGCTGGGGCCGCCTGAGCCGCAAGCTGATCAACGGCATCCGCGACAAGCAGTCCGGCAAGACCATCCTGGACTTCCTGAAGAGCGACGGCTTCGCGAACCGCAACTTCATGCAGCTGATCCACGACGACTCGCTGACCTTCAAAGAGGACATCCAGAAGGCCCAGGTGTCGGGCCAGGGCGACTCCCTGCACGAGCACATCGCCAACCTGGCGGGCTCCCCCGCGATCAAGAAGGGCATCCTGCAGACCGTGAAGGTGGTGGACGAGCTGGTGAAGGTGATGGGCCGCCACAAGCCGGAGAACATCGTGATCGAGATGGCCCGCGAGAACCAGACCACGCAGAAGGGCCAGAAGAACAGCCGCGAGCGCATGAAGCGCATCGAGGAAGGCATCAAGGAGCTGGGCTCGCAGATCCTGAAGGAGCACCCCGTGGAGAACACCCAGCTGCAGAACGAGAAGCTGTACCTGTACTACCTGCAGAACGGCCGCGACATGTACGTGGACCAGGAGCTGGACATCAACCGCCTGTCCGACTACGACGTGGACCACATCGTGCCCCAGAGCTTCCTGAAGGACGACTCGATCGACAACAAGGTGCTGACCCGCAGCGACAAGAACCGCGGCAAGAGCGACAACGTGCCGTCGGAGGAAGTGGTGAAGAAGATGAAGAACTACTGGCGCCAGCTGCTGAACGCCAAGCTGATCACGCAGCGCAAGTTCGACAACCTGACCAAGGCCGAGCGCGGTGGCCTGTCGGAGCTGGACAAGGCGGGCTTCATCAAGCGCCAGCTGGTGGAGACCCGCCAGATCACGAAGCACGTGGCGCAGATCCTGGACTCCCGCATGAACACGAAGTACGACGAGAACGACAAGCTGATCCGCGAGGTGAAGGTGATCACCCTGAAGTCCAAGCTGGTCAGCGACTTCCGCAAGGACTTCCAGTTCTACAAGGTGCGCGAGATCAACAACTACCACCACGCCCACGACGCGTACCTGAACGCCGTGGTGGGCACCGCGCTGATCAAGAAGTACCCCAAGCTGGAGAGCGAGTTCGTGTACGGCGACTACAAGGTGTACGACGTGCGCAAGATGATCGCCAAGTCGGAGCAGGAGATCGGCAAGGCCACCGCGAAGTACTTCTTCTACTCCAACATCATGAACTTCTTCAAGACCGAGATCACGCTGGCCAACGGCGAGATCCGCAAGCGCCCGCTGATCGAGACCAACGGCGAGACGGGCGAGATCGTGTGGGACAAGGGCCGCGACTTCGCGACCGTGCGCAAGGTGCTGAGCATGCCCCAGGTGAACATCGTGAAGAAGACCGAGGTGCAGACGGGCGGCTTCTCCAAGGAGAGCATCCTGCCGAAGCGCAACTCGGACAAGCTGATCGCCCGCAAGAAGGACTGGGACCCCAAGAAGTACGGCGGCTTCGACTCCCCGACCGTGGCCTACAGCGTGCTGGTGGTGGCGAAGGTGGAGAAGGGCAAGTCCAAGAAGCTGAAGAGCGTGAAGGAGCTGCTGGGCATCACCATCATGGAGCGCAGCTCGTTCGAGAAGAACCCCATCGACTTCCTGGAGGCCAAGGGCTACAAAGAGGTGAAGAAGGACCTGATCATCAAGCTGCCGAAGTACTCGCTGTTCGAGCTGGAGAACGGCCGCAAGCGCATGCTGGCCTCCGCGGGCGAGCTGCAGAAGGGCAACGAGCTGGCCCTGCCCAGCAAGTACGTGAACTTCCTGTACCTGGCGTCCCACTACGAGAAGCTGAAGGGCTCGCCGGAGGACAACGAGCAGAAGCAGCTGTTCGTGGAGCAGCACAAGCACTACCTGGACGAGATCATCGAGCAGATCTCGGAGTTCTCCAAGCGCGTGATCCTGGCCGACGCGAACCTGGACAAGGTGCTGAGCGCCTACAACAAGCACCGCGACAAGCCCATCCGCGAGCAGGCGGAGAACATCATCCACCTGTTCACCCTGACGAACCTGGGCGCCCCGGCCGCGTTCAAGTACTTCGACACCACGATCGACCGCAAGCGCTACACCTCCACGAAAGAGGTGCTGGACGCGACCCTGATCCACCAGAGCATCACCGGCCTGTACGAGACGCGCATCGACCTGAGCCAGCTGGGCGGCGACTCCCGCGCGGACCCGAAGAAGAAGCGCAAGGTGTAAGAATTAATTCGGATCGATCCATTGGTGACCAGCTCGAATTTCCCCGATCGTTCAAACATTTGGCAATAAAGTTTCTTAAGATTGAATCCTGTTGCCGGTCTTGCGATGATTATCATATAATTTCTGTTGAATTACGTTAAGCATGTAATAATTAACATGTAATGCATGACGTTATTTATGAGATGGGTTTTTATGATTAGAGTCCCGCAATTATACATTTAATACGCGATAGAAAACAAAATATAGCGCGCAAACTAGGATAAATTATCGCGCGCGGTGTCATCTATGTTACTAGATCGGGGGTACCCGTTGAACAACGGAAACTCGACTTGCCTTCCGCACAATACATCATTTCTTCTTAGCTTTTTTTCTTCTTCTTCGTTCATACAGTTTTTTTTTGTTTATCAGCTTACATTTTCTTGAACCGTAGCTTTCGTTTTCTTCTTTTTAACTTTCCATTCGGAGTTTTTGTATCTTGTTTCATAGTTTGTCCCAGGATTAGAATGATTAGGCATCGAACCTTCAAGAATTTGATTGAATAAAACATCTTCATTCTTAAGATATGAAGATAATCTTCAAAAGGCCCCTGGGAATCTGAAAGAAGAGAAGCAGGCCCATTTATATGGGAAAGAACAATAGTATTTCTTATATAGGCCCATTTAAGTTGAAAACAATCTTCAAAAGTCCCACATCGCTTAGATAAGAAAACGAAGCTGAGTTTATATACAGCTAGAGTCGAAGTAGTGATTGTCCAGTTTGTCCTCGGGGTTTTAGAGCTAGAAATAGCAAGTTAAAATAAGGCTAGTCCGTTATCAACTTGAAAAAGTGGCACCGAGTCGGTGCTTTTTTTTTTTGCAAAATTTTCCAGATCGATTTCTTCTTCCTCTGTTCTTCGGCGTTCAATTTCTGGGTTTTTCTCTTCGTTTTCTGTAACTGAAACCTAAAATTTGACCTAAAAAAAATCTCAAATAATATGATTCAGTGGTTTTGTACTTTTCAGTTAGTTGAGTTTTGCAGTTCCGATGAGATAAACCAATAACTTTGCTTAGATCTAATTCATTCCGTTACACCTCTGATGGAGATGGAAGGTTCTTAATAATGATGCCATTTTTTGGGTAATAATTTTGAATTAGAATCAAGGGTATAAGATTCATAATTAACATCACTTAAGCAAAGTTCGTAATATACGACCACAGGATATAATTTTTGGTCTAGAGTCGACCTGCAGGCATGCAAGCTTGGCACTGGCCGTCGTTTTACAACGTCGTGACTGGGAAAACCCTGGCGTTACCCAACTTAATCGCCTTGCAGCACATCCCCCTTTCGCCAGCTGGCGTAATAGCGAAGAGGCCCGCACCGATCGCCCTTCCCAACAGTTGCGCAGCCTGAATGGCGAATGCTAGAGCAGCTTGAGCTTGGATCAGATTGTCGTTTCCCGCCTTCAGTTTAAACTATCAGTGTTTGACAGGATATATTGGCGGGTAAACCTAAGAGAAAAGAGCGTTTATTAGAATAACGGATATTTAAAAGGGCGTGAAAAGGTTTATCCGTTCGTCCATTTGTATGTGCATGCCAACCACAGGGTTCCCCTCGGGATCAAAGTACTTTGATCCAACCCCTCCGCTGCTATAGTGCAGTCGGCTTCTGACGTTCAGTGCAGCCGTCTTCTGAAAACGACATGTCGCACAAGTCCTAAGTTACGCGACAGGCTGCCGCCCTGCCCTTTTCCTGGCGTTTTCTTGTCGCGTGTTTTAGTCGCATAAAGTAGAATACTTGCGACTAGAACCGGAGACATTACGCCATGAACAAGAGCGCCGCCGCTGGCCTGCTGGGCTATGCCCGCGTCAGCACCGACGACCAGGACTTGACCAACCAACGGGCCGAACTGCACGCGGCCGGCTGCACCAAGCTGTTTTCCGAGAAGATCACCGGCACCAGGCGCGACCGCCCGGAGCTGGCCAGGATGCTTGACCACCTACGCCCTGGCGACGTTGTGACAGTGACCAGGCTAGACCGCCTGGCCCGCAGCACCCGCGACCTACTGGACATTGCCGAGCGCATCCAGGAGGCCGGCGCGGGCCTGCGTAGCCTGGCAGAGCCGTGGGCCGACACCACCACGCCGGCCGGCCGCATGGTGTTGACCGTGTTCGCCGGCATTGCCGAGTTCGAGCGTTCCCTAATCATCGACCGCACCCGGAGCGGGCGCGAGGCCGCCAAGGCCCGAGGCGTGAAGTTTGGCCCCCGCCCTACCCTCACCCCGGCACAGATCGCGCACGCCCGCGAGCTGATCGACCAGGAAGGCCGCACCGTGAAAGAGGCGGCTGCACTGCTTGGCGTGCATCGCTCGACCCTGTACCGCGCACTTGAGCGCAGCGAGGAAGTGACGCCCACCGAGGCCAGGCGGCGCGGTGCCTTCCGTGAGGACGCATTGACCGAGGCCGACGCCCTGGCGGCCGCCGAGAATGAACGCCAAGAGGAACAAGCATGAAACCGCACCAGGACGGCCAGGACGAACCGTTTTTCATTACCGAAGAGATCGAGGCGGAGATGATCGCGGCCGGGTACGTGTTCGAGCCGCCCGCGCACGTCTCAACCGTGCGGCTGCATGAAATCCTGGCCGGTTTGTCTGATGCCAAGCTGGCGGCCTGGCCGGCCAGCTTGGCCGCTGAAGAAACCGAGCGCCGCCGTCTAAAAAGGTGATGTGTATTTGAGTAAAACAGCTTGCGTCATGCGGTCGCTGCGTATATGATGCGATGAGTAAATAAACAAATACGCAAGGGGAACGCATGAAGGTTATCGCTGTACTTAACCAGAAAGGCGGGTCAGGCAAGACGACCATCGCAACCCATCTAGCCCGCGCCCTGCAACTCGCCGGGGCCGATGTTCTGTTAGTCGATTCCGATCCCCAGGGCAGTGCCCGCGATTGGGCGGCCGTGCGGGAAGATCAACCGCTAACCGTTGTCGGCATCGACCGCCCGACGATTGACCGCGACGTGAAGGCCATCGGCCGGCGCGACTTCGTAGTGATCGACGGAGCGCCCCAGGCGGCGGACTTGGCTGTGTCCGCGATCAAGGCAGCCGACTTCGTGCTGATTCCGGTGCAGCCAAGCCCTTACGACATATGGGCCACCGCCGACCTGGTGGAGCTGGTTAAGCAGCGCATTGAGGTCACGGATGGAAGGCTACAAGCGGCCTTTGTCGTGTCGCGGGCGATCAAAGGCACGCGCATCGGCGGTGAGGTTGCCGAGGCGCTGGCCGGGTACGAGCTGCCCATTCTTGAGTCCCGTATCACGCAGCGCGTGAGCTACCCAGGCACTGCCGCCGCCGGCACAACCGTTCTTGAATCAGAACCCGAGGGCGACGCTGCCCGCGAGGTCCAGGCGCTGGCCGCTGAAATTAAATCAAAACTCATTTGAGTTAATGAGGTAAAGAGAAAATGAGCAAAAGCACAAACACGCTAAGTGCCGGCCGTCCGAGCGCACGCAGCAGCAAGGCTGCAACGTTGGCCAGCCTGGCAGACACGCCAGCCATGAAGCGGGTCAACTTTCAGTTGCCGGCGGAGGATCACACCAAGCTGAAGATGTACGCGGTACGCCAAGGCAAGACCATTACCGAGCTGCTATCTGAATACATCGCGCAGCTACCAGAGTAAATGAGCAAATGAATAAATGAGTAGATGAATTTTAGCGGCTAAAGGAGGCGGCATGGAAAATCAAGAACAACCAGGCACCGACGCCGTGGAATGCCCCATGTGTGGAGGAACGGGCGGTTGGCCAGGCGTAAGCGGCTGGGTTGTCTGCCGGCCCTGCAATGGCACTGGAACCCCCAAGCCCGAGGAATCGGCGTGACGGTCGCAAACCATCCGGCCCGGTACAAATCGGCGCGGCGCTGGGTGATGACCTGGTGGAGAAGTTGAAGGCCGCGCAGGCCGCCCAGCGGCAACGCATCGAGGCAGAAGCACGCCCCGGTGAATCGTGGCAAGCGGCCGCTGATCGAATCCGCAAAGAATCCCGGCAACCGCCGGCAGCCGGTGCGCCGTCGATTAGGAAGCCGCCCAAGGGCGACGAGCAACCAGATTTTTTCGTTCCGATGCTCTATGACGTGGGCACCCGCGATAGTCGCAGCATCATGGACGTGGCCGTTTTCCGTCTGTCGAAGCGTGACCGACGAGCTGGCGAGGTGATCCGCTACGAGCTTCCAGACGGGCACGTAGAGGTTTCCGCAGGGCCGGCCGGCATGGCCAGTGTGTGGGATTACGACCTGGTACTGATGGCGGTTTCCCATCTAACCGAATCCATGAACCGATACCGGGAAGGGAAGGGAGACAAGCCCGGCCGCGTGTTCCGTCCACACGTTGCGGACGTACTCAAGTTCTGCCGGCGAGCCGATGGCGGAAAGCAGAAAGACGACCTGGTAGAAACCTGCATTCGGTTAAACACCACGCACGTTGCCATGCAGCGTACGAAGAAGGCCAAGAACGGCCGCCTGGTGACGGTATCCGAGGGTGAAGCCTTGATTAGCCGCTACAAGATCGTAAAGAGCGAAACCGGGCGGCCGGAGTACATCGAGATCGAGCTAGCTGATTGGATGTACCGCGAGATCACAGAAGGCAAGAACCCGGACGTGCTGACGGTTCACCCCGATTACTTTTTGATCGATCCCGGCATCGGCCGTTTTCTCTACCGCCTGGCACGCCGCGCCGCAGGCAAGGCAGAAGCCAGATGGTTGTTCAAGACGATCTACGAACGCAGTGGCAGCGCCGGAGAGTTCAAGAAGTTCTGTTTCACCGTGCGCAAGCTGATCGGGTCAAATGACCTGCCGGAGTACGATTTGAAGGAGGAGGCGGGGCAGGCTGGCCCGATCCTAGTCATGCGCTACCGCAACCTGATCGAGGGCGAAGCATCCGCCGGTTCCTAATGTACGGAGCAGATGCTAGGGCAAATTGCCCTAGCAGGGGAAAAAGGTCGAAAAGGTCTCTTTCCTGTGGATAGCACGTACATTGGGAACCCAAAGCCGTACATTGGGAACCGGAACCCGTACATTGGGAACCCAAAGCCGTACATTGGGAACCGGTCACACATGTAAGTGACTGATATAAAAGAGAAAAAAGGCGATTTTTCCGCCTAAAACTCTTTAAAACTTATTAAAACTCTTAAAACCCGCCTGGCCTGTGCATAACTGTCTGGCCAGCGCACAGCCGAAGAGCTGCAAAAAGCGCCTACCCTTCGGTCGCTGCGCTCCCTACGCCCCGCCGCTTCGCGTCGGCCTATCGCGGCCGCTGGCCGCTCAAAAATGGCTGGCCTACGGCCAGGCAATCTACCAGGGCGCGGACAAGCCGCGCCGTCGCCACTCGACCGCCGGCGCCCACATCAAGGCACCCTGCCTCGCGCGTTTCGGTGATGACGGTGAAAACCTCTGACACATGCAGCTCCCGGAGACGGTCACAGCTTGTCTGTAAGCGGATGCCGGGAGCAGACAAGCCCGTCAGGGCGCGTCAGCGGGTGTTGGCGGGTGTCGGGGCGCAGCCATGACCCAGTCACGTAGCGATAGCGGAGTGTATACTGGCTTAACTATGCGGCATCAGAGCAGATTGTACTGAGAGTGCACCATATGCGGTGTGAAATACCGCACAGATGCGTAAGGAGAAAATACCGCATCAGGCGCTCTTCCGCTTCCTCGCTCACTGACTCGCTGCGCTCGGTCGTTCGGCTGCGGCGAGCGGTATCAGCTCACTCAAAGGCGGTAATACGGTTATCCACAGAATCAGGGGATAACGCAGGAAAGAACATGTGAGCAAAAGGCCAGCAAAAGGCCAGGAACCGTAAAAAGGCCGCGTTGCTGGCGTTTTTCCATAGGCTCCGCCCCCCTGACGAGCATCACAAAAATCGACGCTCAAGTCAGAGGTGGCGAAACCCGACAGGACTATAAAGATACCAGGCGTTTCCCCCTGGAAGCTCCCTCGTGCGCTCTCCTGTTCCGACCCTGCCGCTTACCGGATACCTGTCCGCCTTTCTCCCTTCGGGAAGCGTGGCGCTTTCTCATAGCTCACGCTGTAGGTATCTCAGTTCGGTGTAGGTCGTTCGCTCCAAGCTGGGCTGTGTGCACGAACCCCCCGTTCAGCCCGACCGCTGCGCCTTATCCGGTAACTATCGTCTTGAGTCCAACCCGGTAAGACACGACTTATCGCCACTGGCAGCAGCCACTGGTAACAGGATTAGCAGAGCGAGGTATGTAGGCGGTGCTACAGAGTTCTTGAAGTGGTGGCCTAACTACGGCTACACTAGAAGGACAGTATTTGGTATCTGCGCTCTGCTGAAGCCAGTTACCTTCGGAAAAAGAGTTGGTAGCTCTTGATCCGGCAAACAAACCACCGCTGGTAGCGGTGGTTTTTTTGTTTGCAAGCAGCAGATTACGCGCAGAAAAAAAGGATCTCAAGAAGATCCTTTGATCTTTTCTACGGGGTCTGACGCTCAGTGGAACGAAAACTCACGTTAAGGGATTTTGGTCATGCATTCTAGGTACTAAAACAATTCATCCAGTAAAATATAATATTTTATTTTCTCCCAATCAGGCTTGATCCCCAGTAAGTCAAAAAATAGCTCGACATACTGTTCTTCCCCGATATCCTCCCTGATCGACCGGACGCAGAAGGCAATGTCATACCACTTGTCCGCCCTGCCGCTTCTCCCAAGATCAATAAAGCCACTTACTTTGCCATCTTTCACAAAGATGTTGCTGTCTCCCAGGTCGCCGTGGGAAAAGACAAGTTCCTCTTCGGGCTTTTCCGTCTTTAAAAAATCATACAGCTCGCGCGGATCTTTAAATGGAGTGTCTTCTTCCCAGTTTTCGCAATCCACATCGGCCAGATCGTTATTCAGTAAGTAATCCAATTCGGCTAAGCGGCTGTCTAAGCTATTCGTATAGGGACAATCCGATATGTCGATGGAGTGAAAGAGCCTGATGCACTCCGCATACAGCTCGATAATCTTTTCAGGGCTTTGTTCATCTTCATACTCTTCCGAGCAAAGGACGCCATCGGCCTCACTCATGAGCAGATTGCTCCAGCCATCATGCCGTTCAAAGTGCAGGACCTTTGGAACAGGCAGCTTTCCTTCCAGCCATAGCATCATGTCCTTTTCCCGTTCCACATCATAGGTGGTCCCTTTATACCGGCTGTCCGTCATTTTTAAATATAGGTTTTCATTTTCTCCCACCAGCTTATATACCTTAGCAGGAGACATTCCTTCCGTATCTTTTACGCAGCGGTATTTTTCGATCAGTTTTTTCAATTCCGGTGATATTCTCATTTTAGCCATTTATTATTTCCTTCCTCTTTTCTACAGTATTTAAAGATACCCCAAGAAGCTAATTATAACAAGACGAACTCCAATTCACTGTTCCTTGCATTCTAAAACCTTAAATACCAGAAAACAGCTTTTTCAAAGTTGTTTTCAAAGTTGGCGTATAACATAGTATCGACGGAGCCGATTTTGAAACCGCGGTGATCACAGGCAGCAACGCTCTGTCATCGTTACAATCAACATGCTACCCTCCGCGAGATCATCCGTGTTTCAAACCCGGCAGCTTAGTTGCCGTTCTTCCGAATAGCATCGGTAACATGAGCAAAGTCTGCCGCCTTACAACGGCTCTCCCGCTGACGCCGTCCCGGACTGATGGGCTGCCTGTATCGAGTGGTGATTTTGTGCCGAGCTGCCGGTCGGGGAGCTGTTGGCTGGCTGGTGGCAGGATATATTGTGGTGTAAACAAATTGACGCTTAGACAACTTAATAACACATTGCGGACGTTTTTAATGTACTGAATTAACGCCGAATTAATTCGGGGGATCTGGATTTTAGTACTGGATTTTGGTTTTAGGAATTAGAAATTTTATTGATAGAAGTATTTTACAAATACAAATACATACTAAGGGTTTCTTATATGCTCAACACATGAGCGAAACCCTATAGGAACCCTAATTCCCTTATCTGGGAACTACTCACACATTATTATGGAGAAACTCGAGCTTGTCGATCGACAGATCCTACAGGAACAGGTGGTGGCGGCCCTCGGTGCGCTCGTACTGCTCCACGATGGTGTAGTCCTCGTTGTGGGAGGTGATGTCCAGCTTGGCGTCCACGTAGTAGTAGCCGGGCAGCTGCACGGGCTTCTTGGCCATGTAGATGGACTTGAACTCCACCAGGTAGTGGCCGCCGTCCTTCAGCTTCAGGGCCTTGTGGGTCTCGCCCTTCAGCACGCCGTCGCGGGGGTACAGGCGCTCGGTGGAGGCCTCCCAGCCCATGGTCTTCTTCTGCATCACGGGGCCGTCGGAGGGGAAGTTCACGCCGATGAACTTCACCTTGTAGATGAAGCAGCCGTCCTGCAGGGAGGAGTCCTGGGTCACGGTCGCCACGCCGCCGTCCTCGAAGTTCATCACGCGCTCCCACTTGAAGCCCTCGGGGAAGGACAGCTTCTTGTAGTCGGGGATGTCGGCGGGGTGCTTCACGTACACCTTGGAGCCGTACTGGAACTGGGGGGACAGGATGTCCCAGGCGAAGGGCAGGGGGCCGCCCTTGGTCACCTTCAGCTTCACGGTGTTGTGGCCCTCGTAGGGGCGGCCCTCGCCCTCGCCCTCGATCTCGAACTCGTGGCCGTTCACGGTGCCCTCCATGCGCACCTTGAAGCGCATGAACTCGGTGATGACGTTCTCGGAGGAGGCCATGGTTTCGATCCACTTTCTTACAAATTTCTCTGAAGTTGTATCCTCAGTACTTCAAAGAAAATAGCTTACACCAATTTTTTCTTGTTTTCACAAATGCCGAACTTGGTTCCTTATATAGGAAAACTCAAGGGCAAAAATGACACGGAAAAATATAAAAGGATAAGTAGTGGGGGATAAGATTCCTTTGTGATAAGGTTACTTTCCGCCCTTACATTTTCCACCTTACATGTGTCCTCTATGTCTCTTTCACAATCACCGACCTTATCTTCTTCTTTTCATTGTTGTCGTCAGTGCTTACGTCTTCAAGATTCTTTTCTTCGCCTGGTTCTTCTTTTTCAATTTCTACGTATTCTTCTTCGTATTCTGGCAGTATAGGATCTTGTATCTGTACATTCTTCATTTTTGAACATAGGTTGCATATGTGCCGCATATTGATCTGCTTCTTGCTGAGCTCACATAATACTTCCATAGTTTTTCCCGTAAACATTGGATTCTTGATGCTACATCTTGGATAATTACCTTCTACTAGTCG
